# Non-skeletal health effects of vitamin D supplementation: A systematic review on findings from meta-analyses summarizing trial data

**DOI:** 10.1371/journal.pone.0180512

**Published:** 2017-07-07

**Authors:** Lars Rejnmark, Lise Sofie Bislev, Kevin D. Cashman, Gudny Eiríksdottir, Martin Gaksch, Martin Grübler, Guri Grimnes, Vilmundur Gudnason, Paul Lips, Stefan Pilz, Natasja M. van Schoor, Mairead Kiely, Rolf Jorde

**Affiliations:** 1 Department of Endocrinology and Internal Medicine, Aarhus University Hospital, Aarhus, Denmark; 2 Cork Centre for Vitamin D and Nutrition Research, School of Food and Nutritional Sciences, University College Cork, Cork, Ireland; 3 Icelandic Heart Association, Kopavogur, Iceland; 4 Division of Endocrinology and Diabetology, Department of Internal Medicine, Medical University of Graz, Graz, Austria; 5 Swiss Cardiovascular Centre Bern, Department of Cardiology, Inselspital, Bern University Hospital, University of Bern, Bern, Switzerland; 6 Tromsø Endocrine Research Group, Department of Clinical Medicine, UiT The Arctic University of Norway, Tromsø, Norway; 7 Faculty of Medicine, School of Health Sciences, University of Iceland, Reykjavik, Iceland; 8 Department of Internal Medicine, Section of Endocrinology, Vrije University Medical Center, Amsterdam, Netherlands; 9 Amsterdam Public Health Research Institute, Department of Epidemiology and Biostatistics, VU University Medical Center, Amsterdam, Netherlands; University of Alabama at Birmingham, UNITED STATES

## Abstract

**Background:**

A large number of observational studies have reported harmful effects of low 25-hydroxyvitamin D (25OHD) levels on non-skeletal outcomes. We performed a systematic quantitative review on characteristics of randomized clinical trials (RCTs) included in meta-analyses (MAs) on non-skeletal effects of vitamin D supplementation.

**Methods and findings:**

We identified systematic reviews (SR) reporting summary data in terms of MAs of RCTs on selected non-skeletal outcomes. For each outcome, we summarized the results from available SRs and scrutinized included RCTs for a number of predefined characteristics. We identified 54 SRs including data from 210 RCTs. Most MAs as well as the individual RCTs reported null-findings on risk of cardiovascular diseases, type 2 diabetes, weight-loss, and malignant diseases. Beneficial effects of vitamin D supplementation was reported in 1 of 4 MAs on depression, 2 of 9 MAs on blood pressure, 3 of 7 MAs on respiratory tract infections, and 8 of 12 MAs on mortality. Most RCTs have primarily been performed to determine skeletal outcomes, whereas non-skeletal effects have been assessed as secondary outcomes. Only one-third of the RCTs had low level of 25OHD as a criterion for inclusion and a mean baseline 25OHD level below 50 nmol/L was only present in less than half of the analyses.

**Conclusions:**

Published RCTs have mostly been performed in populations without low 25OHD levels. The fact that most MAs on results from RCTs did not show a beneficial effect does not disprove the hypothesis suggested by observational findings on adverse health outcomes of low 25OHD levels.

## Introduction

In recent years the number of studies exploring effects of vitamin D beyond its well-known effects on the musculo-skeletal system have increased markedly. The vitamin D receptor (VDR) and the enzyme (the 1α-hydroxylase) needed to hydroxylate 25-hydroxyvitamin D (25OHD) to its active form 1,25-dihydroxyvitamin D (1,25(OH)2D) has been identified in a large number of different cells. This widespread expression of the 1α-hydroxylase suggests that local production and action of 1,25(OH)2D to regulate VDR-directed gene expression may be of importance to the function of many tissues [1]. Moreover, gene array studies have shown that vitamin D may be involved in the regulation of as much as 5% of the human genome [1–3].

Studies in different populations from around the world have shown a high prevalence of vitamin D insufficiency, and observational studies have described associations between low circulating levels of 25OHD and a large number of diseases, including cardiovascular diseases (CVD), malignancies, diabetes, obesity, infections, neuropsychiatric, and autoimmune diseases [4–16].

As causality should not be infered from observational studies, there is a strong need for clinical and population based trials. So far, only relatively few randomized clinical trials (RCT), specifically designed to assess effects of vitamin D on non-skeletal outcomes, have been performed. Nevertheless, the number of papers published on potential causal effects of vitamin D supplementation is rapidly growing as findings from previously published RCTs, originally designed to evaluate skeletal effects, are being reanalysed in order to elucidate possible non-skeletal outcomes. In evidence-based medicine, results from systematic reviews (SR) of RCTs are considered as the highest level of evidence [17]. Within the last few years, an increasingly number of SRs has been published, including meta-analyses (MAs) with summary data on results from RCT on non-skeletal outcomes in response to supplementation with vitamin D [18–20]. In this paper, our aim is to provide a comprehensive umbrella review on SRs reporting MAs with summary results on clinically relevant non-skeletal outcomes from trials on vitamin D supplementation. In addition, we report characteristics of the individual trials included in MAs, in order to review the evidence-base providing data for published MAs.

## Methods

The present paper is a part of a collaborative study between a number of European research institutions within a project on *food-based solutions for eradication of vitamin D deficiency and health promotion throughout the life cycle* (ODIN project, www.odin-vitd.eu) funded by the European Commission as part of the Seventh Framework Programme of the European Community for Research, Technological Development and Demonstration Activities.

At a consensus meeting in Tromsø, Norway, we decided which outcomes to study. Criteria for selecting studied outcomes were:

1. Non-skeletal effects of supplementation with vitamin D in terms of either vitamin D2 (ergocalciferol) or vitamin D3 (cholecalciferol) have been investigated in at least two randomized trials.
2. At least one MA published in which findings fromRCTs has been summarized.
3. Outcomes should cover different organ systems and be of clinical importance to European citizens i.e., outcomes on biomarkers were not considered.
4. Reviews should be published in English

Based on these criteria, we selected to study effects of vitamin D supplementation in trials on CVD, blood pressure, type 2 diabetes (T2D), body weight, birth weight, malignant diseases, respiratory tract infections (excluding tuberculosis), depression, and mortality.

We searched PubMed, Embase, and the Cochrane Library until December 1^st^, 2016 for SRs published in English within the last 10 years on findings from RCTs testing effects of vitamin D supplementations on the selected outcomes. The search strings used are detailed in the supplementary file (S1 File).

**Fig 1:**
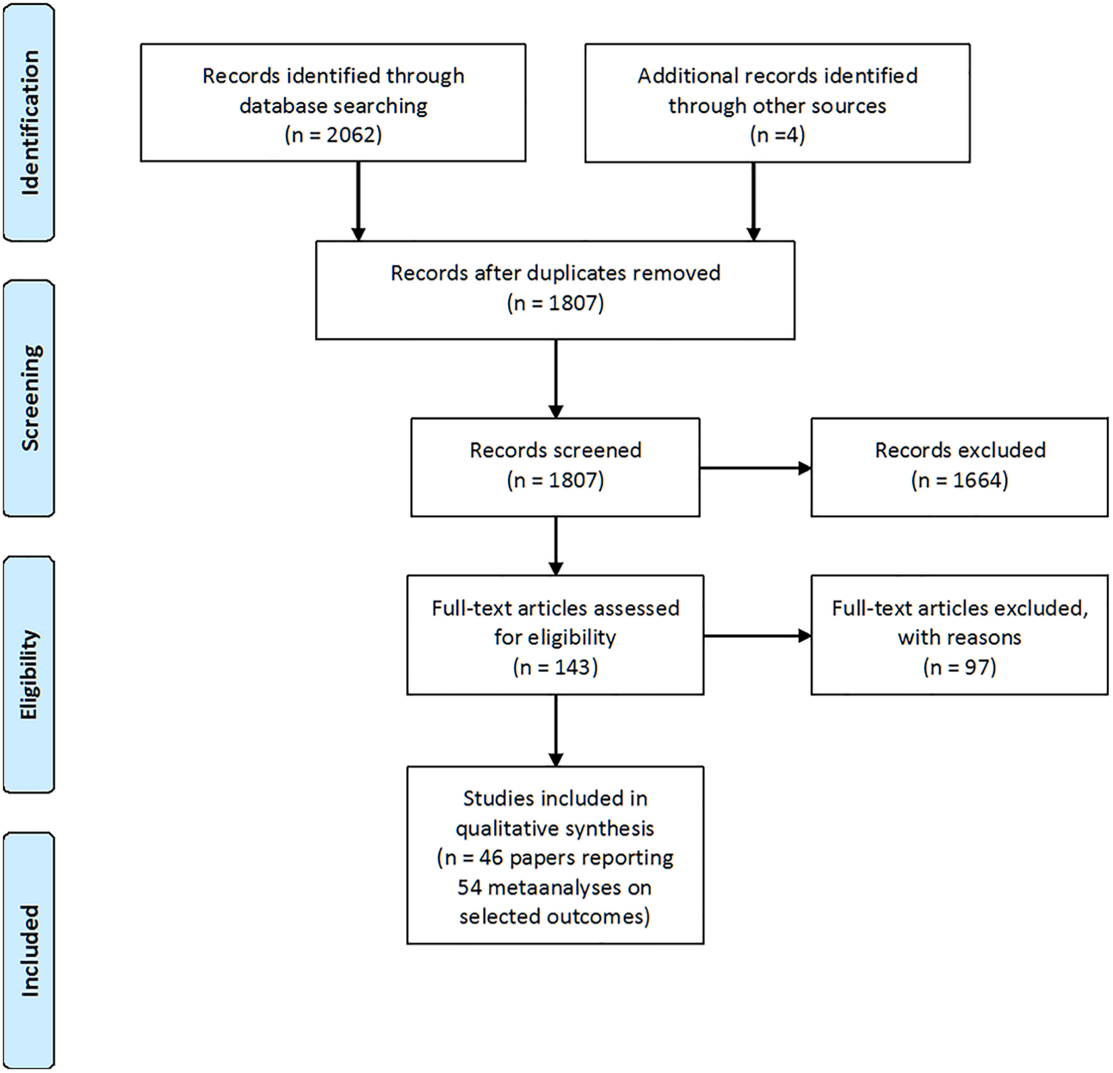
PRISMA flow diagram.

In addition, we manually searched references cited in the papers as well as papers citing the selected papers for additional articles. We only included SRs reporting summary data in terms of MAs. Only MAs on effects of treatment with calciferol (vitamin D2 or D3) were considered. However, we also accepted MAs including RCTs on activated vitamin D analogues in their summary estimate, as long as the majority (>50%) of included studies were on calciferol. In addition, we included a trial on effects of vitamin D supplementation on birth weight although published outside the predefined time range as this SR reports important information on potential bias in a previously published trial [21]. While writing the paper, a large MA on effects of vitamin D supplementation on risk of respiratory infections was published in February 2017 [22]. Although being published after the end of the time period defined *a priori*, we decided to include this paper, as it is the largest MA published so far on vitamin D and infections. A flow chart showing the search profile is shown in Fig 1.

For each MA we assessed its quality using the AMSTAR tool for which the quality is measured on a scale between 0 to 11 [23]. A total score of 9–11 is considered as ‘good’ quality, whereas a score of 5–8 shows ‘moderate’ quality and a score of 0–4 indicates a ‘poor’ quality [24]. Furthermore, for each MA we scrutinize the original papers included in the summary estimate on studied outcomes. Specific characteristics of the individual trials were systematically identified, including the population studied and whether (if relevant) subjects were diagnosed with the disease in question. We also collected information on type of vitamin D studied, dosing frequency, duration of the intervention, and whether calcium was co-administered. Furthermore, we noted whether only participants with low 25OHD levels were included (defined as 25OHD < 50 nmol/L). Finally, we retrieved information on baseline 25OHD levels and whether the intervention resulted in a more than 50% increase in 25OHD levels, as well as the main finding (conclusion) of the trial. We did not perform new MAs, but summarized the characteristics in tables in order to allow for a quantitative evaluation of characteristics of the studies forming the evidence base for published MAs.

## Results

We identified 54 SRs reporting summary data in terms of a formal MA on effects of vitamin D supplementation on selected outcomes (Table 1). Included SRs had a mean AMSTAR score of 8.8 (range 6–11) suggesting an overall moderate to good quality. Within each group of studied non-skeletal health outcomes, mean AMSTAR score varied between 8.4 to 9.8 (Table 1). For each outcome, number of RCTs included in one or more of the MAs varied from four to 69 trials. A total of 210 RCTs were included in the MAs.

**Table 1:** Summary characteristics of meta-analyses and the individual RCTs included in MA on non-skeletal effects of vitamin D supplementation.

			CVD	BP	T2D	Body Weight	Birth Weight	Cancer	RTI	Dep.	Death	ALL
**Number of meta-analyses**			7	9	1	3	6	5	7	4	12	54
	*Results (main finding)*											
		Beneficial response	0	2	0	0	2	0	3	1	8	16
		Harmful	0	1	0	0	1^a)^	0	0	0	0	2
		Null finding	7	6	1	3	3	5	4	3	4	36
**AMSTAR Score, mean**			8.4	8.9	9	8.3	9.8	9.2	9.8	8.5	9.2	8.8
**Number randomized clinical trials**			21	59	4	32	14	19	30	12	59	210^b^
	*Results (main finding)*											
		Beneficial response	0	8	0	2	4	1	9	3	0	27
		Harmful	0	0	0	1	0	0	1	0	2	4
		Null finding	21	51	4	29	10	18	20	9	57	219
	*Disease studied was primary outcome*?											
		Yes	0	15	1	5	5	0	23	4	0	53
		No	21	44	3	27	9	19	7	7	59	196
		Not reported	0	0	0	0	0	0	0	1	0	1
	*Gender*											
		Only women	13	12	1	14	14	11	2	4	26	97
		Only men	0	1	0	2	0	1	1	0	1	6
		Men and women	8	46	3	16	0	7	27	8	32	147
	*Number of included participants*											
		<1000	15	58	2	31	14	13	28	9	43	213
		≥ 1000	6	1	2	1	0	6	2	3	16	37
	*Characteristics of participants*											
		Diagnosed with the disease studied	1	7	1	11	NA	0	11	3	NA	34
		Not diagnosed with the disease	20	52	3	21	NA	19	19	9	NA	143
	*Vitamin D supplements used*:											
		Vitamin D3	13	56	4	29	9	15	30	11	43	86
		Vitamin D2	2	3	0	2	4	1	0	0	12	17
		Calciferol (D2/D3) ^b)^	1	0	0	0	1	0	0	0	3	5
		Activated vitamin D	5	0	0	1	0	3	0	1	1	13
	*Frequency of vitamin D administration*											
		Single dose	0	6	0	0	3	0	1	1	2	13
		Daily	17	29	3	23	4	14	16	7	39	152
		Less than daily	4	24	1	9	7	5	13	4	18	85
	*Co-administration of calcium*											
		No	9	47	1	18	10	11	29	8	27	160
		Only to the vitamin D group	5	4	2	5	2	5	0	2	24	49
		Both vitamin D and control group	7	8	1	9	2	3	1	2	8	41
	*25OHD < 50 nmol/L as inclusion criteria*											
		No	19	8	4	26	14	18	29	11	54	183
		Yes	2	51	0	6	0	1	1	1	5	67
	*Mean 25OHD levels at baseline*											
		< 50 nmol/L	7	37	1	15	5	8	7	5	27	112
		≥ 50 nmol/L	6	18	2	11	1	9	13	3	16	79
		Not reported	8	4	1	6	8	2	10	4	16	59
	*Duration of follow-up*											
		< 1 year	1	50	1	16	NA	4	18	8	19	117
		≥ 1 year	20	9	3	16	NA	15	12	4	40	119
	*Mean changes in P-25OHD levels compared with baseline*:											
		< 50% increase	4	15	2	15	2	8	10	3	23	82
		≥ 50% increase	6	37	1	12	4	7	9	5	17	98
		Not reported	11	7	1	5	8	4	11	4	19	70

NA: not appropriate; Dep: depression.

a)Strong suspicion that data from one of the studies included in the MA were wrongly stated in the published paper (see text in section on birth weight)

b)Some RCTs were included in more than one MA, why total number of RCT within the row do not sum up to N = 210

### Cardiovascular diseases (CVDs)

A number of observational studies have reported an inverse association between 25OHD concentrations and risk of CVDs [25–27], including a greater carotid intima–medial thickness [28], peripheral arterial disease [29], and risks of cardiovascular (CV) death [30,31]. Moreover, MAs on data from observational studies have consistently found an increased risk of CVD in subjects with vitamin D insufficiency [32,33]. An effect of vitamin D on CV health is furthermore biologically plausible, as the 25OHD-1α-hydroxylase enzyme is expressed by CV tissues and the VDRs have been identified in vascular smooth muscle cells, cardiomyocytes, as well as in coronary arteries [34–36]. Given the presence of the VDR in the vascular system, vitamin D may potentially improve CV health through several biological pathways. For example, activation of the VDR has been shown to inhibit vascular smooth muscle cell proliferation, which is believed to be cardio-protective [37,38]. Moreover, a state of chronic inflammation is considered to play a key role in the initiation and progression of CVD [39,40], and several studies have shown an inverse association between 25OHD levels and markers of inflammation, suggesting that vitamin D also may protect against CVD by lowering the state of inflammation [41–43]. On the other hand, trials assessing the effects of vitamin D supplementation on arterial stiffness, a marker of cardiovascular risk, have shown ambiguous results. In a MA including data from seven RCTs involving a total of 547 participants, supplementation with vitamin D3 for 2 to 12 months showed no significant effects on changes in pulse wave velocity [44]. Nor did a MA including data from seven RCTs suggest beneficial effects of vitamin D supplementation on left ventricular function or exercise tolerance [45].

We identified 10 SR among which seven reported summary data for incident CVDs in terms of formal MAs, whereas three SRs considered the interventions and outcomes in published RCTs to be too heterogeneous for MA [36,46,47]. Table Aa in S1 File shows the seven SRs reporting pooled data on results from RCTs on effects of vitamin D supplementation on risk of CVDs [48–54]. None of the MAs showed neither beneficial nor harmful effects on estimates in response to the interventions on risk of CVDs, in terms of any CV events, myocardial infarctions (MI), stroke/ cerebrovascular disease, or CV death (Table Aa in S1 File). In several of the MAs summary risk estimates were stratified by whether vitamin D was provided alone or in combination with calcium (CaD), showing no effects of any of the interventions. Only one of the seven MAs addressed whether supplementation to individuals with low vitamin D levels may result in beneficial effects on cardiovascular health, showing no significant interaction when comparing risk estimates from studies with mean 25OHD levels below vs. above 50 nmol/L on stroke (P_interaction [Pi]_ = 0.36) or myocardial infarction (P_i_ = 0.83) [49]. Neither did interaction analyses suggest differences according to whether the intervention resulted in increased (unspecified) 25OHD levels on risk of stroke (P_i_ = 0.41) or myocardial infarction (Pi = 0.34) [49].

Numbers of RCTs included in each MA varied from two to 11 RCTs, and the total number of randomized participants included in the RCTs varied between 2,988 and 48,647. The seven MAs included data from a total of 21 RCTs (Table Ab in S1 File), among which 16 were on effects of treatment with calciferol and five on treatment with activated vitamin D analogues. Only the study by Trivedi et al. [55] was included in all seven MAs, whereas two trials were included in six of the MAs.

Summary characteristics of the 21 RCTs are shown in Table 1. None of the trials had pre-specified CVD as their primary outcome. Most of the trials included only women, and none of the RCTs studied only men. Six of the RCTs were large-scale studies with more than 1000 participants. Only one of the trials included paticipants recruited based on cardiovascular risk factors in terms of systolic hypertension [56]. Thirteen RCTs investigated effects of vitamin D3, two studied effects of vitamin D2 and one study did not report specifically whether the intervention was D2 or D3. In five studies, all included (only) in the MA by Ford et al. [53], effects of activated vitamin D analogues were investigated. In most of the studies, vitamin D was administered as a daily dose and calcium was co-administered in approximately half of the studies. Only two trials had low 25OHD levels (< 50 nmol/L) as inclusion criteria [57,58], in which 352 participants who received treatment for one-year participated [57,58]. In addition, four RCTs reported mean 25OHD levels at baseline below 50 nmol/L [56,59–61], in which approximately 8000 participants were randomized to treatment for one to five years. Among the 16 trials investigating effects of vitamin D2 or D3, only four reported an increase in 25OHD levels of more than 50% in response to the treatment. None of the included RCTs reported neither beneficial nor harmful effects on risk of CVDs in response to the interventions. While writing this review, we noticed that findings from a large RCT (one of the so called ‘mega-RCTs’) were published on effects of vitamin D treatment on risk of CVD in the general population as primary endpoint. In the study, Scragg et al [62] randomized 5110 participants (42% females) to receive placebo (n  =  2552) or vitamin D3 (n  =  2558) with an initial dose of 200,000 IU, followed a month later by monthly doses of 100,000 IU, for a median of 3.3 years. The study showed no beneficial effects of vitamin D supplementation on risk of CVD (hazard ratio [HR] 1.02; 95% confidence interval [CI] 0.87 to 1.20). The study population had a mean (SD) baseline deseasonalized 25OHD concentration of 66 (26) nmol/L and 25% of studied subjects had 25OHD levels below 50 nmol/L at baseline. Similar to the main analysis, sub-analyses on effects of vitamin D supplementation within the group of participants with 25OHD levels below 50nmo/L showed no effects of the intervention.

In summary, a discrepancy seems to exist between findings from observational studies and randomized trials on effects of vitamin D on risk of CVDs. Nevertheless, none of the RCTs included in the MAs have been designed to specifically address whether supplementation with vitamin D affects CV health and available data from secondary analyses are, as reviewed above, characterized by a high degree of heterogeneity. However, the recently published study by Scragg et al [62] with CVD as primary outcome does not support beneficial effects of vitamin D supplementation on risk of CVD. Importantly, whereas observational studies have reported adverse health effects on CV health of low 25OHD levels, only few of the RCTs published have specifically investigated effects in a population with low 25OHD levels.

### Blood pressure

A possible link between vitamin D and hypertension has been extensively investigated. Strong observational data associate low 25OHD levels with an increase in blood pressure and an increased risk of hypertension [63–65]. Moreover, a large Mendelian randomization analysis concluded that increased 25OHD levels might reduce risk of hypertension [66]. Vitamin D may control blood pressure through its regulatory effects on the renin–angiotensin–aldosterone system (RAAS) [67,68]. Vitamin D may suppress the renin biosynthesis [67], and human studies have shown increased levels of renin and angiotensin II in subjects with vitamin D deficiency [69,70]. Moreover, a positive correlation has been shown between PTH and angiotensin II/ aldosterone [68,71,72]. Trials with vitamin D supplementation have furthermore shown that vitamin D stimulates various effects on the endothelia, smooth muscle cells, and in macrophages. Such local mechanisms of action on the vessel wall have been suggested to be of importance for blood pressure regulation [73–77].

We identified nine SRs on RCTs reporting effect of vitamin D supplementation on blood pressure [46,49,78,79] [80–84] (Table Ba in S1 File). A significant reduction in SBP (range -6.18 mmHg. to -2.44 mmHg) with no effect on DBP was reported in two of the MAs each including four RCTs with approximately 400 participants [78,79]. In the study by Withham et al. [78] only participants from studies with elevated mean baseline blood pressure were included and effects on blood pressure was designated as the primary outcome. In contrast, in the MA by Wu et al. [79] studies on patients with hypertension were not considered, but the majority of included participants had, nevertheless, arterial hypertension.

No beneficial effects of vitamin D supplementation were, however, reported in two recent MAs by Beveridge et al [81] and Golzarand et al [83], including 38 and 30 RCTs, respectively. The two MAs included a meta-regression analysis on dose-response effects showing no associations between daily dose (-equivalent) of vitamin D supplementation and changes in BP. Similarly, the MA by Wu et al. (66) showed no dose-response effects. This is in contrast to the MAs published by Pittas et al. (39) showing a beneficial dose-response effect on DBP. In response to a daily dose above 1000 IU, DBP was significantly reduced compared with a daily dose below 1000 IU.

Three MAs have addressed effects of vitamin D in combination with calcium [46,79,83]. Two of the MAs showed no difference in change in SBP and DBP according to whether vitamin D was tested alone or in combination with calcium D, whereas the MA by Golzarand et al [83] found a significantly increase in SBP (3.64 mmHg, 95%CI: 3.15–4.13) and DBP (1.71 mmHg, 95%CI: 1.25–2.18) in response to treatment with CaD.

Two MAs have investigated effects of vitamin D supplementation in specific populations. By pooling results from 15 RCTs on patients with T2D, Lee et al. [84] found no effects of vitamin D on SBP, although DBP was slightly but significantly (p = 0.02) reduced (SMD –0.160 (95% CI –0.298 to –0.022) mmHg, I2 = 0%). In contrast, an increase in SBP (WMD: 0.237; 95% CI, 0.110 to 0.365) was found in a trial level MAs by Manousopoulou et al. [82], including five RCTs on adults with obesity.

Only two of the MAs have investigated whether baseline 25OHD levels are of importance. In the MA by Withham et al. [77], all four studies included reported mean 25OHD level below 50 nmol/L with a summary estimate showing a significantly decreased SBP in response to the intervention. In contrast, a trial-level meta-regression analyses in the SR by Beveridge et al [81] showed no significant effects on responses in blood pressure of baseline 25OHD levels or increases of 25OHD levels in response to supplementation.

The nine MAs included data from 59 RCTs with a total of 42,814 participants. The majority of the participants were from the WHI study, whereas the number of participants ranged from 16 to 511 in the other 58 RCTs (Table 3B in Bb S1 File). The studies have been published between 1983 and 2015. Eight of the RCTs had a duration of one-year or more and none of these studies with long duration showed a reduced BP in response to the intervention.

In 49% of the studies, vitamin D was administered as a daily dose and calcium was co-administered in 12 of the studies. Most trials used oral vitamin D3, but one study [85] reports the effect of UVB vs. UVA radiation and in one study the effect of a single dose vitamin D2 was investigated [86]. Although 25OHD levels below 50 nmol/L was only required as an inclusion criteria in eight (14%) of the studies, mean 25OHD levels at baseline were below 50 nmol/L in 63% of the trials and increased by more than 50% in response to the supplementation in almost two-third of the trials (Table 1).

Overall, eight RCTs reported a beneficial response of vitamin D on blood pressure [85–92], whereas 51 studies found no effects (Table Bb in S1 File). Among the eight studies reporting a beneficial response, two had low 25OHD levels as inclusion criteria [86,87] and six of the trials reported a mean 25OHD level at baseline below 50 nmol/L [85–87]. Furthermore, five of the studies reported a mean increase in 25OHD levels of 50% or more in response to the intervention [85,87,88,90,92]. Type of vitamin D intervention varied widely between the eight studies showing beneficial effects. Three studies used vitamin D in combination with calcium [87,89,91] and one study used UVB exposure [85]. The remaining four studies administrated vitamin D using different dosing regimens in terms of a daily dose [90], a weekly dose [92], a single high dose [86], or a 50,000 IU dose of D3 administrated twice three weeks apart [88]. The populations studied in the eight trials varied widely. Some studies included patients with mild hypertension, others pregnant women or healthy normotensive persons. In additin, as recently highlighted in a paper by Veloudi et al. [93], only a small proportion of the trials on effects of vitamin D supplementation on blood pressure have actually examined effects in patients with low vitamin D levels. Similarly, we found that only eight of 51 trials (16%) included in published MAs had a 25OHD level below 50 nmol/L as inclusion criterion.

*In summary*, two of nine published MA and eight of 59 individual RCTs included in the MAs, showed beneficial effect of vitamin D supplementation on BP, whereas one MA found an increase in SBP in obese adults in response to vitamin D supplementation. Overall, these results do not provide substantial support for the findings from the observational studies on an importance of vitamin D on blood pressure regulation. Nevertheless, as most trials on effects of vitamin D supplementation has been performed in populations without low 25OHD levels, the hypothesis raised by observational studies on adverse effects of low vitamin D levels has in reality not been tested to a large extent by trials performed so far.

### Diabetes

Numerous observational studies have shown lower 25OHD levels in patients with T2D compared with the general population, as well as an inverse association between 25OHD levels and fasting plasma glucose, impaired glucose tolerance, and HbA1c levels [94–103]. In studies on potential biological actions of vitamin D in relation to glucose homeostasis, vitamin D has been suggested to be of importance to insulin secretion and action. The pancreatic islet cells have been shown to express the VDR as well as the 1α-hydroxylase enzyme, enabling conversion of 25OHD into its active form directly by the β-cells [104,105]. In animal experimental studies, vitamin D deficiency inhibits pancreatic secretion of insulin [106] and vitamin D repletion of rats with vitamin D insufficiency has been shown to improve glucose tolerance and glucose-stimulated insulin release [107,108]. Trials in humans on potential effects of vitamin D supplementation on indices of glucose homeostasis have, however, not demonstrated a clear beneficial effect. Recent MAs on results from RCTs showed no significant improvement on indices of glucose homeostasis including HbA1c levels in those treated with vitamin D compared with placebo [19,109–111]. Restricting study subjects to patients with diabetes or impaired glucose tolerance, one of the MA showed, nevertheless, a small but significant improvement in fasting glucose levels (−0.32 mmol/L, 95% CI, −0.57, −0.07) and a small improvement in insulin resistance (SMD −0.25, 95% CI, −0.48, −0.03) [109]. As these reviews provides a comprehensive update on findings from randomized trials on effects of vitamin D on biochemical indices of glucose homeostasis, we chose to focus on clinical outcomes in terms of effects of vitamin D supplementation on risk of incident T2D.

We identified three SRs on risk of incident T2D in RCTs on vitamin D supplementation [12,112,113]. However, only one of the SRs provided a summary risk estimate based on a MA [12] (Table Ca in S1 File). The MA included data from four RCTs [114–117], and showed no effects of vitamin D supplementation on risk of incident T2D [12]. Stratification by glucose levels at baseline showed no effects on risk of progression in participants with normal glucose levels or in patients with an impaired glucose tolerance at baseline. The SR did not report findings according to baseline levels of 25OHD or changes in 25OHD levels in response to supplementation.

Table Cb in S1 File shows characteristics of the four RCTs included in the MA and characteristics of the RCTs are summarized in Table 1. Only one of the four studies had development of diabetes as a co-primary end-point [117]. This was a small study that included only 109 patients with pre-diabetes [117]. One study included patients diagnosed with pre-diabetes, whereas two studies included participants from the general population and one study included patients with a previous fracture. None of the studies had low 25OHD levels as a prerequisite for inclusion and mean 25OHD levels at baseline was only below 50 nmol/L in only one of the trials [116]. None of the individual RCTs showed beneficial effects of vitamin D supplementation on risk of incident T2D.

In summary, there is a discrepancy between findings from observational studies and RCTs on effects of vitamin D on risk of T2D. The number of trials is relatively small and the majority of subjects included in the trials did not have low 25OHD levels. Accordingly, the findings from observational studies on an increased risk among those with vitamin D insufficiency cannot be considered as being disproved by data from RCTs.

### Body weight/obesity

Obesity has been associated with low levels of 25OHD in a large number of observational studies [118–121]. This may be attributable to different mechanisms. Following endogenous synthesis or dietary intake, vitamin D is readily stored in adipose tissue [122]. As the body pool of fat is larger in obese compared with non-obese individuals, vitamin D may be diluted or sequestered in the larger body pool of fat resulting in lower plasma levels [123]. Moreover, in a study by Wortsman et al. [124], it was found that following endogenous synthesis; the release of vitamin D3 from the skin into the circulation is decreased in obesity. It has also been suggested that obese subjects are less exposed to sunlight, leading to lower 25OHD levels. This may be due to clothing habits and less involvement in outdoor activities (reduced mobility) resulting in less skin exposure [125]. Finally, a recently published bi-directional Mendelian randomization analysis of multiple cohorts showed that a higher BMI leads to lower plasma 25OHD levels whereas low 25OHD levels did not appear to lead to a high BMI [126].

In contrast to the above reviewed findings, suggesting that low vitamin D levels are a consequence of obesity, findings from a number of other studies have suggested that low 25OHD levels may predispose to obesity. The VDR is expressed by adipose tissue and adipose tissue has the ability to synthesize 1,25(OH)_2_D [127,128]. Studies have also suggested that vitamin D may regulate adipose tissue mass, differentiation and metabolism in ways that might contribute to obesity [129]. Furthermore, secondary hyperparathyroidism is a well-known consequence of vitamin D insufficiency and increased PTH levels promote calcium influx into the adipocytes. In adipocytes, intracellular calcium may enhance lipogenesis, and PTH excess may thereby promote weight gain [130,131]. Conflicting results have been reported on the potential anti-inflammatory effects of vitamin D. Obesity is considered as a state of chronic low-grade inflammation [132]. Although several in-vitro and animal experimental studies have suggested anti-inflammatory effects of vitamin D in obesity, results from human intervention studies have not shown effects on inflammatory cytokines in response to vitamin D supplementation [133–135].

In our search for SRs reporting effects of vitamin D supplementation on changes in body weight, we identified four SRs [82,136–138] among which three provided summary data in terms of a formal MA including data from 32 RCTs (Table Da in S1 File). Overall, the MAs showed no effects of vitamin D supplementation on changes in body weight, fat mass (FM), percentage FM (%FM), or lean body mass. Only the MA by Chandler et al. [138] showed a beneficial effect on weight loss of supplementation with CaD as compared to placebo.

Table Db in S1 File shows characteristics of the 32 RCTs included in the MA and summary characteristics of the trials are shown in Table 1. The 32 RCTs have been published between 1987 and 2014. One of the trials included in the MA by Chandler et al. [138] reported effects on treatment with active vitamin D [139], whereas the other 31 trials reported effects of treatment with vitamin D2 (2 RCTs) or D3 (29 RCTs). The majority of the individual trials reported null-findings. Only two trials found beneficial effects of supplementation [139,140]. In the WHI trial, a secondary analysis on weight changes showed that women randomized to the CaD group had smaller average annual weight gains than women assigned to the placebo group [140]. In contrast, Maki et al. [141] reported a significantly increased body weight in response to eight weeks of treatment with a daily multivitamin and mineral supplement containing D3 1,200 IU D3/d compared with the same supplement without D3. Weight loss was a primary endpoint in five of the trials [142–146] and none of these showed beneficial effects of vitamin D intervention. Most of the studies assessed effects in both men and women (N = 16 trials). Number of subjects included was less than 1000 in all of the trials except for the WHI trial [140]. Eleven (34%) of the trials included participants with (signs of) obesity, none of them showing beneficial effects of supplementation.

Six (19%) of the studies had low 25OHD levels (<50 nmol/L) at baseline as a criterion for inclusion, but mean 25OHD levels were below 50 nmol/L in almost half of the studies (N = 15) and 25OHD levels increased by more than 50% in N = 15 (47%) of the trials in response to the intervention (Table 1). Only one of the MAs have addressed the importance of 25OHD levels, showing no effects of either 25OHD levels archived in response to supplementation nor change in 25OHD levels from baseline in meta-regression analyses on obesity measures [137].

*In summary*, an inverse association between body weight and 25OHD levels is well documented. While it is generally agreed that obesity results in low 25OHD levels, it is still disputed whether low 25OHD levels may contribute to weight gain. Although most of the individual RCTs included in the MAs were relatively small in terms of number of participants and of short duration, the studies did, nevertheless, investigate effects of vitamin D supplementation in populations in which mean 25OHD levels were below 50 nmol/L, as well as 25OHD levels increased by more than 50% in approximately half of the studies. Accordingly, trial data do not support a beneficial effect of vitamin D supplementation on weight loss.

### Birth weight

Vitamin D insufficiency is common in pregnant women [147–149], and a number of studies have investigated effects of vitamin D status on pregnancy outcomes. Results from three SRs on findings from observational studies, suggest a reduced birth weight in infants born by mothers with low 25OHD levels [18,150,151]. In a MA by Aghajafari et al. [150] a significantly lower birth weight (weighted mean difference −131g; 95% CI, −187g to −75g) was found in infants born by mothers with 25OHD levels below 37.5 nmol/L during pregnancy. The MA also showed a significant inverse association between low 25OHD levels and risk of small gestational age (SGA) infants (OR 1.85; 95% CI, 1.52 to 2.26), as well as risk of gestational diabetes, pre-eclampsia, and bacterial vaginosis [150]. Similar findings were reported in a SR by Wei et al. [151], in which a MA of observational data showed an increased risk of SGA infants (OR 1.52, 95% CI, 1.08 to 2.15) if maternal 25OHD levels were below 50 nmol/L during pregnancy. Moreover, in an umbrella review of SRs, Theodoratou et al. [18] reported concordant results from MA of observational studies and randomised trials, suggesting an increased birth weight with improved vitamin D status.

Several biological mechanisms responsible for potential effects of vitamin D on fetal development have been suggested, including an importance of vitamin D on placental development through effects on human chorionic gonadotropin expression and placental sex steroid synthesis [152]. Moreover, an increased expression of 1α-hydroxylase and VDR in early pregnancy trophoblast and decidua tissue has been reported, suggesting an importance of vitamin D in early foetal development [153].

In our search for SRs on effects of vitamin D supplementation on birth weight, we identified eight SR [21,113,154–158] among which six [21,154,155,157–159] reported MAs on pooled data from RCTs on effects of vitamin D supplements on weight of new-born infants (Table Ea in S1 File). Three of the MAs were Cochrane reviews which have been continuously updated [21,155,159]. In the first of the published MA, Mahomed et al. [21] found a reduced birth weight in response to vitamin D supplementation in a daily dose of 1000 IU/d. However, this was based on data from two studies with a total of only 176 pregnant women. In one of the studies, the reported SD of mean birth weight was very low (reported to be 70–90g) whereas it normally is approximately 600 g [160]. It is likely that the standard error was reported instead of SD and this may have affected the pooled estimate of the MA. Accordingly, the authors of the MA conclude that too few data were available to draw conclusions on effects of supplementation on birth weight [21]. In the other five MAs, two showed an increased birth weight in response to vitamin D supplementation [158,159], whereas three reported null findings [154,155,157] (Table Ea in S1 File). In one of the SR, data were stratified by dose of vitamin D supplementation [157], showing no effects on birth weight in neither studies using high doses nor studies using low doses (Table Ea in S1 File).

The six MAs included data from 14 RCTs. Table Eb in S1 File shows characteristics of the individual trials included in the MAs and characteristics of the individual trials are summarized in Table 1.

Overall, four of the individual trials reported beneficial effects of the supplementation, whereas 10 RCTs reported null-findings. Birth weight/fetal growth was considered as a (co-)primary endpoint in three of the four studies reporting beneficial effects of supplementation [161–163]. The number of pregnant women included in the 14 trials varied from 50 to 350. In most studies, vitamin D supplementation was initiated in the 3^rd^ trimester, as only a few studies started the supplementation in the 2^nd^ trimester [163–166].

Supplementation with vitamin D3 was used in most trials (N = 9), and in three of the four trials showing beneficial effects on birth weight. All four studies showing a beneficial effect used high dose treatment in the 3^rd^ trimester either as a single high dose or high doses given 2–3 times. All of the four studies were performed as open label trials in non-western contries (three in India and one in Iran).

None of the studies had vitamin D insufficiency (25OHD < 50 nmol/L) as criteria for inclusion, and eight of the trials did not report 25OHD levels at baseline or changes in response to supplementation. None of the four studies showing beneficial effects reported changes in 25OHD levels in response to treatment and only one of the four studies reported baseline 25OHD levels. None of the published MAs, reported effects according to baseline 25OHD levels or changes in 25OHD levels.

Only the studies by Brooke et al. [167] and Hollis et al. [164] reported a 50% increase in 25OHD levels in response to treatment. The study by Brooke et al. [167] had, however, a rather small sample size whereas the study by Hollis et al. [164] had a reasonable sample size and used a relatively high vitamin D dose although including women who did not have vitamin D insufficiency (Table Eb in S1 File).

In *summary*, in contrast to findings from observational studies, results from most RCTs have not shown beneficial effect of vitamin D supplementation on birth weight. The quality of the trials showing an increased birth weight in response to supplementation is limited by the lack of blinding and measurement of 25OHD levels. Accordingly, available data from RCTs on effects of vitamin D supplementation on birth weight do not clearly support findings from observational studies. Of notice, the sample size in most trials have been relatively small and in most of the studies, the intervention has been of short duration and initiated in late pregnancy, whereas several observational studies showing an inverse association between 25OHD levels and birth weight have assessed vitamin D status in early/mid pregnancy [168]. Accordingly, the hypothesis raised by findings from observational studies on effects of vitamin D status on birth weight has not been properly tested in RCTs.

### Malignant diseases

A potential antineoplastic effect of vitamin D has been suggested by findings from *in vitro* studies, as vitamin D may affect several cellular processes, including proliferation, differentiation, angiogenesis, and apoptosis [169,170]. Although an association between vitamin D and risk of cancer has not been found in all human studies, several observational studies have reported an inverse association between risk of cancer and a low dietary intake of vitamin D, a low degree of sun exposure, or low plasma levels of 25OHD [171–173]. Studies on VDR polymorphisms (BsmI, TaqI, FokI, and ApaI) [174,175], and MAs on data from observational studies also support an association between low 25OHD levels and an increased risk of malignant diseases [176–180] as well as cancer mortality [181].

We identified eight SRs reporting data from RCT on risk of incident malignant diseases in response to vitamin D supplementation [52,54,113,182–186]. However, a formal MA reporting summary risk estimates was only performed in five of the SRs [52,54,184–186] (Table Fa in S1 File).

Following the Evidence/Technology Assessment report on health outcomes of vitamin D and calcium published in 2009 by Chung et al. [182], Chung and colleagues [183] published an update in 2011 on cancer outcomes. The SR reports data from three RCTs [55,187,188], but no pooled summary estimates are reported. The SR concludes that the available data do not allow for any firm conclusions on harm or benefits of vitamin D supplementation for cancer prevention. Moreover, in a SR on non-skeletal effects of vitamin D by Rosen et al [113], commissioned by The Endocrine Society, data from three RCTs are reviewed. The three studies included are similar to those reviewed by Chung et al [183]. No formal MA was performed. In accordance with the conclusion by Chung et al [183], Rosen et al [113] conclude that there is not sufficient evidence to draw conclusions on whether vitamin D may affect cancer incidence or mortality from cancer. However, the paper did not consider a prior analysis performed by Bolland et al. [189] showing an interaction between use of personal CaD supplements and randomization to CaD vs. placebo in the WHI trial. The analysis showed a significantly decreased risk of breast cancers and a non-significantly reduced risk of colorectal cancer in women randomized to CaD supplementation who did not take personal CaD supplements at randomization [189].

In a SR commissioned by the U.S. Preventive Services Task Force (USPSTF), aiming at updating the evidence on benefits and harms of vitamin and mineral supplementation to prevent cancer, Fortmann et al. [52] and Keum et al. [186] performed MAs on data from four RCTs on primary prevention of cancers in the general adult population. None of the analyses showed effects of vitamin D supplementation on risk of incident cancers, and this was not changed by stratification on whether the supplementation was provided as vitamin D alone or vitamin D in combination with calcium (Table Fa in S1 File).

Bjelakovic et al [184] published an elaborated Cochrane review including data from 18 RCTs on risk of incident cancers and cancer mortality. In the SR, a number of MAs were performed showing no significant effects of vitamin D supplementation on risk of developing cancer. However, based on data from four studies, the MA showed a significantly reduced risk on cancer mortality (RR 0.88; 95% CI, 0.78, 0.98). Similarly, a reduced risk of death due to malignancies (RR 0.88; 95% CI, 0.79–0.98, p = 0.03, I^2^ 0%) was also been reported in MAs by Zheng et al. [190] and Keum et al. [186], both pooling findings from three trials [55,188,191] with a total of 44,260 randomized participants.

In another SR, Bolland and colleagues [54] identified SRs published since 2009 that summarized data from RCTs on effects of vitamin D supplementation on risk of cancer. Three MAs were identified including seven RCT [182,183,192]. Bolland et al. [54] performed a new MA, based on data from the seven RCTs included in theses three SRs. The new MA showed no effects of vitamin D supplementation, either alone or in combination with calcium, on risk of incident cancers (Table Fa in S1 File).

In addition, Sperati et al [185] performed a SR focusing specifically on effects of vitamin D on risk of breast cancer. The authors identified only two studies fulfilling their pre-defined criteria for inclusion of studies (Table Fa in S1 File). The MA showed no beneficial effects of vitamin D supplementation and neither was vitamin D dosage nor mode of administration found to affect risk of breast cancer [185].

Table Fb in S1 File shows characteristics of the 19 RCT included in the five MA and Table 1 summarizes characteristics of the individual studies.

None of the RCTs assessed risk of cancer as a primary outcome. The majority of the studies was performed in women-only (58%) with less than 1000 randomized participants (68%) and no RCT included in published SRs provides data on effects of vitamin D supplementation in patients already diagnosed with cancer. Mean levels of 25OHD at baseline were below 50 nmol/L in less than half of the studies and only one study had low 25OHD levels as an inclusion criteria. Only one of the MAs addressed whether vitamin D status at baseline is of importance to responses, showing no difference in risk of incident cancers between trials with mean baseline levels below 50 nmol/L (RR 0.99; 95% CI, 0.93–1.05) compared with studies with mean 25OHD levels above 50 nmol/L (RR 1.12; 95% CI, 0.94, 1.34) [184].

The majority of studies investigated effects of vitamin D3 administered as a daily supplement. The MA by Bjelakovic et al [184], also included data from three studies on treatment with activated vitamin D analogues. More than half of the studies investigated effects of vitamin D alone, whereas calcium was co-administered in seven studies to either only the vitamin D group (5 RCT) or to both the vitamin D and the control group (3 RCT). Most of the studies (79%) had a duration of at least one year. The intervention with vitamin D supplementation increased plasma 25OHD levels by more than 50% in only one-third of the studies. Only one of the 19 RCTs showed a reduced incidence of cancers in response to supplementation with vitamin D. This study was performed by Lappe et al [187] and did not have malignant diseases as a primary outcome (Table Fb in S1 File). Most recently, Lappe et al [193] have published results from a subsequent double-blind RCT comparing effect of four-years of treatment with a daily supplement of D3 2000 IU plus 1500 mg of calcium with placebo on risk of incident all-type cancer (primary endpoint). The trial included 2,303 healthy elderly women with a mean age of 65±7 years and a mean baseline 25OHD level of 82±26 nmol/L. The study showed no significant beneficial effects on cancer incidence (HR 0.70; 95% CI, 0.47 to 1.02). However, the study probably had a too low statistical power to show such an effect [194].

*Conclusions*: MAs on results from RCTs on effects of vitamin D supplementation on risk of cancer have concluded that no evidence exists to support a causal relationship. It should, however, be emphasized that most RCTs included in the SRs have been carried out in groups of participants without low 25OHD levels and mean changes in 25OHD levels in response to supplementation have either not been assessed or been less than 50% of baseline values in most studies. In addition, the duration of the intervention has in most studies been rather short. Interestingly, the WHI study [189] as well as the trials by Lappe et al [187,187] had a relatively long duration and did suggest beneficial effects. More so, data on specific types on malignant diseases are largely missing. Accordingly, the evidence-base by which the MAs are grounded does not seem to be appropriate to reject the findings from observational studies on an increased risk of cancer in vitamin D insufficiency.

### Respiratory tract infections

For more than a century, vitamin D deficiency has been suggested to increase the susceptibility to infection. Early observations showed increased risk of respiratory tract infections (RTI) in children with nutritional rickets and vitamin D was considered of importance in the treatment of tuberculosis [195,196]. The seasonality of RTIs such as those caused by rhinovirus (“common cold”) and influenza virus is well known, and low 25OHD levels during wintertime have been suggested to be the “seasonal stimulus” which increases the susceptibility to such infections [197]. If so, this is of major importance to public health, as RTI are a major contributor to mortality [198,199]. Recent studies have provided further evidence of vitamin D as an important regulator of human immune function, as vitamin D may stimulate the innate immune response which provides front-line protection against infectious agents [200]. The VDR has been shown to be expressed in different cells of the myeloid and lymphoid lineage and vitamin D may increase the expression of antimicrobial peptides in human monocytes and neutrophils [201,202]. Particularly, vitamin D may enhance the expression of the human cathelicidin antimicrobial peptide (hCAP-18) which is of specific importance in host defenses against respiratory tract pathogens [203]. In accordance with the findings from epidemiological and *in-vitro* studies, a number of observational studies have suggested an inverse association between vitamin D status and risk of infections. Thus, risk of a recent RTI was significantly increased by 24% among participants with low compared with high 25OHD levels (below/above 75 nmol/L) in the Third National Health and Nutrition Examination Survey in the USA, which included 18,883 study subjects [204]. Similarly, an inverse association between vitamin D status and risk of developing RTIs has also been reported in other cohorts as well as a replete vitamin D status has been associated with fewer days of absence from duty due to RTIs [205–209].

In our search for SRs on effects of vitamin D supplementation on risk of RTIs, we identified 10 SR [22,210–218] among which seven [22,211–213,216–218] reported MAs on pooled data from RCTs on risk of RTIs in response to vitamin D supplementation (Table Ga in S1 File). In two trial-level MAs, vitamin D supplementation was found to significantly reduce risk of RTI by approximately 40% [211,212], which also applied to pediatric populations [211]. The first of the trial MA found a protective effect by summarizing data from five RCTs [211], the second trial MA included data from 11 RCTs [212]. A subsequent trial-level MA by Mao et al [213], however, found no beneficial effects of vitamin D supplementation on risk of RTI. Mao et al [213] included only data from seven RCTs, as this MA excluded studies which were considered to be of low quality in terms of a modified Jadad score ≤ 3 [219]. Most recently, an individual patient data analysis (IPD) has been published, including individual data from 10,933 study subjects included in 25 RCTs [22]. The analysis showed a significantly reduced risk of acute RTI (OR 0.88, 95% CI, 0.81 to 0.96). Sub-group analyses suggested protective effects in response to a daily or weekly vitamin D dose (adjusted odds ratio 0.81, 0.72 to 0.91), but not in response to one or more bolus doses (adjusted OR 0.97, 95% CI, 0.86 to 1.10; P_i_ = 0.05). Moreover, among those receiving daily or weekly vitamin D, the protective effects were stronger in those with a baseline 25OHD <25 nmol/L (adjusted OR 0.30, 95% CI, 0.17 to 0.53) than in those with a baseline 25OHD ≥25 nmol/L (adjusted OR 0.75, 95% CI, 0.60 to 0.95; P_i_ = 0.006) [22]. However, two of the other published MAs found no differences between studies with mean baseline levels above or below 75 nmol/L [212] or 50 nmol/L [218].

The seven MAs included data from a total of 30 RCTs. Table Gb in S1 File shows characteristics of the individual RCTs included in the seven MAs and characteristics of the individual trials are summarized in Table 1. None of the MAs included data from all the 30 RCTs.

The majority of studies included both men and women. Only two studies were of a large scale with more than 1000 participants [220,221] and most studies had a relatively short duration. Twenty-three (77%) of the RCTs investigated effects of vitamin D supplementation as a primary endpoint (Table Gb in S1 File). Eleven trials (37%) included patients diagnosed with an infection at time of inclusion or at increased risk of acquiring infections. However, the specific infections studied varied widely between studies e.g., some investigated risk of pneumonia, upper or lower RTI, or exacerbations in patients with COPD or asthma as well as some studies included new-borns or infants where other studies focused on adults or elderly (Table Gb in S1 File). All studies investigated effects of supplementation with vitamin D3 and half of the trials (53%) administered D3 as a daily dose without concomitant calcium supplementation. Only one of the trials had low 25OHD levels (< 50 nmol/L) as inclusion criteria and mean 25OHD levels at baseline were only reported in two-thirds of the studies among which only seven trials reported mean levels below 50 nmol/L. Nine trials (30%) reported a more than 50% increase in 25OHD levels in response to treatment. A beneficial effect of vitamin D supplementation on risk of infections was found in nine (30%) of the trials, but none of the above mentioned indices were in general a characteristic of the trials showing beneficial effects of the supplementation (Table Gb in S1 File).

In *summary*, most published studies on effects of vitamin D supplementation on risk of RTI have been relatively small and of short duration without specifically addressing effects in populations with vitamin D insufficiency. Beneficial effects of vitamin D supplementation have been reported in nine of the 30 RCTs included in the 7 published MAs and three of the MAs have concluded that vitamin D supplementation may lower risk of infections. Noteworthy, the populations studied in the individual trials have varied widely from newborns to elderly as well as effects of a wide range of different types of infections have been included raising the question whether results from such different settings can be merged into MA reporting summary estimates. Nevertheless, the overall findings suggest a beneficial effect of vitamin D on respiratory tract infections.

### Depression

Vitamin D may affect brain function as the VDR as well as the 1α-hydroxylase enzyme is expressed by brain tissues, suggesting that 25OHD is activated and functions within the central nervous system (CNS) [222–224]. This is further supported by findings showing effects of 1,25-OH_2_D3 on the synthesis of nerve growth factor (NGF) [225,226], as well as the synthesis of tryptophan is transcriptionally activated by vitamin D and low 25OHD levels may accordingly cause low levels of serotonin [227].

Several cross-sectional- and cohort-studies have reported an inverse association between plasma 25OHD concentrations and depression [228–234]. In a MA on results from observational studies, low plasma 25OHD levels were associated with an increased risk of depression in cross-sectional studies (n = 11 studies with 43,137 participants; OR 0.96; 95% CI, 0.94, 0.99, I^2^ = 63%) as well as in cohort studies (n = 5 studies with 12,648 participants; OR 0.92; 95% CI, 0.87, 0.98, I^2^ = 50%) [235]. Nevertheless, other epidemiological studies have not found such associations [236–238].

We identified six SRs [239–244] among which four [239–242] provided formal MAs reporting pooled data on results from 30 RCTs on effects of vitamin D supplementation on depression (Table Ha in S1 File).

Performing a systematic literature search, Spedding et al. [239] identified 15 RCTs reporting effects of vitamin D supplementation on depression scores including mood disorders such as (seasonal) recurrent depressive disorder. Studies were subsequently grouped according to the presence of biological flaws. Biological flaws were considered if baseline 25OHD levels were not measured, if baseline levels indicated a replete vitamin D status, or if the interventions did not cause a significant increase in 25OHD levels. Among the 15 studies identified, eight (53%) were found to have biological flaws [61,245–251], whereas seven studies (47%) were classified as being without flaws [252–258]. MAs were performed on studies with or without biological flaws. In the MA on studies without biological flaws, only two of the seven studies were included [253,257], as they used the same outcome measure in terms of Beck Depression Inventory (BDI), whereas other measures were used in the remaining five studies. Among the eight studies grouped as being with biological flaws, only two were included in MA [61,251] as they both used the 12-item Short Form Health Survey (SF-12) questionnaire, whereas other measures were used in the remaining six studies. As shown in Table Ha S1 File, the authors found beneficial effects of vitamin D supplementation in the MA on studies without biological flaws, whereas no effect was present in the MA on studies classified as being with biological flaws.

In a systematic review by Li et al. [240] studies were only included if vitamin D was provided orally as mono-intervention and compared with placebo in adults considered to be at risk of depression, having depressive symptoms, or being diagnosed with depression (secondary or tertiary prevention). The protocol for the study was published prior to the results from the MA [259]. The MA included data from six RCTs (Table Hb in S1 File) among which one of the studies used calcitriol as treatment [250]. For some of the included studies, only sub-groups of the randomized population were considered for data-analyses. For example, in the study by Sanders et al. [61], only 137 of the 2258 randomized women were included, as only this subgroup provided concurrent information on changes in 25OHD levels and changes in scores as assessed by the WHI-5 questionnaire. Overall, the meta-analysis by Li et al. [240] showed no effects of vitamin D supplementation on studied indices (Table Ha in S1 File). In subgroup analysis stratified by vitamin D dosages, sex, study location, and different study populations, no effects of vitamin D were found. In a post hoc analysis stratified by vitamin D status at baseline (sufficient vs deficient), no significant differences between groups were observed [240]. Similarly, Shaffer et al [241] and Gowda et al [242] found no significant differences when comparing studies with mean baseline 25OHD levels below or above 50 nmol/L. For most of the analyses performed, a substantial unexplained heterogeneity was present and the quality of evidence obtained from the included trials was graded as low by the authors [240]. Similarly, no overall beneficial effects of vitamin D supplementation were found in the MAs by Shaffer et al (221) and Gowda et al [242] and a substantial and unexplained heterogeneity was also found in most analyses (Table Ha in S1 File).

Table Hb in S1 File shows summary characteristics of the 12 RCTs included in the four MAs. The trials have been published between 2003 and 2013. In four (33%) of the trials, depression was considered as a primary end-point. Four of the RCTs included only women (none of the studies included only men). Three studies included solely patients with a diagnosis of depression; none of them on treatment with antidepressant at time of inclusion. One study included only elderly women with known seasonal affective disorders (treatment status unknown). The remaining studies included community-dwelling men and women among which two of the studies excluded participants on treatment with antidepressants. In three studies, participants were only included if they had 25OHD levels below 40 or 55 nmol/L or between 25–75 nmol/L. In 10 of the studies, vitamin D supplementation was given orally as vitamin D3 tablets/capsules (9 studies) or as fortified cheese (1 study). One study used intramuscular D3-injections and one study tested calcitriol. Vitamin D was given daily (7 studies), weekly (3 studies), yearly (1 study), or as a single dose (1 study). Time from initiation of treatment to end of follow-up varied from six weeks to five years. Effects were assessed by Beck Depression Inventory (BDI) in five studies, by the Geriatric Depression Scale (GDS) in two studies, and by the SF-12 questionnaire in two studies. One study used the Burnam 8-item scale for depressive disorders whereas another study used a questionnaire designed for patients with fibromyalgia which included depression as a domain. Only three of the individual RCTs showed beneficial effects [254,257,260] among which effects of the intervention was considered as a primary endpoint in two of the trials [257,260]. In one study, a per-protocol analysis showed a decrease in depressive symptoms compared with placebo in the cognitive-affective domain (subscale 1–13) of BDI [253]. In a sub-analysis from the WHI study, the Burnam-score was worsened by vitamin D compared with placebo (0.009; 95% CI, 0.002, 0.017) in the group of women without evidence of prior depression [246]. No major effects were shown in sub-analyses in any of the other studies.

*In summary*, only one of four MAs has suggested a beneficial effect of vitamin D supplementation on depression. This MA included only few of the published studies, whereas the three MAs showing no effects of vitamin D supplementation allowed for inclusion of more of the published RCTs. However, the methods used to assess effects in the various RCTs varied widely and only few (N = 5) studies reported an increase in 25OHD levels above 50% in response to the intervention. Moreover, most of the studies (N = 11) did not require their participants to have vitamin D insufficiency (25OHD<50 nmol/L) at inclusion and only five studies reported mean baseline 25OHD levels below 50 nmol/L. It is nevertheless noteworthy that both the MA by Li et al. [240] and the MA by Shaffer et al [241] suggested beneficial effects of vitamin D supplementation among those with a replete vitamin D status, but not in subjects with low vitamin D levels which does not support findings from observational studies of a major role of vitamin D in the pathogenesis of depression.

### Mortality

Observational studies have shown an inverse association between 25OHD levels and mortality, including death from CVD, cancer, and non-vascular, non-cancer causes [194,261–266]. The mechanism by which supplementation with vitamin D may reduce mortality has not been fully clarified, but it has been suggested that the reduced risk of death may be due to the possible pleiotropic effect that involved the musculo-skeletal- as well as several extraskeletal-systems, including anti-inflammatory and immune modulating effects [267,268].

We identified 12 SRs with MAs on mortality in response to supplementation with vitamin D. Eight of the MAs showed a significantly reduced mortality in response to supplementation (Table Ia in S1 File). In most of the MAs, stratification by type of calciferol showed a significantly reduced mortality in response to treatment with vitamin D3, whereas no beneficial effects were found in response to treatment with vitamin D2 [190,263,269–271]. Discrepant results have been reported on whether mortality is reduced in response to treatment with vitamin D alone [263,269] or only if vitamin D is combined with calcium supplements [190,270–272]. In two MAs, effects of the intervention was significant in summary estimates on trials with low baseline vitamin D status [190,271], but a clear dose-response relationship has not been documented [49,269,271,272].

The 12 MAs included data from a total of 59 RCTs. Table Ib in S1 File shows characteristics of the individual RCTs included in the 12 MAs and characteristics of the individual trials are summarized in Table 1. None of the RCTs had mortality as a primary endpoint and none of them showed a significantly reduced mortality in response to treatment. Actually, two small trials showed an increased mortality in response to the supplementation [273,274]. In the study by Latham et al. [273], a single high dose (300,000 IU) of vitamin D3 increased mortality during the following six months compared with placebo in a group of 243 frail elderly people. Similarly, a high dose of D2 (100,000 IU every 3^rd^ month) increased mortality compared with no treatment during a 10 months study period in a group of 3,717 nursing home residents [274]. On the other hand, none of the studies with daily administration of vitamin D supplements has shown increased mortality in response to supplementation, including a recently published trial on three-years of daily supplementation with 4000 IU D3 or placebo to 400 patients with heart failure [275]. Most studies have been relatively small with less than 1000 randomized participants and have investigated effects of a daily dose of vitamin D3 with calcium being co-administered in approximately half of the studies. Only few of the studies (N = 5) had low 25OHD levels as a criterion for inclusion. Seventeen of the 59 studies reported an increase in 25OHD levels of more than 50% in response to the intervention. Mean 25OHD levels were below 50 nmol/L in approximately half of the studies. Several of the MAs compared effects between studies with mean baseline levels below or above 50 nmol/L showing no significant interactions [49,270,276,277]. However, stratified analyses showed a significantly decreased mortality when pooling results from studies with mean 25OHD levels below 50 nmol/L, but not when combining results from studies with mean levels above 50 nmol/L [190,270,276].

*In summary*, supplementation with vitamin D seems to be causally related to a reduce all-cause mortality, which is in accordance with the findings from observational studies. Moreover, as reviewed in the section on malignant diseases, MAs on cause specific mortality have shown a reduced mortality due to malignant diseases [184,186,190]. Only vitamin D3 has been shown to reduce mortality, and some MAs have suggested that the effect only is present if calcium is co-administered, whereas other MAs have shown an effect of vitamin D3 alone. The lack of a clear dose-response relationship may be attributable to characteristics of the RCTs included in the MAs, as most of the trials have investigated effects of low doses (Table Ib in S1 File) which may limit the statistical power to show dose-effect relationships. Furthermore, as recently shown in a simulation analysis, based on findings from a large cohort study, the expected impact on mortality of vitamin D supplementation is presumably much higher if targeted to individuals with low 25OHD levels [278].

## Discussion

Overall, 16 of the 54 MAs suggested beneficial effects of vitamin D supplementation on different extra-skeletal outcomes among which eight of 12 MAs (66%) reported a decreased mortality. Beneficial effects were also suggested by MAs on blood pressure (2 of 9 [22%] MAs), birth weight (2 of 6 [33%] MAs), RTIs (3 of 7 [43%] MAs), and depression (1 of 4 [25%] MAs). No beneficial effects were reported in any of the MAs on CVD, T2D, body weight, or malignant diseases.

For all studied outcomes, only one-fourth of the individual RCTs included in the MAs had assessed effects studied as a primary endpoint. None of the trials included in the 12 MAs on mortality had mortality as a designated primary outcome. Most studies have been performed only in women or in a mixed gender population, whereas only very few studies included only men. The sex distribution is probably because many of the included studies have investigated skeletal effects as primary outcomes. As osteoporosis and fractures are more common in women than in men, these studies have preferentially included women in order to achieve effects in a population at high risk. However, although many of the trials have assessed effects of vitamin D supplementation on skeletal outcomes, only a minority of the studies have actually investigated effects in populations with low 25OHD levels. Plasma 25OHD levels below 50 nmol/L were only inclusion criteria in one-fourth of the analyses.

Moreover, mean 25OHD levels at baseline below 50 nmol/L has only been measured in less than half of the studies, indicating that the populations studied did not, on average, suffer from vitamin D insufficiency and the duration of studies has in many studies been relatively short. The WHI study is the trial with the longest time of intervention (7 years) and the most participants included [279].

The vast majority of observational studies have reported low 25OHD levels to be associated with an increased risk of adverse health outcomes, but caution is needed in interpreting observational data. Although most studies have adjusted for differences between groups, residual confounding can never be excluded in observational studies. Effects may be due to lifestyle factors, a “healthy user” bias or other differences between groups, which has not been fully accounted for in adjusted analyses. This may result in reverse causation, in which vitamin D status is more a marker for an underlying condition/disease than causally related to the disease investigated. Accordingly, RCTs are needed to determine whether a causal link is present. Following the publications of a large number of observational studies showing adverse health outcomes of low 25OHD levels, the requests of data from RCTs have increased. For each of the outcomes we studied, a variable number of RCTs have been published and most of them have failed to document a beneficial effect as shown in observational studies. Accordingly, it may seem questionable whether the findings from observational studies actually reflect a beneficial effect of vitamin D by itself. Due to such findings, a recent large scale review by Autier et al [19] concluded that the inverse association between 25OHD levels and various health outcomes, could be the result of disease process causing low 25OHD levels rather than low 25OHD levels causing the disease i.e., low 25OHD levels are a marker of ill health.

In our review, we aimed to summarize characteristics of the published RCTs on effects of vitamin D supplementation. Although, RCTs are considered as the highest level of evidence, and especially if results from RCTs are summarized in MA, the degree of confidence on the findings from the RCTs do, however, depend on how the studies were performed and whether they actually had a design and statistical power to detect possible effects. Our review highlights that most of the available trials were not designed to study non-skeletal outcomes of vitamin D supplementation. For most of the selected outcomes reviewed in this paper, the RCTs included in MAs did not have the disease in question as primary outcome. For several of the diseases studied, some of the outcomes were assessed as adverse event reports and many of the studies had a questionable statistical power to detect effects such as incident CVDs or cancers. The lack of trials assessing effects of intervention on specific health outcomes as a primary endpoint does, however, not provide a complete answer to the discrepancy between findings from observational and randomized trials. For example, in analyses on mortality, none of the individual RCTs showed beneficial effects and none of the trials were designed to assess mortality. However, several of the pooled risk estimates in MAs did show a significantly reduced risk of death in response to treatment. Notably, the MAs on mortality have included substantially more studies than most of the MAs on other studied outcomes and mortality may be assessed in a more valid manner than other “adverse effects” observed during a trial. If vitamin D supplementation affects studied outcomes the sample size may have been too small to show effects, which may have been further hampered by a relatively short duration of treatment. CVD, obesity, T2D, and cancers develop over a long time, and the duration of the trials available may not be sufficiently long to affect such disease processes. Historically, it has been shown multiple times that RCTs are difficult to perform in terms of testing the right dose for an appropriate time-period in a suitable population of study subjects and that null-finings do not necessary disprove important effects [17,280,281]. Interestingly, a beneficial effect of vitamin D supplementation was shown in several of the trials on RTIs, which are outcomes that seems more likely to be affected by a relatively short duration of treatment compared with for example CVD or cancer. The most noticeable difference between the observational and randomized trials is, however, that only few of the RCTs included participants with vitamin D insufficiency. Many studies did not report mean 25OHD levels, but among studies reporting these values, mean levels were below 50 nmol/L in less than half of the studies and the intervention did not result in a marked increase in 25OHD levels in several of the trials. Only a few of the included MAs investigated whether findings differed according to baseline vitamin D status showing no significant interaction between results from studies with mean 25OHD levels below vs. above 50 nmol/L or according to whether supplementation resulted in increased 25OHD levels on cardiovascular outcomes [49], blood pressure [81], body weight [137], malignant diseases [184], RTI [212,218] or mortality [49,270,276,277]. A significant interaction term (P_i_ = 0.006) was only shown for RTI in the recently published MA by Martineau et al [22] showing a larger decrease in risk of RTI if baseline 25OHD levels are below as compared with above 25 nmol/L. Of importance, a mean level below 50 nmol/L implies that only approximately half of the population had levels below 50 nmol/L, which may have blunted a potential effect in subjects with low levels. Accordingly, it is questionable whether available trials as well as MAs performed by combining trial results in reality have tested the hypothesis raised by observational studies on adverse health effects of low 25OHD levels. A beneficial effect of vitamin D supplementation is most likely difficult to demonstrate if studies are performed in populations with adequate levels. Moreover, factors such as compliance, sources of vitamin D, etc. have to be considered when evaluating findings from RCTs as well as in the design of further studies [194,282]. We choose to classify studies according to a cut-off plasma level of 25OHD equal to 50 nmol/L, as consensus exist on considering this threshold limit as a state of inadequacy [283], although some have suggested that a sufficient vitamin D status requires levels above 80 nmol/L [284]. Importantly, the optimal 25OHD level for non-skeletal health outcomes have not yet been clarified and the available RCTs do not allow for such definitions [27]. Lack of effects may be attributable to use of too low doses of vitamin D to archice a sufficient response. In terms of this, it may be of relevance in future studies also to consider the relationship between vitamin D dose, baseline and achieved 25OHD concentrations as well as the fact that the relationship between vitamin D dose and 25OHD concentration is nonlinear and can vary due to personal factors [194].

A further limitation to some of the RCTs might be that some of the studies have used intermitten treatment with high doses of vitamin D. Although such dosing regimes results in high levels of 25OHD, levels of cholecalciferol are neglible for most of the time due to the rapid 25-hydroxylation of vitamin D3 into 25OHD. Vitamin D3 acts as a substrate in many tissues thereby being of importance to the vitamin D endocrine/autocrine system. Accordingly, it is likely that vitamin D3 should be available on a daily basis through supplementation or endogenous synthesis in order to archive effects of vitamin D3 [285]. It may also be questioned whether effects of supplementation with vitamin D are equal to endogenous synthesis. In recent studies, novel secosteroids with potential actions similar to 25OHD have been shown to be synthesized in vivo in human epidermis and in pig adrenal glands [286]. These compounds may function as substrates for the 1α-hydroxylase and thereby potentially act in a manner similarly to activated vitamin D. Further studies are needed to determine whether this is of importance to the effects of vitamin D and if it makes a difference whether vitamin D is synthesized endogenously or provided as in terms of supplementation with cholecalciferol. Additionally, UV irradiation through sun-exposure may also cause liberation of nitric oxide from subcutaneous nitrogen stores thereby affecting non-skeletal outcomes such as blood pressure beyond potential effects of vitamin D synthesis [287–289]. Finally, it should also be noted that our assessement of findings on non-skeletal outcomes is not exhaustive, as effects of vitamin D supplementation also have been studied on other outcomes such as dental caries [290].

### Directions for further investigations

As highlighted by our review, the quality of data from available RCTs is of limited value in terms of assessing non-skeletal effects of vitamin D supplementation. Further MAs performed on these data are therefore not to be considered as a priority. MA does not compensate for shortcomings in the original studies. Further RCTs designed to assess effects of vitamin D supplementation on specific outcomes within a population at risk are warranted. A number of large-scale trials are currently being performed. These studies aim at assessing non-skeletal outcomes of vitamin D supplementation and first results are expected within the next year [291,292]. Although these studies plan to include from 6,000 to 25,874 participants, and thereby potentially provide a substantial statistical power to detect potential effects, most of these studies are not targeted towards subjects with vitamin D insufficiency [293]. Accordingly, they do not assess effects of the intervention in a population at specific risk where potential effects of improving vitamin D status may be blunted by inclusion of individuals with a replete vitamin D status.

Assessing effects of vitamin D supplementation in randomized trials is complicated by a number of factors [294]. Even if a large-scale study was performed in a population with vitamin D insufficiency at baseline, changes in vitamin D status may occur in the control group during the course of the trial due to altered dietary habits and/or sun-exposure, including seasonal-variations [295–297]. Moreover, vitamin D supplements are readily available and controls may start using vitamin supplements. Finally, assessment of effects in diseases which develop over a long time span such as CVD or obesity may be difficult during the course of a trial which most often only runs for a few years. Effects may also depend on the age of studied subjects and risk for developing such chronic diseases may be grounded in early life [298,299]. If so, a replete vitamin D status throughout life or at certain ages may be of importance. Although RCTs are considered to provide the highest degree of evidence on causal relationships, this methodology may not be appropriate to rely on, if the effects of vitamin D are of importance at a certain period of life or during a long time span. Thus, the importance of well-designed longitudinal studies should not be entirely rejected. Development of new methods or reliance on the Bradford Hill criteria may be necessary to refind our understanding of vitamin D [300]. A major limitation to the interpretation of prior longitudinal studies is lack of standardization of 25OHD measurements [301]. It has been documented that use of different methods for measuring 25OHD levels may cause huge variations in levels measured and thereby affect the classification of vitamin D status [302–305]. Further observational studies should therefore aim at using standardized methods for vitamin D measurements in order to allow for comparisons across studies and time periods. Such observational studies may also help to provide guidance on threshold values of 25OHD levels of importance to non-skeletal outcomes.

### Conclusions

Observational studies have suggested an inverse association between vitamin D status and a number of diseases. Results from RCTs and MAs of RCTs do, however, only provide limited support for such effects, as most studies have failed to document significant effects. Nevertheless, several MAs have suggested a reduced mortality in response to vitamin D s supplementation, as well as three of seven MAs showed a beneficial effect on RTIs, including a recently published large individual patient data analysis [22]. Only sparse evidence are available for a beneficial effect of supplementation on other non-skeletal outcomes such as blood pressure, depression, and increased birth weight. No MAs based on findings from RCTs have so far provided support for a beneficial effect of supplementation on risk of CVD, T2D, body weight, or malignant diseases. More than half of the available trials have been performed in individuals without vitamin D insufficiency and the intervention has not resulted in an adequate increase in 25OHD levels in many of the trials. Furthermore, the sample size in most RCTs was relatively small and the trials had a short duration of follow-up. In accordance with the null findings in most RCTs, most meta-analyses have not shown effects of vitamin D supplementation on non-skeletal outcoms. Importantly, as evaluated by the AMSTAR score, the quality of published MAs has largely been fair to good. The null findings of MAs are therefore not attributable to an inappropriate methodology when combining data from RCTs. MAs cannot be expected to proof potential effects of supplementation with vitamin D, if the trials forming the basis for the MAs have not been performed in populations with low 25OHD levels. Overall, a definite causal link between vitamin D insufficiency and non-skeletal diseases has not been proven, but the RCTs performed so far cannot be considered to have tested the hypothesis raised by observational studies on adverse health outcomes of low vitamin D levels. As a “healthy user” bias and reverse causation cannot be excluded in observational studies, further RCTs designed to test the hypothesis of a beneficial effect of optimizing vitamin D status in individuals with an impaired vitamin D status are warranted, as well as long-term cohort studies using standardized methods for serial measurement of 25OHD levels.

## Supporting information

S1 File Supplementary tables. (DOCX)

S2 File PRISMA checklist. (DOC)
