# Supplementary material for: Non-skeletal health effects of vitamin D supplementation: A systematic review on findings from meta-analyses summarizing trial data
Source: PLoS One. 2017 Jul 7;12(7):e0180512. doi: 10.1371/journal.pone.0180512 (PMC5501555; doi:10.1371/journal.pone.0180512)
Supplement: S1 File — (DOCX) [file pone.0180512.s001.docx]

S1 File

**Appendix: Non-skeletal health effects of vitamin D supplementation: a systematic review on findings from meta-analyses summarizing trial data**

**Content of appendix**

[Abbreviations 3](#_Toc481325007)

[Supplementary Tables 5](#_Toc481325008)

[Table 2A. Systematic reviews on risk of cardiovascular diseases 5](#_Toc481325009)

[Table 2B. RCTs on risk of cardiovascular diseases. 8](#_Toc481325010)

[Table 3A. Systematic reviews on effects of vitamin D on blood pressure 13](#_Toc481325011)

[Table 3B. RCTs on effects of vitamin D supplementation on blood pressure. 16](#_Toc481325012)

[Table 4A. Systematic reviews on risk of type 2 diabetes 33](#_Toc481325013)

[Table 4B. RCTs on risk of type 2 diabetes. 34](#_Toc481325014)

[Table 5A. Systematic reviews on body weight 35](#_Toc481325015)

[Table 5B. RCTs on body weight 36](#_Toc481325016)

[Table 6A. Systematic reviews on birth weight 43](#_Toc481325017)

[Table 6B. RCTs on birth weight. 45](#_Toc481325018)

[Table 7A. Systematic reviews on risk of malignant diseases 48](#_Toc481325019)

[Table 7B. RCTs on risk of malignant diseases 50](#_Toc481325020)

[Table 8A. Systematic reviews on respiratory tract infections 55](#_Toc481325021)

[Table 8B. RCTs on risk of respiratory tract infections. 58](#_Toc481325022)

[Table 9A. Systematic reviews on depression. 67](#_Toc481325023)

[Table 9B. RCTs on risk of depression. 68](#_Toc481325024)

[Table 10A. Systematic reviews on mortality 72](#_Toc481325025)

[Table 10B. RCTs on mortality 76](#_Toc481325026)

[Search string 90](#_Toc481325027)

[PRISMA Checklist 91](#_Toc481325028)

[Flow Chart (PRISMA) showing identification of included systematic reviews 94](#_Toc481325029)

[References for the appendix 95](#_Toc481325030)

# Abbreviations

B-25OHD: Plasma levels of 25OHD at baseline

BDI: Beck Depression Inventory

BMI: Body mass index

BP: Blood pressure

Ca: Calcium

CaD: Calcium + vitamin D supplementation

CHF: Chronic heart failure

CKD: Chronic kidney disease.

CNS: Central nervous system

CV: cardiovascular

CVD: Cardiovascular disease

D2: Ergocalciferol

D3: Cholecalciferol

DB: Double blind study

DBP: diastolic blood pressure

EOS: End of study

FIQ: Fibromyalgia Impact Questionnaire

FM: Fat mass

GDS: Geriatric Depression Scale

GHQ: General Health Questionnaire

HA: Arterial hypertension

HADS: The Hospital Anxiety and Depression Scale

HbA1c: Hemoglobin A1c

HR: Hazard ratio

HRT: Hormone replacement therapy

IC-25OHD: low 25OHD levels required for study inclusion (Yes/No)

IFG: Impaired fasting glucose

IM: intramuscular injection

IQR: Interquartile range

ITT: intention to treat

IU: international unit

MA: meta-analysis

MADRS: The Montgomery–Åsberg Depression Rating Scale

MCS: mental component score

MI: myocardial infarction

NB: None blinded (open label) study

NFG: Normal fasting glucose

NR: Not reported

P_i_: P-value for interaction analysis

RTI: Respiratory tract infections

SB: single blind study

SBP: systolic blood pressure

SR: systematic review

SCID-CV: The Structured Clinical Interview for DSM-IV Axis I Disorders – Clinician Version

SF-12: 12-item Short Form Health Survey

SMD: standard mean difference

SPAQ: Seasonal Pattern Assessment Scale

SR: Systematic review

T2D: Type 2 diabetes

URI: Upper respiratory tract infections

VitD: Vitamin D

WHI: Womens health initiative

WHO-5: The WHO 5-Well-Being Index

Δ 25OHD >50%: Whether the intervention increased plasma 25OHD levels >50% (Yes/No)

#

# Supplementary Tables

Please note, Table 1 is shown in the main paper.

## Table Aa. Systematic reviews on risk of cardiovascular diseases

SR with MA reporting summary data on findings from RCT on effects of vitamin D supplementation on risk of cardiovascular diseases (CVD).

| **Systematic reviews on effects of vitamin D supplementation on risk of cardiovascular diseases** | | | | |
| --- | --- | --- | --- | --- |
| **MA#** | **Reference, publication year** | **Papers included** | **Criteria for inclusion of studies** | **Results** |
| CV1 | Wang, 2010 [1] | 4 RCT:  VitD-alone: (2 RCT with N=2,988 participants [2,3].  CaD: 2 RCT with N=36,474 participants [4,5]. | Systematic search identifying RCT reporting effects of treatment with VitD-alone or CaD on risk of CV events in adults. | No effects on CVD:  VitD- alone: RR 0.90 (95% CI, 0.77 to 1.05)  CaD: RR 1.04 (95% CI, 0.92 to 1.18)  I^2^ not reported |
| CV2 | Elamin, 2011 [6] | 7 RCT:  MI (6 RCT, N=39,879 participants):  [2–4,7–9]  Stroke (6 RCT, N=39,739 participants): [2–4,8–10] | RCT that enrolled adults who received VitD supplementation and a concurrent comparison group that did not receive VitD.  Studies were included regardless of their language, size, or duration of follow-up.  Excluded studies with activated VitD analogues | No effects on CVD:  MI: RR 1.02 (95% CI, 0.93 to 1.13; I^2^ = 0%)  Stroke: RR 1.05 (95% CI, 0.88 to 1.25; I^2^ = 15%) |
| CV3 | Myung, 2013 [11] | 7 RCT with N=43,443 participants  [2–5,7,9,12] | RCT reporting efficacy of VitD supplements for the prevention of CVD.  At least 6 mo. of follow-up | No effects on CVD:  Major CV events:  VitD alone (N=2 RCT): RR 0.95 (95% CI, 0.86 to 1.05, I^2^=5%)  VitD combined with others (N=5 RC): RR 1.04 (95% CI, 0.99 to 1.10, I^2^=0%)  VitD alone or combined (N=7 RCT): RR 1.02 (95% CI, 0.98 to 1.07, I^2^=23%)  CV death (N=3 RCT): RR 0.90 (95% CI, 0.76 to 1.07, I^2^=27%)  MI (N=2 RCT): RR 1.06 (95% CI, 0.92 to 1.21, I^2^=0%)  Stroke (N=5 RCT): RR 1.00 (95% CI, 0.88 to 1.13, I^2^=6%) |
| CV4 | Mao, 2013 [13] | 6 RCT with N=45,933 participants  [2–5,14,15] | The MA included RCT published between 1980 – 2012, with a minimum of 12 months of follow-up, and in which intervention with VitD was compared with placebo. | No effects on CVD:  VitD alone:  Major CVD events: OR 0.94 (95% CI, 0.81 to 1.08, I^2^=0%)  MI: OR 0.86 (95% CI, 0.54 to 1.37, I^2^=0%)  Stroke: OR 1.09 (95% CI, 0.87 to 1.37, I^2^=0%)  CaD:  Major CVD events: OR, 1.06 (95% CI, 0.94 to 1.19; I^2^=0%)  MI: OR 1.06 (95% CI, 0.92 to 1.21, I^2^=0%)  Stroke: OR 0.98 (95% CI, 0.86 to 1.13; I^2^=0%) |
| CV5 | Fortmann, 2013 [16] | *VitD-alone:* 2 RCT with N=7,978 participants [2,17].  *CaD:* 1 RCT (WHI) with N=36,282 participants [18]. | Included only RCT considered as having fair- and good-quality assessing effects of supplements in the *primary prevention* of CVD in *the general adult population* (community-dwelling, nutrient-sufficient adults) without a history of CVD.  Excluded supplement doses higher than the upper tolerable limit set by the U.S. Food and Nutrition Board | No effects on CVD:  VitD alone: RR 0.94 (95% CI, 0.87 to 1.02)  CaD: RR 1.01 (95% CI, 0.95 to 1.07)  I^2^ not reported |
| CV6 | Ford, 2014 [17] | MI: 11 RCT with N= 11,717 participants  [2,3,14,19–24]  [25,26].  Stroke: 11 RCT with N=11,841 participants  [2,3,14,21,22,24,26–29]. | Included only RCTs that studied participants aged >60 y and with at least 1 yr. of follow-up.  Treatment with any VitD or VitD analogue was allowed.  Co-administration with other medications, such as calcium, was allowed provided that the comparator group received the same medication  Excluded studies solely assessing effects in patients with kidney diseases, steroid induced osteoporosis and psoriasis. | No effect on CVD  MI: RR 0.96 (95% CI, 0.83 to 1.10)  Stroke: RR 1.07 (95% CI, 0.91 to 1.29)  Risk estimates represents pooled estimates form studies on vitamin D2/D3 as well as from studies on effects of activated vitamin D analogs [19–21,28]  . |
| CV7 | Bolland, 2014 [30] | MI: 9 RCT with N=48,647 participants [2–4,7,10,14,15,18,24]  Stroke: 8 RCT with N=46,431 participants [2–4,7,8,10,14,15]. | Through a systematic search on PubMed, the authors identified MA, published since January, 2009, reporting data from RCT on effects of VitD supplementation on risk of CVD. Four MA were identified [1,6,31,32] reporting data from 7 RCT. A new MA was performed on data from the 7 RCT.  Studies were excluded if they were cluster randomized or investigated effects of hydroxylated VitD or VitD analogues. The analyses also excluded trials that included other interventions only in the VitD group, trials of fortified dairy products, and trials in populations with chronic comorbidity other than osteoporosis or frailty | No effects on CVD:  *Myocardial infarction or ischemic heart disease*:  VitD alone: RR 0.99 (95% CI, 0.86 to 1.13; I^2^=0%)  CaD: RR 1.18 (95% CI, 0.86 to 1.63; I^2^=31%)  VitD with or without Ca: RR 1.02 (95% CI, 0.93 to 1.13; I^2^=0%)  *Stroke or cerebrovascular disease*:  VitD alone: RR 1.09 (95% CI, 0.92 to 1.30; I^2^=0%)  CaD: RR 0.99 (95% CI, 0.87 to 1.13; I^2^=0%)  VitD with or without Ca: RR 1.01 (95% CI, 0.90 to 1.13; I^2^=0%) |

## Table Ab. RCTs on risk of cardiovascular diseases.

Characteristics of randomized clinical trials included in MAs (Table 2A) on effects of vitamin D supplementation on risk of cardiovascular diseases (CVD).

| **Characteristics of RCTs included in MAs on effects of vitamin D supplementation on risk of cardiovascular diseases** | | | | | | | | | | |
| --- | --- | --- | --- | --- | --- | --- | --- | --- | --- | --- |
| **Reference, publication year, country** | **Included in MA** | | | | | | | **Population**  No. of randomized (% females), Characteristics of study | **Intervention** | **Results** |
|  | CV1 | CV2 | CV3 | CV4 | CV5 | CV6 | CV7 |  |  |  |
| Inkovaara, 1983 [10], Finland |  | X |  |  |  |  | X | N=87 (82% F) living at elderly care homes, aged > 65 yr.  Mean age ≈79±6 yr.  IC-25OHD <50 nmol/L: No  B-25OHD: NR  Δ 25OHD > 50%: NR | 9 mo. of treatment (DB):  D3 1000 IU/d  Vs.  Placebo | CVD was not primary outcome.  Stroke: RR 2.49 (95% CI, 0.71 to 8.76) |
| Aloia, 1988 [19], USA |  |  |  |  |  | X |  | N=27 postmenopausal women with osteoporosis, aged 50–80 y  IC-25OHD <50 nmol/L: No  B-25OHD: ≈58±20 nmol/L  Δ 25OHD > 50%: Yes | 2 yr. of treatment (DB):  0.50 μg calcitriol/d  Vs.  Placebo  All received D3 400 IU/d and had a calcium intake of at least1000 mg/d | CVD was not primary outcome.  MI: RR 1.00 (95% CI, 0.07 to 14.72) by comparing calcitriol with placebo, as reported by [17] |
| Ott, 1989 [20], USA |  |  |  |  |  | X |  | N=86 postmenopausal women with vertebral compression fractures.  IC-25OHD <50 nmol/L: No  B-25OHD: 66 nmol/L  Δ 25OHD > 50%: NR | 2 yr. of treatment (DB)  Calcitriol, mean dose 0.43 µg/d.  Vs.  Placebo  Dose and dietary calcium were adjusted for hypercalciuria or hypercalcemia. | CVD was not primary outcome.  MI: RR 0.33 (95% CI, 0.01 to 7.96) by comparing calcitriol with placebo, as reported by [17] |
| Mark, 1996 [12], China |  |  | X |  |  |  |  | N=3,318 (56% F) diagnosed with esophageal dysplasia, aged 40-69 yr., from rural China  IC-25OHD <50 nmol/L: No  B-25OHD: NR  Δ 25OHD > 50%: NR | 6 yr. of treatment (DB):  Multiple vitamin/mineral supplement containing vitD 800 IU/d + Ca 324 mg/d  Vs.  Placebo | CVD was not primary outcome.  Cerebrovascular mortality: RR 0.63 (95% CI, 0.37 to 1.07) |
| Komulainen, 1999 [7], Finland |  | X | X |  |  |  | X | N= 464 non-osteoporotic early postmenopausal women aged 47-56-yr.  Median age: 53 yr.  IC-25OHD <50 nmol/L: No  B-25OHD: NR  Δ 25OHD > 50%: NR | 5 yr. of treatment:  HRT  Vs.  D3 (300 IU/d → 100 IU/d during the fifth year)  Vs.  3) HRT + D3 combined  Vs.  Placebo  Women randomized to D3 and placebo also received Ca 93 mg/d | CVD was not primary outcome.  CVD reported as adverse events: No significant effects on risk of MI or stroke. |
| Gallagher, 2001/2004 [21,33], USA |  |  |  |  |  | X |  | N=246 women with normal age-adjusted bone mineral density, aged 65–77 yr.  IC-25OHD <50 nmol/L: No  B-25OHD: ≈80±27 nmol/L  Δ 25OHD > 50%: No | 3 yr. of treatment (DB):  Calcitriol 0.5 μg/d  Vs.  Placebo | CVD was not primary outcome.  MI: RR 1.49 (95% CI, 0.54 to 4.13) by comparing calcitriol with placebo, as reported by [17]  Stroke: RR 1.42 (95% CI, 0.55 to 3.68) by comparing calcitriol with placebo, as reported by [17] |
| Trivedi, 2003 [2], UK | X | X | X | X | X | X | X | N=2,686 (24% F) community-dwelling aged 65­85 yr.  IC-25OHD <50 nmol/L: No  B-25OHD: ≈ NR  Δ 25OHD > 50%: NR | 5 yr. of treatment (DB):  D3 100 000 IU/ 4^th^ mo.  Vs  Placebo / 4^th^ mo., | CVD was not primary outcome.  Total CVD: RR 0.90 (95% CI, 0.77 to 1.06)  CV deaths: RR 0.84 (95% CI, 0.65 to 1.10) |
| Avenell, 2004 [27], UK |  |  |  |  |  | X |  | N=134 (84% F) patients with a previous low-trauma fracture, aged > 70 yr.  Mean age ≈77±6 yr.  IC-25OHD <50 nmol/L: No  B-25OHD: NR  Δ 25OHD > 50%: NR | 1 yr. of treatment (DB):  D3 800 IU/d  Vs.  Ca 1000 mg/d  Vs.  D3 800 IU/d + Ca 1000 mg/d  Vs.  Placebo. | CVD was not primary outcome.  Stroke: RR 0.31 (95% CI, 0.01 to 7.36) as reported by [17] |
| Matsumoto, 2005 [28], Japan |  |  |  |  |  | X |  | N=219 (98% F) osteoporotic patients aged 49–87 yr.  Mean age ≈68±7 yr.  IC-25OHD <50 nmol/L: No  B-25OHD: ≈43±15 nmol/L  Δ 25OHD > 50%: NR | 1 yr. of treatment (DB) withED-71 (an activated vitD analog):  ED-71 0.5 μg/d  Vs.  ED-71 0.75 μg/d  Vs.  ED-71 1.0 μg/d  Vs.  Placebo  All received D3 in a dose of 400 IU/d if 25OHD<50 nmol/L and 200 IU/d if 25OHD ≥ 50 nmol/L | CVD was not primary outcome.  Stroke: RR 0.32 (95% CI, 0.02 to 5.02) by comparing ED-71 with placebo, as reported by [17] |
| Brazier, 2005 [4], France | X | X | X | X |  |  | X | N=192 community-dwelling ambulatory women aged >65 years with low 25OHD levels  Mean age 75±7 yr.  IC-25OHD <50 nmol/L: Yes  B-25OHD: 18 nmol/L  Δ 25OHD > 50%: No | 1 yr. of treatment (DB):  D3 800 IU + Ca 1000 mg/d  Vs  Placebo | CVD was not primary outcome.  CVD are reported as adverse events  No effects on CVD risk - similar number of CV events in both groups: (N=6 vs. N=6) |
| RECORD study as reported by Grant, 2005 [14] and Ford, 2014 [17], UK |  |  |  | X | X | X | X | N=5,292 (85% women) aged > 70 yr. with a previous low-trauma fracture.  IC-25OHD <50 nmol/L: No  B-25OHD: 38±16 nmol/L (subgroup)  Δ 25OHD > 50%: Yes (subgroup following one year of treatment) | Mean 45 (range 24-62) mo. of treatment (DB):  D3 800 IU/d  Vs.  Ca 1000 mg/d  Vs.  D3 800 IU/d + Ca 1000 mg/d  Vs.  Placebo. | CVD was not primary outcome.  No effects on CVD events : RR 0.93 (95% CI, 0.81 to 1.07) as reported by Fortmann et al [16] |
| WHI study (USA) as reported by Jackson, 2006 [8], Wactawski-Wende, 2006 [18], and Hsia, 2007 [5] | X | X | X | X | X |  | X | N=36,282 post-menopausal women  IC-25OHD <50 nmol/L: No  B-25OHD: NR  Δ 25OHD > 50%: NR | 7 yr. of treatment (DB):  D3 400 IU + Ca 1000 mg/d  Vs.  Placebo | CVD was not primary outcome.  Major cardiovascular events: RR 1.05 (95% CI, 0.92 to 1.19)  MI: RR 1.05 (95% CI, 0.91 to 1.21)  Stroke: RR 0.96 (95% CI, 0.83 to 1.11)  CVD death (RR 1.01). |
| Lappe, 2007 [15], USA |  |  |  | X |  |  | X | N=1,179 healthy community-dwelling women aged > 55 yr.  IC-25OHD <50 nmol/L: No  B-25OHD: ≈72±20 nmol/L  Δ 25OHD > 50%: No | 4 yr. of treatment (DB):  D3 1100 IU + Ca 1500 mg/d  Vs.  Ca-only: 1500 mg/d  Vs  Placebo | CVD was not primary outcome.  No effects on risk of CVD, according to analyses by [6,13], risk estimates are:  Major cardiovascular events: OR 0.64 (95% CI, 0.24 to 1.72)  MI: OR 0.64 (95% CI, 0.09 to 4.60)  Stroke: OR 0.80 (95% CI, 0.21 to 3.02) |
| Berggren, 2008 [9], Sweden |  | X | X |  |  |  |  | N= 199 (74% F) patients operated on for femoral neck fracture, aged ≥70 years  Mean age 87 yr.  IC-25OHD <50 nmol/L: No  B-25OHD: NR  Δ 25OHD > 50%: NR | 1 yr. of treatment:  D3 800 IU/d + Ca 1000 mg/d  Vs.  None  A multidisciplinary, multifactorial fall-prevention program was applied in the intervention group. | CVD was not primary outcome.  No effects on risk of CVD. According to analyses by Elamin et al. [6], risk estimates are:  MI: RR 1.12 (95% CI, 0.81 to 1.53)  Stroke: RR 1.71 (95% CI, 0.97 to 3.02)  The intervention group had a high prescription of calcium and vitamin D (66%) than the control group (22%) |
| Prince, 2008 [3], AU | X | X | X | X |  | X | X | 302 community-dwelling ambulant women with a history of falling in the past 12 months, aged 70-90 yr.  Mean age ≈77±5 yr.  IC-25OHD <50 nmol/L: No  B-25OHD: ≈45±12 nmol/L  Δ 25OHD > 50%: No | 12 mo. of treatment (DB):  D2 1000 IU + Ca 1000 mg/d  Vs.  Placebo + Ca 1000 mg/d | CVD was not primary outcome.  Ischemic heart diseases: RR 0.67 (95% CI, 0.11 to 3.93)  Stroke: RR 1.00 (95% CI, 0.21 to 4.88) |
| Zhu, 2008 [22], AU |  |  |  |  |  | X |  | 120 community-dwelling women aged 70–80 yr.  Mean age 74.8±2.6 yr.  IC-25OHD <50 nmol/L: No  B-25OHD 68±29 nmol/L  Δ 25OHD > 50%: Yes | 5 yr. of treatment (DB):  D2 1000 IU + Ca 1200 mg/d  Vs.  Placebo-VitD + placebo-Ca | CVD was not primary outcome.  MI: RR 3.08 (95% CI, 0.13 to 74.42) as reported by [17]  Stroke: RR 0.51 (95% CI, 0.05 to 5.43) as reported by [17] |
| Xia, 2009 [23], China |  |  |  |  |  | X |  | 150 Chinese women with osteopenia/osteoporosis aged > 65 yr.  Mean age ≈70±4 yr.  IC-25OHD <50 nmol/L: No  B-25OHD: NR  Δ 25OHD > 50%: NA | 12 mo. of treatment (not-DB):  0.25 μg calcitriol + Ca 600 mg/d + D3 125 IU/d  Vs.  Ca 600 mg/d + D3 125 IU/d | CVD was not primary outcome.  MI: RR 3.08 (95% CI, 0.13 to 74.42) as reported by [17] (comparing calcitriol with no calcitriol) |
| Sanders, 2011 [24], AU |  |  |  |  |  | X | X | N=2,258 community-dwelling women aged > 70 yr. and at risk of fracture  Median age 76 yr.  IC-25OHD <50 nmol/L: No  B-25OHD 49 nmol/L (sub-study)  Δ 25OHD > 50%: No (sub-study) | 3–5 yr. of treatment (DB):  D3 500,000 IU/yr.  Vs.  Placebo | CVD was not primary outcome.  No effect of the intervention on risk of CVD. According to analyses by Ford et al. [17], risk estimates are:  Stroke: RR 1.33 (95% CI, 0.46 to 3.82)  MI: RR 1.49 (95% CI, 0.25 to 8.93) |
| Lehouck, 2012 [25], Belgium |  |  |  |  |  | X |  | 182 patients (20% F) with moderate to very severe COPD and a history of recent exacerbations  Mean age: 68±8 yr.  IC-25OHD <50 nmol/L: No  B-25OHD: 50±28 nmol/L  Δ 25OHD > 50%: Yes | 1 year of treatment (DB):  D3 100 000 IU/month  Vs.  VitD-Placebo/month | CVD was not primary outcome.  MI: RR 0.33 (95% CI, 0.04 to 3.15) as reported by [17] |
| Gallagher, 2012 [29], USA |  |  |  |  |  | X |  | 163 healthy postmenopausal white women aged 57–90 yr.  Mean age: 68±8 yr.  IC-25OHD < 50 nmol/L: Yes  B-25OHD: 39±10 nmol/L  Δ 25OHD > 50%: Yes | 1 year of treatment (DB):  Placebo  Vs  D3 in different doses:  400, 800, 1600, 2400, 3200, 4000, and 4800 IU/d.  All received daily calcium to maintain intake of 1200–1400 mg | CVD was not primary outcome.  Stroke: RR 0.46 (95% CI, 0.02 to 10.98) as reported by [17] |
| Witham, 2013 [26], UK |  |  |  |  |  | X |  | N=159 (48% F) with isolated systolic hypertension aged >70 yr.  Mean age 77±4.6 yr.  IC-25OHD <50 nmol/L: No  B-25OHD: 45±15 nmol/L  Δ 25OHD > 50%: Yes | 1 year of treatment (DB):  D3 100,000 IU/every 3^rd^ mo.  Vs  Placebo/every 3^rd^ mo. | CVD was not primary outcome.  MI: RR 0.20 (95% CI, 0.01 to 4.05) as reported by [17]  Stroke: RR 2.96 (95% CI, 0.31 to 27.88) as reported by [17] |

## Table Ba. Systematic reviews on effects of vitamin D on blood pressure

SR with MA reporting summary data on findings from RCTs on effects of vitamin D supplementation on blood pressure.

| **SR on effects of vitamin D supplementation on blood pressure.** | | | | |
| --- | --- | --- | --- | --- |
| **MA#** | **Reference, publication year** | **Papers included** | **Criteria for inclusion of studies** | **Results** |
| BP1 | Witham, 2009 [34] | 4 RCT with N= 389 participants  [35–38]. | Only RCT comparing VitD with placebo – co-treatment with Ca in both arms was permitted - trials on CaD vs. placebo were excluded  Included UVB study [36]. | VitD compared with controls (WMD):  **SBP: -6.18 (95% CI, -12.32 to -0.04) mmHg (I^2^ = 74%)**  DBP: -2.56 (95% CI, -5.83 to 0.72) mmHg (I^2^ = 67%) |
| BP2 | Wu, 2010 [39] | 4 RCT with N= 429 participants  [35,37,40,41]. | Only double-blind RCT of oral VitD supplementation in normotensive or hypertensive individuals with BP measurements were included.  Excluded patients with diseases such as diabetes and CKD | All studies used D3.  VitD compared with calcium or placebo (WMD):  **SBP: -2.44 (**95% CI, **-4.86 to -0.02) mmHg**  DBP: -0.02 (95% CI, -2.19 to 1.94) mmHg  Subgroup analysis – no effects of:  - dose of vitD (< vs. *≥* 400 IU/d)  - study length (< vs. *≥ 10* wk)  *VitD alone vs. CaD (P_i_>0.99):*  VitD alone (3 RCT, N=366): SBP -2.39 (95% CI, -5.70 to 0.92); DBP -0.03 (95% CI, -1.98 to 1.92)  CaD (1 RCT): SBP -2.50 (95% CI, -6.06 to 1.06); DBP 0.00 (95% CI, -2.67 to 2.67) |
| BP3 | Pittas, 2010 [42] | 10 RCT with N=37,162 participants  [35–38,41,43–47]. | Systematic search identifying RCT reporting effects of treatment with VitD with or without calcium on BP in adults.  Included only studies performed in generally healthy populations (<20% of study participants had major chronic diseases, such as diabetes, cancer, or CVD, at baseline).  Included UVB study by [36] | In MA of 10 trials (WMD):  SBP: -1.9 (95% CI, -4.2 to 0.4) mm Hg (I^2^ = 69%)  DBP:-0.1 (95% CI, -0.7 to 0.5) mm Hg (I^2^ = 23%)  Sensitivity: results not changed by exclusion of WHI  *VitD vs. CaD:*  Change in SBP and DBP did not differ between trials that tested vitD alone and those testing CaD (i.e. no effects)  *Dose response - dose < vs. ≥1000 IU/d:*  SBP: No effect of dose  DBP: larger reduction in response to ≥1000 IU/d (−1.5 mm Hg) than  <1000 IU/d (+0.1 mm Hg; P=0.04) |
| BP4 | Elamin, 2011 [6] | 14 RCT with N=1518 participants  The MA does not report from which trials data were extracted to compute the summary effect sizes | RCT that enrolled adults who received VitD supplementation and a concurrent comparison group that did not receive VitD.  Studies were included regardless of their language, size, or duration of follow-up.  Excluded studies with activated VitD analogs | VitD compared with controls (WMD):  SBP: -0.06 (95% CI, 1.98 to 1.87) mmHg (I^2^ = 61%)  DBP: -0.34 (95% CI, -1.03 to 0.35) mmHg (I^2^ = 0%) |
| BP5 | Kunutsor, 2014 [48] | 16 RCTs with N=1,879 participants | Only RCTs aiming to study the effects of oral vitD supplementation alone.  Excluded studies in which the intervention was calcitriol or one of its analogues and those with participants not receiving an intervention to raise 25OHD levels.  Primary outcome was the difference in office or ambulatory SBP and DBP among treatment and control groups. | No overall effect in trial level meta-analyses.  Sub-analysis showed a significant reduction in DBP (95 % CI --2.28 to -0.34 mmHg, P = 0.01) in participants with pre-existing cardiometabolic disease. |
| BP6 | Beveridge, 2015 [49] | 38 RCTs (N=4058 participants) for trial level MA.  IPD: 27 RCTs (3092 participants) – includes active vitD trials. | - RCTs with vitD for a minimum of 4 weeks  - Co-interventions were permitted if identical in all treatment arms | No overall effect in trial level or IPD analyses.  D3 trials: no significant relationship was found on meta-regression  between SBP or DBP treatment effect and the daily dose equivalent used as treatment, baseline 25OHD-levels, baseline PTH-levels or trial duration |
| BP7 | Manousopoulou, 2015 [50] | 5 RCTs with N=819 participants [45,46,51–53]. | Only RCTs including adults with obesity  - Interventions in addition to VitD (e.g. calcium co-administration, exercise program, weight loss regime) applied equally to both groups. | VitD significantly **increased** SBP (WMD: 0.237; 95% CI, 0.110 to 0.365).  No effect on DBP |
| BP8 | Golzarand, 2016 [54] | 30 RCTs with N=4744 participants | RCTs on effect of D3 on SBP and DBP | No effect on SBP (−0.68 mmHg, 95%CI: −2.19 to 0.84) or DBP (−0.57 mmHg, 95%CI: −1.36 to 0.22).  **Sub-group analyses:**  - Daily vitamin D3 at a dose of >800 IU/day for <6 months in subjects ≥50 years reduced both SBP and DBP (p < 0.001).  - Hypertensive effect in overweight and obese subjects  ……After excluding overweight and obese subjects, VitD3  significantly reduced SBP and DBP  CaD: significantly increased SBP (3.64 mmHg, 95%CI: 3.15 to 4.13) and DBP (1.71 mmHg, 95%CI: 1.25 to 2.18).  Meta-regression analysis: no association between dose of vitamin D and changes in SBP and DBP |
| BP9 | Lee KJ , 2016 [55] | SBP: 15 RCTs with 1,134 patients  DBP: 13 RCTs with N=793 patients. | Only T2D patients: RCTs on effects of VitD supplementation on BP.  Included only studies in which the efficacy of vitamin D on BP was expressed as mean with and standard deviation. | No effect on SBP (SMD –0.121 (95% CI –0.355 to 0.113) mmHg; I^2^ = 74%).  Significantly (p=0.02) lower DBP (SMD –0.160 (95% CI –0.298 to –0.022) mmHg, I^2^ = 0%).  In meta-regression analyses, study duration and vitamin D dose did not influence effects of vitamin D on blood pressure  Analyses included data from one study on paricalcitol [56] |

## Table Bb. RCTs on effects of vitamin D supplementation on blood pressure.

Characteristics of randomized clinical trials included in MA (Table 3A) on effects of vitamin D supplementation on blood pressure (BP).

| **Characteristics of RCTs included in MAs on effects of vitamin D supplementation on blood pressure** | | | | | | | | | | | |
| --- | --- | --- | --- | --- | --- | --- | --- | --- | --- | --- | --- |
| **Reference, publication year, country** | **Included in MA *** | | | | | | |  | **Population**  No. of randomized (% females), Characteristics of study | **Intervention** | **Results** |
|  | **BP1** | **BP2** | **BP3** | **BP5** | **BP6** | **BP7** | **BP8** | **BP9** |  |  |  |
| Pan, 1993 [40], Taiwan |  | X |  |  |  |  |  |  | N= 58 institutionalized elderly persons (27% F)  63.8 % of the participants were taken antihypertensive drugs.  Mean age: 74.2 ± 4.4 yr.  IC-25OHD $<$ 50 nmol/L: No  B-25OHD: 63 nmol/L  Δ 25OHD > 50%: No | For 11 weeks treatment (DB) with :  Ca 800 mg/d  Vs  D3 200 IU/d  Vs.  D3 200 IU/d + Ca 800 mg/d  Vs  Placebo | Primary outcome: hypertension  No effect on SBP or DBP  WMD SBP: -0.23 mmHg  WMD DBP: 1.20 mmHg |
| Scragg, 1995 [35], UK | X | X | X | X | X |  |  |  | N=189 (54 % F) recruited from the general population, aged 63-76  All had vitamin D deficiency  Excluded if using antihypertensive drugs,  Mean age 70 yr.  IC-25OHD $<$ 50 nmol/L: No  B-25OHD 33 nmol/L  Δ 25OHD > 50%: No | 5 weeks of follow-up after a single oral dose (DB):  D3 100 000 IU  Vs.  Placebo | Primary outcome: Blood pressure  No effect on SBP or DBP:  WMD DBP 0.00 mmHg (95% Cl, -2.57 to 2.57)  WMD SBP 0.00 mmHg (95% Cl -2.15 to 2.15) |
| Krause, 1998 [36], USA | X |  | X |  |  |  |  |  | N=18 (34% F) patients with untreated mild essential HA, aged 26-66 yr.  Mean age: 48 yr.  IC-25OHD $<$ 50 nmol/L: No  B-25OHD: 48 nmol/L  Δ 25OHD > 50%: Yes | 6 weeks of full-body exposures to (NB):  UVB irradiation thrice-weekly  Vs.  UVA irradiation thrice-weekly | Primary outcome: blood pressure  24-h ambulatory BP  UVB:  WMD SBP: -6 mmHg (95% CI, –14.94 to 2.94)  **WMD DBP: -8 mmHg (95% CI, –13.32 to –2.68) mmHg, P < 0.001**  UVA: no effect on BP |
| Pfeifer, 2001 [37], Germany | X | X | X | x | X |  | X |  | N=148 women, 39% had a history of hypertension and 46% were on treatment with CVD drugs  Age 74 ± 1 yr.  IC-25OHD $<$50 nmol/L: Yes  B-25OHD: 25 nmol/L  Δ 25OHD > 50%: Yes | 8 weeks of treatment (DB):  D3 800 IU/d + Ca 1200 mg/d  Vs.  Ca 1200 mg/d | Primary outcome: blood pressure  D3 significantly decreased SBP  **WMD SBP -6.50 mmHg (95%Cl -12.36 to -0.64)**  WMD DBP -0.30 mmHg (95% Cl -3.46 to 2.86) |
| Schleithoff, 2006 [43], Germany |  |  | X | x |  |  | X |  | N=123 (17% F) with CHF, almost everyone on BP medication  Mean age ≈ 55 yr  IC-25OHD <50 nmol/L: No  B-25OHD: 38 nmol/L  Δ 25OHD > 50%: Yes | 9 mo. of treatment (DB):  D3 2000 IU/d + Ca 500 mg/d  Vs.  Placebo + Ca 500 mg/d | BP was a secondary endpoint  No effect on office SBP or DBP |
| Major, 2007 [41], CA |  | X | X |  |  |  |  |  | N=63 healthy obese/overweight normotensive (BP <160/95mm Hg) premenopausal women with a Ca intake < 800 mg/d  Mean age 44±5 yr.  IC-25OHD <50 nmol/L: No  B-25OHD: NR  Δ 25OHD > 50%: NR | 15 weeks of treatment (DB):  D3 400 IU/d + Ca 1200 mg/d  Vs.  Placebo  Both groups observed a 700 kcal/d energy restriction | BP was a secondary endpoint  No effects on office SBP or DBP |
| Sugden, 2008 [38], Scotland | X |  | X | X | X |  |  | X | N=34 (47% F) with T2D. Most on BP medication  Age 64±10 yr.  IC-25OHD: < 50 nmol/L: Yes  B-25OHD: 38 nmol/L  Δ 25OHD > 50%: No | 8 weeks of follow-up after a single oral dose (DB):  D2 100 000 IU  Vs.  Placebo | Primary outcome: BP/endothelian function  D2 significantly decreased office SBP by 14 mm Hg compared with placebo  A non-significant fall in DBP (p=0.08)  WMD SBP: -13.90 mmHg (95% CI, –21.16 to -6.64)  WMD DBP: -4.50 mmHg (95% CI, –9.40 to 0.40) mmHg |
| Margolis, 2008 [44], WHI study, USA |  |  | X |  |  |  |  |  | N= 36,282 post-menopausal women. 28% with self-reported antihypertensive treatment.  Age 62±7 yr.  IC-25OHD <50 nmol/L: No  B-25OHD: NR  Δ 25OHD > 50%: NR | 7 years of treatment (DB):  D3 400 IU/d + Ca 1000 mg/d  Vs.  Placebo | Blood pressure was *not* primary outcome  No effects on office SBP or DBP  Sub-group analysis on women without hypertension at baseline (N=17,122): HR 1.01 (95% CI: 0.96 to 1.06.) |
| Nagpal, 2009 [45], India |  |  | X | X | X | X | X |  | N= 71 apparently healthy men with centrally obesity. Excluded men with hypertension.  Aged ≥ 35 yr.  IC-25OHD <50 nmol/L: No  B-25OHD: 33±13 nmol/L  Δ 25OHD > 50%: Yes | 3 oral doses administrated two weeks apart (DB) – with follow-up 2 weeks after last dose:  D3 120 000 IU  Vs.  Placebo | BP was a secondary endpoint  No effects on office SBP or DBP |
| Zittermann, 2009 [46], Germany |  |  | X |  | X | X | X |  | N=165 (67% F) healthy overweight subjects recruited from the background population. Excluded subjects with CVD.  Age: 48±10 yr.  IC-25OHD <50 nmol/L: No  B-25OHD: 30±19 nmol/L  Δ 25OHD > 50%: Yes | 12 mo. of treatment (DB):  D3 3320 IU/d  Vs.  Placebo  All participated also in a weight-reduction program. | BP was a secondary endpoint  No effect on office SBP or DBP |
| Jorde, 2009 [47], Norway |  |  | X |  |  |  |  | X | N=32 (44% F) with T2D for more than 1 yr. and on treatment with insulin and metformin, aged 21–75 yr. Excluded subjects with CVD.  Mean age ≈56±7 yr.  IC-25OHD <50 nmol/L: No  B-25OHD: 59±17 nmol/L  Δ 25OHD > 50%: Yes | 6 mo. of treatment (DB):  D3 40,000 IU/week  Vs.  Placebo | BP was a secondary endpoint  No effect on office SBP or DBP |
| Jorde, 2010 [53]; Norway |  |  |  | X | X | X | X |  | N= 438 (64% F) overweight or obese subjects without diabetes or IHD.  Mean aged 48±11 yr.  IC-25OHD <50 nmol/L: No  B-25OHD: 58±21 nmol/L  Δ 25OHD > 50%: Yes | 12 mo. Of treatment (DB)  D3 20,000 IU/week + 500mg Ca/d  Vs.  D3 40,000 IU/week + 500mg Ca/d  Vs  Placebo + 500 mg Ca/d | BP was a secondary endpoint  No effect on office SBP or DBP |
| Witham, 2010 [57], Scotland |  |  |  | X | X |  | X | X | N=61 (33% F) with T2D and 25OHD <100 nmol/L.  IC-25OHD <50 nmol/L: No  B-25OHD: 58±21 nmol/L  Δ 25OHD > 50%: Yes | 16 weeks of follow-up after a single oral dose (DB) of: D3 100,000 IU  Vs.  D3 200,000 IU  Vs.  Placebo | BP was a secondary endpoint  No effect on office SBP or DBP |
| Witham, 2010 [58], Scotland |  |  |  |  | X |  |  |  | N=105 (23% F) older adults with heart failure  Mean age 80±6 yr.  IC-25OHD $<$ 50 nmol/L: Yes  B-25OHD: 22±9 nmol/L  Δ 25OHD > 50%: Yes | 20 weeks of follow-up after two doses (10 weeks apart) of (DB):  D2 100,000 IU  Vs.  Placebo. | BP was a secondary endpoint  No effect on office SBP or DBP |
| Nikooyeh, 2011 [59], Iran |  |  |  |  |  |  |  | X | N=90 (61% F) patients with T2D aged 30–60 yr.  Mean age 51±7 yr.  IC-25OHD $<$ 50 nmol/L: No  B-25OHD: 42±45 nmol/L  Δ 25OHD > 50%: Yes | 12 weeks of daily intake (DB) of:  Plain yogurt drink (Ca 300 mg/d without D3)  Vs.  D3 Fortified yogurt drink (D3 1000 IU/d + Ca 300 mg/d)  Vs.  CaD fortified yogurt drink (D3 1000 IU/d + Ca 500 mg/d) | BP was a secondary endpoint  No effect on office SBP or DBP |
| Maki, 2011 [52], USA |  |  |  |  |  | X | X |  | N=60 (75% F) aged 18–79 yr. with high waist circumference.  Mean age 52±2 yr.  IC-25OHD $<$ 50 nmol/L: No  B-25OHD: 64±4 nmol/L  Δ 25OHD > 50%: No | 8 weeks of treatment (DB):  D3 1,200 IU/d + Daily multivitamin and mineral supplement  Vs  Daily multivitamin and mineral supplement without D3 | BP was a secondary endpoint  No effect on office SBP or DBP |
| Harris, 2011 [60], USA |  |  |  |  | X |  |  |  | N=45 (53% F) African-American adults with no overt cardiovascular, pulmonary or metabolic disease  Mean age 30±2 yr.  IC-25OHD $<$ 50 nmol/L: No  B-25OHD: 36±3 nmol/L  Δ 25OHD > 50%: Yes | 16 weeks of treatment (DB):  D3 60,000 IU monthly  Vs.  Placebo | BP was a secondary endpoint  No effect on office SBP or DBP |
| Shab-Bidar, 2011 [61], Iran |  |  |  | X | X |  |  | X | N=100% F) (47% F) with T2D, but not on insulin treatment  Mean age 52±7 yr.  IC-25OHD < 50 nmol/L: No  B-25OHD: 36±3 nmol/L  Δ 25OHD > 50%: Yes | 12 weeks of intervention with plain yogurt drink fortified with:  D3 1000 IU/d + 340 mg Ca/d  Vs.  No fortification | BP was a secondary endpoint  No effect on office SBP or DBP |
| Larsen, 2012 [62], Denmark |  |  |  | X |  |  | X |  | N=112 (69% F) hypertensive patients  Mean age 61±10 yr.  IC-25OHD < 50 nmol/L: No  B-25OHD: 58±25 nmol/L  Δ 25OHD > 50%: Yes | 20 weeks of therapy (DB):  D3 3000 IU/d  Vs.  Placebo | BP was primary endpoint (DB):  No effect on 24-h BP, although central systolic BP decreased significantly.  Post-hoc subgroup analysis of  92 subjects with baseline 25OHD < 80 nmol/L showed a significant decreases in 24-h SBP and DBP. |
| Alvarez, 2012 [63], USA |  |  |  |  | X |  |  |  | N=46 (5% F) with mild to moderate kidney disease.  Mean age 62±9 yr.  IC-25OHD < 50 nmol/L: No  B-25OHD: 27±7 nmol/L  Δ 25OHD > 50%: Yes | 1 year of oral therapy (DB):  D3  50,000 IU/week for 12 weeks followed by 50,000 IU every other week  Vs.  Placebo | BP was a secondary endpoint  No effect on office SBP or DBP |
| Gepner, 2012 [64], USA |  |  |  | X | X |  | X |  | N=114 healthy community  dwelling post-menopausal women with 25OHD between 25-150 nmol/L  Mean age 64±3 yr.  IC-25OHD < 50 nmol/L: No  B-25OHD: 78±28 nmol/L  Δ 25OHD > 50%: Yes | 4 mo. of therapy (DB):  D3 2500 IU/d  Vs.  Placebo | BP was a secondary endpoint  No effect on office SBP or DBP |
| Ahmadi, 2013 [65], Iran |  |  |  |  |  |  |  | X | N=60 (63% F) patients with T2D and nephropathy.  Mean age 58±11 yr.  IC-25OHD < 50 nmol/L: No  B-25OHD: 38±18 nmol/L  Δ 25OHD > 50%: Yes | 12 weeks of therapy (DB):  D3 50,000 IU/wk.  Vs.  Placebo | BP was a secondary endpoint  No effect on office SBP or DBP |
| Longenecker, 2012 [66], USA |  |  |  |  |  |  | X |  | N= 45 (22% F) HIV-infected adults  Mean age 44±9 yr.  IC-25OHD < 50 nmol/L: Yes  B-25OHD: 23 nmol/L  Δ 25OHD > 50%: Yes | 12 weeks of therapy (DB):  D3 4000 IU/d  Vs.  Placebo | BP was a secondary endpoint  No effect on office SBP or DBP |
| Heshmat, 2012 [67], Iran |  |  |  |  | X |  |  | X | N= 42 (36% F) patients with type 2 diabetes.  Mean age 56±10 yr.  IC-25OHD < 50 nmol/L: No  B-25OHD: 103±35 nmol/L  Δ 25OHD > 50%: No | 3 mo. of follow-up after (DB):  A single intramuscular injection of D3 300,000 IU  Vs.  Placebo | BP was a secondary endpoint  No effect on office SBP or DBP |
| Kjærgaard, 2012 [68], Norway |  |  |  |  | X |  |  |  | N= 243 (53% F) adults  Mean age 53±10 yr.  IC-25OHD < 50 nmol/L: No  B-25OHD: 47±16 nmol/L  Δ 25OHD > 50%: Yes | 6 mo. of oral treatment (DB) with:  D3 40 000 IU/week  Vs.  Placebo | BP was a secondary endpoint  No effect on office SBP or DBP |
| Muldowney, 2012 [69], Ireland |  |  |  |  | X |  | X |  | N= 394 (54% F) adults aged 20-40 yr. or > 63 yr.  IC-25OHD < 50 nmol/L: No  B-25OHD: 70.4 nmol/L (young group) and 54.2 nmol/L (old group)  Δ 25OHD > 50%: No | 22 weeks of treatment (DB) with:  D3 200 IU/d  Vs.  D3 400 IU/d  Vs.  D3 600 IU/d  Vs.  Placebo | BP was a secondary endpoint  No effect on office SBP or DBP |
| Salehpour, 2012 [70]. Iran |  |  |  |  | X | X | X |  | N= 85 healthy premenopausal overweight and obese women aged 18–50 yr.  Mean age 38±8 yr.  .  IC-25OHD < 50 nmol/L: No  B-25OHD: 42±30 nmol/L  Δ 25OHD > 50%: No | 12 weeks of therapy (DB) with:  D3 1000 IU/d  Vs.  Placebo | BP was a secondary endpoint  No effect on office SBP or DBP |
| Stricker, 2012 [71], Switzerland |  |  |  |  | X |  | X |  | N= 62 (39% F) with chronic peripheral vascular disease and 25OHD < 75 nmol/L  Mean age 73±9 yr.  IC-25OHD < 50 nmol/L: No  B-25OHD: 43±15 nmol/L  Δ 25OHD > 50%: Yes | 1 mo. of follow-up after (DB):  D3 as a single, oral dose of 100 000 IU  VS.  Placebo | BP was a secondary endpoint  No effect on office SBP or DBP |
| Witham, 2012 [72], Scotland |  |  |  | X | X |  |  |  | N=58 (72% F) community dwelling patients with a history of stroke and baseline 25OHD <75 nmol/L  Mean age 67±10 yr.  IC-25OHD < 50 nmol/L: No  B-25OHD: 38±18 nmol/L  Δ 25OHD > 50%: No | 4 mo. of follow-up after (DB) a single oral dose of:  D2 100,000 units  Vs.  Placebo | BP was primary endpoint  No effect on office SBP or DBP |
| Wood, 2012 [73], UK |  |  |  | X | X |  | X |  | N=305 healthy postmenopausal women aged 60-70 yr.  Mean age 64 yr.  IC-25OHD < 50 nmol/L: No  B-25OHD: 34±16 nmol/L  Δ 25OHD > 50%: No | 1 year of therapy (DB) with:  D3 400 IU/d  Vs  D3 1000 IU/d  Vs.  Placebo | BP was a co-primary endpoint  No effect on office SBP or DBP |
| Asemi, 2013 [74], Iran |  |  |  |  | X |  |  |  | N= 54 pregnant women aged 18–40 yr. with gestational diabetes mellitus  Mean age 32±6 yr.  IC-25OHD < 50 nmol/L: No  B-25OHD: 51±35 nmol/L  Δ 25OHD > 50%: Yes | At baseline and at day 21, treatment with (DB):  D3 50,000 IU  Vs.  Placebo | BP was a secondary outcome  D3 reduced SPB significantly compared with placebo (according to reference [49]) |
| Boxer, 2013 [75], USA |  |  |  |  | X |  |  |  | N= 64 (48% F) patients with heart failure and 25OHD <94 nmol/L.  Mean age 66±10 yr.  IC-25OHD < 50 nmol/L: No  B-25OHD: 48±23 nmol/L  Δ 25OHD > 50%: Yes | 6 mo. of therapy (DB) with:  Vitamin D3 50,000 IU/week  Vs.  Placebo/week | BP was a secondary endpoint  No effect on office BP (according to reference [49]) |
| Breslavsky, 2013 [76], Israel  Also reported in [77] |  |  |  |  | X |  |  | X | N=47 (53% F) with T2D and cardiovascular risk factors recruited from a hypertension outpatient clinic.  Mean age 66±9 yr.  IC-25OHD < 50 nmol/L: No  B-25OHD: 44±28 nmol/L  Δ 25OHD > 50%: No | 1 year of therapy (DB) with:  D3 1000 IU/d  Vs.  Placebo | BP was a secondary endpoint  No effect on office BP (as reported in reference [77]) |
| Chai, 2013 [78], USA |  |  |  |  | X |  | X |  | N=92 (30% F) colorectal adenoma patients  Mean age 66±10 yr.  IC-25OHD < 50 nmol/L: No  B-25OHD: NA  Δ 25OHD > 50%: NA | 6 mo. of therapy (DB) with:  Calcium carbonate 2g/d  Vs.  D3 800 IU/d  Vs.  Calcium carbonate 2g/day +  D3 800 IU/d  Vs.  Placebo | BP was a secondary endpoint  No effect on office SBP or DBP |
| Forman, 2013 [79], USA |  |  |  | X | X |  | X |  | N= 283 (65% F) healthy blacks aged 30 to 80 years.  Median age (IQR): 51 (44–59) yr.  IC-25OHD < 50 nmol/L: No  B-25OHD, median (IQR): 39 (27-59) nmol/L  Δ 25OHD > 50%: NA | 3 mo. of therapy (DB) with:  D3 1000 IU/d + 200 mg Ca/d  Vs.  D3 2000 IU/d + 200 mg Ca/d  Vs.  4000 IU/d + 200 mg Ca/d  Vs.  Placebo + 200 mg Ca/d | BP was primary endpoint  In response to D3, SBP decreased dose-dependently: For each additional 1000 IU/d of D3, SBP decreased significantly by 1.4 mm Hg (P=0.04).  No effect on office DBP |
| Petchey, 2013 [80], AU |  |  |  |  | X |  |  |  | N=28 (29% F) adult patients with CKD stages 3–4 recruited from an outpatient clinic.  Mean age: 66±10 yr.  IC-25OHD < 50 nmol/L: No  B-25OHD: 90±29 nmol/L  Δ 25OHD > 50%: Yes | 6 mo. of therapy (DB) with:  D3 2000 IU/d  Vs.  Placebo | BP was a secondary endpoint  No effect on office BP (according to reference [49]) |
| Roth, 2013 [81], Bangladesh |  |  |  |  | X |  |  |  | N=160 pregnant women in 3^rd^ trimester aged 18 to <35 years, at 26 to 29 weeks gestation, and residing in Dhaka, Bangladesh.  Mean age: 22±3 yr.  IC-25OHD < 50 nmol/L: No  B-25OHD: 44±21 nmol/L  Δ 25OHD > 50%: Yes | Oral treatment (DB) until delivery:  D3 35,000 IU/week  Vs.  Placebo | BP was a secondary endpoint  No effect on office BP (according to reference [49]) |
| Toxqui, 2013 [82], Spain |  |  |  |  | X |  |  |  | N=99 Healthy, 18–35 years old, non-smoking, non-pregnant, and non-breast-feeding Caucasian women with low iron stores.  Mean age: 22±3 yr.  IC-25OHD < 50 nmol/L: No  B-25OHD: 63±21 nmol/L  Δ 25OHD > 50%: No | 4 mo. of therapy (DB) with:  Skimmed milk fortified with iron and D3 200 IU/d  Vs.  Skimmed milk fortified with iron but no D3 (Placebo) | BP was a secondary endpoint  No effect on office BP |
| Wamberg, 2013 [83], Denmark |  |  |  | X | X |  |  |  | N= 52 (71% F) with BMI > 30 kg/m2, aged 18 to 50, and with 25OHD <50 nmol/L.  Mean age: 40±7 yr.  IC-25OHD < 50 nmol/L: Yes  B-25OHD: 33±10 nmol/L  Δ 25OHD > 50%: Yes | 26 weeks of therapy (DB) with:  D3 7000 IU/d  Vs.  Placebo | BP was a co-primary endpoint  No effect on office BP |
| Witham, 2013 [26], Scotland |  |  |  | X | X |  | X |  | N=159 (48% F) with isolated systolic hypertension, aged > 70 years,  and with 25OHD < 75 nmol/L  Mean age: 77±7 yr.  IC-25OHD < 50 nmol/L: No  B-25OHD: 45±15 nmol/L  Δ 25OHD > 50%: No | 1 year of therapy (DB) with:  D3 100,000 IU evert 3 mo.  Vs.  Placebo | BP was primary endpoint  No effect on office BP or 24h BP |
| Witham, 2013 [84], Scotland |  |  |  |  | X |  | X |  | N=75 (33% F) with a history of myocardial  Infarction more than 6 weeks prior to inclusion.  Mean age: 66±10 yr.  IC-25OHD < 50 nmol/L: No  B-25OHD: 47±18 nmol/L  Δ 25OHD > 50%: No | 6 mo. of therapy (DB) with:  D3 100,000 IU oral at baseline and after 2 and 4 months.  Vs.  Placebo | BP was a secondary endpoint  No effect on office BP |
| Witham, 2013 [85], Scotland |  |  |  | X | X |  |  |  | N=50 South-east Asian women living in UK with 25OHD< 75 nmol/L.  Mean age: 40±12 yr.  IC-25OHD < 50 nmol/L: No  B-25OHD: 27±14 nmol/L  Δ 25OHD > 50%: No | 8 weeks of follow-up after (DB) a single dose of:  D3 100,000 IU  VS.  Placebo | BP was a secondary endpoint  No effect on office BP |
| Yiu, 2013 [86], Hong Kong |  |  |  |  | X |  | X | X | N= 100 (50% F) T2D patients with 25OHD< 75 nmol/L. recruited from a medical outpatient clinic  Mean age: 65±8 yr.  IC-25OHD < 50 nmol/L: No  B-25OHD: 54±11 nmol/L  Δ 25OHD > 50%: Yes | 12 weeks of therapy (DB) with:  D3 5000 IU/d  Vs.  Placebo | BP was a secondary endpoint  No effect on office BP |
| Moghassemi, 2014 [87], Iran |  |  |  |  |  |  |  |  | N=76 healthy post-menopausal women with 25OHD <75 nmol/L.  Mean age: 52±7 yr.  IC-25OHD < 50 nmol/L: No  B-25OHD: 34±10 nmol/L  Δ 25OHD > 50%: NA | 12 weeks of therapy (DB) with:  D3 2000 IU/d  Vs.  Placebo | BP was a secondary endpoint  No effect on office BP |
| Dalbeni, 2014 [88], Italy |  |  |  |  | X |  |  |  | N=36 (39% F) Chronic heart failure aged >40 yr with 25OHD< 75 nmol/L.  Mean age: 74±2 yr.  IC-25OHD < 50 nmol/L: No  B-25OHD: 44 nmol/L  Δ 25OHD > 50%: Yes | 6 mo. of therapy (DB) with:  D3 600,000 IU at baseline + 100,000 IU after 10 and 20 wk.  Vs.  Placebo | BP was a secondary endpoint  No effect on office BP |
| Scragg, 2014 [89], NZ |  |  |  |  | X |  | X |  | N=322 (75% F) adults recruited from staff and students.  Mean age: 48±10 yr.  IC-25OHD < 50 nmol/L: No  B-25OHD: 73±22 nmol/L  Δ 25OHD > 50%: Yes | 18 mo. of therapy (DB):  D3 200000 IU first mo., 200000 IU 2^nd^ mo. then 100000 IU monthly  Vs.  Placebo | BP was a secondary endpoint  No effect on office BP |
| Sollid, 2014 [90], Norway |  |  |  |  | X |  |  |  | N=511 (39% F) subjects with prediabetes aged 21–80 yr.  Mean age: 62±9 yr.  IC-25OHD < 50 nmol/L: No  B-25OHD: 60±21 nmol/L  Δ 25OHD > 50%: Yes | 1 yr. of therapy (DB):  D3 20,000 IU/wk  Vs.  Placebo | BP was a secondary endpoint  No effect on office BP  Subanalysis on subjects with baseline 25OHD <50 nmol/L did not change the results |
| Strobel, 2014 [91], Germany |  |  |  |  | X |  |  |  | N=86 (44% F) T2D patients not on insulin therapy aged 18–80 yr.  Mean age: 60 yr.  IC-25OHD < 50 nmol/L: No  B-25OHD: 30 nmol/L  Δ 25OHD > 50%: No | 6 mo. of therapy (DB):  D3 oral 13,322 IU/wk.  Vs.  Placebo | BP was a secondary endpoint  No effect on office BP |
| Raja-Khan, 2014 [92], USA |  |  |  |  |  |  | X |  | N=22 women with polycystic ovary syndrome aged 18-45 yr.  Mean age: 28±5 yr.  IC-25OHD < 50 nmol/L: No  B-25OHD: 50±25 nmol/L  Δ 25OHD > 50%: Yes | 12 weeks of therapy (DB):  D3 12,000 IU/d  Vs.  Placebo | BP was a secondary endpoint  No effect on office BP |
| Chen, 2014 [93], China |  |  |  |  |  |  | X |  | N=126 (42% F) with mild essential hypertension  Mean age: 62±9 yr.  IC-25OHD < 50 nmol/L: No  B-25OHD: 49±29 nmol/L  Δ 25OHD > 50%: Yes | 6 mo. of therapy (DB):  D3 2000 IU/d.  Vs.  Placebo  All patients were on nifedipine treatment 30 mg/d | BP was primary endpoint  Compared with placebo, D3 significantly reduced 24h daytime and night-time SBP and DBP |
| Witham, 2014 [94], Scotland |  |  |  |  | X |  |  |  | N=68 (35% F) with resistant hypertension defined as office SBP >140/90 mm Hg or on ≥3 anti-hypertensive agents with 25OHD < 75 nmol/L  Mean age: 63±11 yr.  IC-25OHD < 50 nmol/L: No  B-25OHD: 42±16 nmol/L  Δ 25OHD > 50%: NA | 6 mo. of therapy (DB):  D3 oral 100,000 IU every 2^nd^ mo.  Vs.  Placebo | BP was a secondary endpoint  No effect on office BP |
| Ryu, 2014 [95], Korea |  |  |  |  |  |  |  | X | N=81 (?% F) patients with T2D and 25OHD < 50 nmol/L.  Mean age: 55±7 yr.  IC-25OHD < 50 nmol/L: Yes  B-25OHD: 28±8 nmol/L  Δ 25OHD > 50%: Yes | 12 weeks of therapy (DB):  D3 2000 IU/d + Ca 200 mg/d  Vs.  Ca 200 mg/d | BP was a secondary endpoint  No effect on office BP |
| Jehle, 2014 [96], Switzerland |  |  |  |  |  |  |  | X | N=55 (64% F) patients with T2D for more than 10 yr.  Mean age: 66±9 yr.  IC-25OHD < 50 nmol/L: No  B-25OHD: 32±17 nmol/L  Δ 25OHD > 50%: Yes | 6 mo. of follow-up after (DB) a single dose of:  D3 300,000 IU IM + D3 150,000 IU IM if 25OHD was < 80 nmol/L after 3 mo.  Vs.  Placebo | BP was a secondary endpoint  No effect on 24h ambulatory BP |
| Kampmann, 2014 [97], Denmark |  |  |  |  |  |  |  | X | N=16 (50% F) patients with T2D and 25OHD < 50 nmol/L.  Mean age: 60±4 yr.  IC-25OHD < 50 nmol/L: Yes  B-25OHD: 31±5 nmol/L  Δ 25OHD > 50%: Yes | 12 weeks of therapy (DB):  D3 11,200 IU/d for 2 wk. followed by 5,600 IU/d for 10 wk.  Vs.  Placebo | BP was a secondary endpoint  No effect on office BP |
| Nasri, 2014 [98], Iran |  |  |  |  |  |  | X |  | N=60 (72% F) patients with T2D  Mean age: 55±11 yr.  IC-25OHD < 50 nmol/L: No  B-25OHD: 82±52 nmol/L  Δ 25OHD > 50%: Yes | 12 weeks of therapy (DB):  D3 50,000 IU/wk.  Vs.  Placebo | BP was primary endpoint  No effect on office BP |
| Tabesh, 2014 [99], Iran  Similar data published in [100] |  |  |  |  |  |  | X | X | N=118 (51% F) patients with T2D aged >30 years with 25OHD <75 nmol/L.  Mean age: 50±6 yr.  IC-25OHD < 50 nmol/L: No  B-25OHD: NA  Δ 25OHD > 50%: NA | 12 weeks of therapy (DB):  D3 50,000 IU/wk. + Ca-placebo  Vs.  D3-placebo + Ca 1,000 mg/d  Vs.  D3 50,000 IU/wk. + Ca 1,000 mg/d  Vs.  D3-placebo + Ca-placebo | BP was a secondary endpoint  Compared with the 3 other groups, CaD resulted in a greater reduction in SBP (p = 0.005) with no effect on DBP. |
| Sadiya, 2015 [101], UAE |  |  |  |  |  |  | X |  | N= 87 (82% F) obese patients with T2D aged 30-60 yr. with a BMI > 30 kg/m^2^, and 25OHD < 50 nmol/L.  Mean age: 48±8 yr.  IC-25OHD < 50 nmol/L: Yes  B-25OHD: 29±10 nmol/L  Δ 25OHD > 50%: Yes | 6 mo. of therapy (DB):  D3 6000 IU/d the first 3 mo. followed by 3000 IU/d the last 3 mo.  Vs.  Placebo | BP was a secondary endpoint  No effect on office BP |
| Pilz, 2015 [102], Austria |  |  |  |  |  |  | X |  | N=200 (47% F) with hypertension and 25OHD <75 nmol/L.  Mean age: 60±11 yr.  IC-25OHD < 50 nmol/L: No  B-25OHD: 53±14 nmol/L  Δ 25OHD > 50%: Yes | 8 weeks of therapy (DB):  D3 2800 IU/d  Vs.  Placebo | BP was primary endpoint  No effect on 24h ambulatory BP |
| Mozaffari-Khosravi [103], Iran |  |  |  |  |  |  | X |  | N=39 (64% F) aged 25–50 yr. with hypertension and 25OHD <75 nmol/L.  Mean age: 43±6 yr.  IC-25OHD < 50 nmol/L: No  B-25OHD: 45±18 nmol/L  Δ 25OHD > 50%: Yes | 8 weeks of oral therapy (DB):  D3 50,000 IU/wk  Vs.  Placebo | BP was primary endpoint.  D3 decreased office SBP and DBP significantly. |

*) MA BP4 did not specified included studies.

## Table Ca. Systematic reviews on risk of type 2 diabetes

SR with MA reporting summary data on findings from RCT on effects of vitamin D supplementation on risk of incident type 2 diabetes (T2D)

| **Systematic reviews on risk of type 2 diabetes** | | | | |
| --- | --- | --- | --- | --- |
| **MA#** | **Reference, publication year** | **Papers included** | **Criteria for inclusion of studies** | **Results** |
| DM1 | Seida, 2014 [104] | 4 RCT with 39,111 participants [105–108] | A systematic search of the literature on RCT examining the effects of vitamin D3 supplementation with or without calcium compared with placebo or other non-VitD supplementation in adults without known diabetes were included  Excluded trials examining active or synthetic VitD formulations or enrolling patients with type 1 or gestational diabetes, as well as trials with VitD2 | No effect of VitD: OR 1.02 (95% CI, 0.94 to 1.10, I^2^=0%)  Three studies reported progression to impaired glucose tolerance or diabetes in patients with normal glucose levels at baseline: OR 1.02 (95% CI, 0.94 to 1.10; I^2^=0%). |

## Table Cb. RCTs on risk of type 2 diabetes.

Characteristics of randomized clinical trials included in MA (Table 4A) on effects of vitamin D supplementation on risk of type 2 diabetes.

| **Characteristics of RCTs included in MAs on effects of vitamin D supplementation on risk of type 2 diabetes** | | | | |
| --- | --- | --- | --- | --- |
| **Reference, publication year, country** | **Included in MA** | **Population**  No. of randomized (% females), Characteristics of study | **Intervention** | **Results** |
|  | DM1 |  |  |  |
| Pittas, 2007 [105], USA | X | N=314 (70% F) healthy, non-diabetic community-dwelling adults, aged >65 years  Mean age ≈71±1 yr.  IC-25OHD <50 nmol/L: No  B-25OHD: ≈76±5 nmol/L  Δ 25OHD > 50%: No | 3 yr. of treatment (DB):  D3 700 IU + Ca 500 mg/d  Vs.  Placebo | Incident T2D was not a primary endpoint.  Results analyzed by glucose status at baseline – divided into whether participants had a NFG (N=222) or an IFG (N=92)  Among participants with an IFG, a similar number of patients (p=0.28) remained with IFG or had developed T2D at EOS in the CaD group (70%) and in the placebo group (81%)  The proportion of participants with NFG who progressed to IFG or diabetes was similar (p=0.84) in the placebo (19%) and in the CaD group (20%). |
| de Boer, 2008 [106], WHI, USA | X | N=36,282 postmenopausal women  IC-25OHD <50 nmol/L: No  B-25OHD: NR  Δ 25OHD > 50%: NR  Assessed self-reported incidence of T2D during the study. | 7 years of treatment (DB):  D3 400 IU + Ca 1000 mg/d  Vs.  Placebo | Incident T2D was not a primary endpoint.  Incidence of diabetes: HR 1.01 (95% CI, 0.94 to 1.10)  The null result was robust in subgroup analyses, efficacy analyses accounting for non-adherence, and analyses examining change in laboratory measurements |
| Avenell, 2009 [107], UK | X | N=5,292 (85% women) aged > 70 yr. with a previous low-trauma fracture.  IC-25OHD <50 nmol/L: No  B-25OHD: 38±16 nmol/L (subgroup)  Δ 25OHD > 50%: Yes (subgroup after one year of treatment)  Assessed self-reported incidence of T2D during the study. | Mean 45 (range 24-62) mo. of treatment (DB):  D3 800 IU/d  Vs.  Ca 1000 mg/d  Vs.  D3 800 IU/d + Ca 1000 mg/d  Vs.  Placebo. | Incident T2D was not a primary endpoint.  No difference in the incidence of self-reported incident T2D:  Intention-to-treat analyses:  HR 1.11 (95% CI, 0.77 to 1.62)  Per protocol analyses (good compliance):  HR 0.68 (95% CI, 0.40 to 1.16) |
| Davidson, 2013 [108], USA | X | N=109 (67% F) with pre-diabetes, aged > 40 yr.  IC-25OHD <50 nmol/L: No  B-25OHD: ≈55 nmol/L  Δ 25OHD > 50%: Yes | 1 yr. of treatment (DB):  D3: mean 88,865±16,154 (range 64,731–134,446) IU/week (individually titrated).  Vs.  Placebo/week | Development of diabetes was a co-primary endpoint  No effect of VitD supplementation on risk of developing T2D: OR: 1.37 (95% CI, 0.41 to 4.62) |

## Table Da. Systematic reviews on body weight

SR with MA reporting summary data on findings from RCT on effects of vitamin D supplementation on body weight.

| **Systematic reviews: Body weight** | | | | |
| --- | --- | --- | --- | --- |
| **#MA** | **Reference, publication year** | **Papers included** | **Criteria for inclusion of studies** | **Results** |
| BV1 | Pathak, 2014 [109] | 12 RCT with N=1,421 participants  [29,45,46,51,59,61,83,110–114] | A systematic searched on RCTs published from 1995-2013 that had supplemented with VitD without imposing any caloric restriction.  Included only RCTs of high quality conducted on adult non-pregnant, non-lactating women.  18 trials were identified reporting effects on either body weight, BMI, fat mass (FM), percentage fat mass (%FM) or lean body mass (LBM). However, only 12 studies provided the required data for a MA. | VitD supplementation did not affect the SMD for body weight, FM, %FM or LBM.  A small but non-significant decrease in BMI (SMD = −0.097, 95% CI, −0.210 to 0.016], P = 0.09) was observed.  Meta-regression confirmed that neither the absolute VitD status achieved nor its change from baseline influenced the SMD of any obesity measure. |
| BW2 | Chandler, 2015 [115] | 26 RCTs with N=42,430 participants  VitD alone vs. Placebo: 12 RCTs  CaD vs. Ca: 10 RCTs  CaD vs. Placebo: 5 RCTs  [3,22,29,41,46,51,53,60,68,73,83,105,108,110–112,116–125] | To assess whether VitD and Ca supplements cause changes in adiposity.  RCTs with >50 participants aged >18 years at baseline who had received at least 12 weeks of treatment. | VitD vs. Placebo: no significant effect on  BMI (WMD -0.06 kg/m^2^ (95% CI, -0.14 to 0.03)  Body weight: WMD -0.05 kg (95% CI, -0.32 to 0.23)  Fat mass: WMD -0.43 kg (95% CI, -1.69 to 0.84).  CaD vs. Ca: no significant effect on  BMI: WMD, 0.02 kg/m^2 (^95% CI, -0.11 to 0.14)  Body weight: WMD, 0.12 kg (95% CI, -0.24 to 0.49)  Fat mass: WMD, 0.12 kg (95% CI, -0.22 to 0.45)  CaD vs. Placebo:  BMI: WMD, -0.03 (95% CI, -0.13 to 0.08)  **Body weight: WMD, -0.13 (**95% CI, **-0.21 to -0.05)**  Fat mass: WMD, 0.67 (95% CI, -0.33 to 1.66) |
| BV3 | Manousopoulou, 2015 [50] | 6 RCTs  [45,46,51,52,83,125] | Only RCTs including adults with obesity  Interventions in addition to VitD (e.g. calcium co-administration, exercise program, weight loss regime) applied equally to both groups. | No effect of VitD on  - Body weight: SMD 0.10; 95% CI, -0.11 to 0.31), I^2 =^ 57%  - BMI: SMD = −0·047; (95% CI, −0·178 to 0·084, I^2^ = 0% |

## Table Db. RCTs on body weight

Characteristics of randomized clinical trials included in MA (Table 5A) on effects of vitamin D supplementation on body weight

| **Characteristics of RCTs included in MAs on effects of vitamin D supplementation on body weight** | | | | | | |
| --- | --- | --- | --- | --- | --- | --- |
| **Reference, publication year, country** | **Included in MA** | | | **Population**  No. of randomized (% females), Characteristics of study | **Intervention** | **Results** |
|  | BW1 | BW2 | BW3 |  |  |  |
| Ljunghall, 1987 [116], Sweden |  | X |  | 65 middle-aged men with impaired glucose tolerance  Mean age NA yr.  IC-25OHD $<$ 50 nmol/L: NA  B-25OHD: NA  Δ 25OHD > 50%: NA | 3 mo. of treatment (DB):  0.75 micrograms alphacalcidol/d  Vs.  Placebo | Weight loss was not a primary end-point.  Subjects treated with alphacalcidol displayed a significant reduction in body weight with an average of 1.1 kg, while those receiving placebo lost no weight |
| Dawson-Hughes, 1991 [117], USA |  | X |  | N=249 healthy ambulatory post-menopausal women  Mean age 61±0.5 yr.  IC-25OHD $<$ 50 nmol/L: No  B-25OHD: NA  Δ 25OHD > 50%: NA | 12 mo. treatment (DB):  D3 400 IU/d + Ca 377 mg/d  Vs  D3 placebo + Ca 377 mg/d | Weight loss was not a primary end-point.  No significant differences in the outcome variables between the two groups |
| Heikkinen, 1997 [118], Finland |  | X |  | N=464 postmenopausal women aged 47-56-yr.  Mean age 53±1 yr.  IC-25OHD $<$ 50 nmol/L: No  B-25OHD: NA  Δ 25OHD > 50%: NA | 3 yr. of treatment (DB):  HRT  Vs.  D3 300 IU/d  Vs.  HRT+D3 300 IU/d  Vs.  Placebo (calcium 93 mg/d). | Weight loss was not a primary end-point.  No effect on body weight  D3 seemed to affect serum lipid concentrations unfavorably and possibly counteracted positive lipid profile changes during HRT. |
| Dawson-Hughes, 1997 [119], USA |  | X |  | N=389 (55% F) healthy ambulatory participants  Mean age 61.9 yr.  IC-25OHD $<$ 50 nmol/L: No  B-25OHD: 72 nmol/L  Δ 25OHD > 50%: No | Up to 36 mo treatment (DB) with  D3 700 IU/d + Ca 500 mg/d  Vs.  Placebo | Weight loss was not a primary end-point.  No reports on effect on body weight |
| Aloia, 2005 [120], USA |  | X |  | N=208 black postmenopausal African-American women.  Mean age 61±6 yr.  IC-25OHD $<$ 50 nmol/L: No  B-25OHD: 48 nmol/L  Δ 25OHD > 50%: No | 36 mo. of treatment (DB):  D3 800 IU/d (the first two years), 2000 IU/d (the third year) + Ca 1200-1500 mg/d  Vs.  Placebo plus Ca 1200-1500 mg/d | Weight loss was not a primary end-point.  No effect on body weight |
| Caan, 2007 [121], USA |  | X |  | N= 36 282 post-menopausal women  IC-25OHD < 50 nmol/L: No  B-25OHD: NR  Δ 25OHD > 50%: NR | 7 yr. of treatment (DB):  D3 400 IU + Ca 1000 mg/d  Vs.  Placebo | Weight loss was not a primary end-point.  Compared with placebo, CaD resulted in a significantly decreased body weight −0.13 kg (95% CI, −0.21 to −0.05; P =0.001). |
| Major, 2007 [41], CA |  | X |  | N=63 healthy obese/overweight normotensive (BP <160/95mm Hg) premenopausal women with a Ca intake < 800 mg/d  Mean age 44±5 yr.  IC-25OHD < 50 nmol/L: No  B-25OHD: NR  Δ 25OHD > 50%: NR | 15 weeks of treatment (DB):  D3 400 IU/d + Ca 1200 mg/d  Vs.  Placebo  Both groups observed a 700 kcal/d energy restriction | Weight loss was not a primary end-point.  No significant effect on body weight |
| Pittas, 2007 [105], USA |  | X |  | N=314 (70% F) healthy, non-diabetic community-dwelling adults, aged >65 years  Mean age ≈71±1 yr.  IC-25OHD < 50 nmol/L: No  B-25OHD: ≈76±5 nmol/L  Δ 25OHD > 50%: No | 3 yr. of treatment (DB):  D3 700 IU + Ca 500 mg/d  Vs.  Placebo | Weight loss was not a primary end-point.  No significant effect on body weight |
| Prince, 2008 [3], AU |  | X |  | 302 community-dwelling ambulant women with a history of falling in the past 12 months, aged 70-90 yr.  Mean age ≈77±5 yr.  IC-25OHD < 50 nmol/L: No  B-25OHD: ≈45±12 nmol/L  Δ 25OHD > 50%: No | 12 mo. of treatment (DB):  D2 1000 IU + Ca 1000 mg/d  Vs.  Placebo + Ca 1000 mg/d | Weight loss was not a primary end-point.  No significant effect on body weight |
| Zhu, 2008 [22], AU |  | X |  | 120 community-dwelling women aged 70–80 yr.  Mean age 74.8±2.6 yr.  IC-25OHD < 50 nmol/L: No  B-25OHD 68±29 nmol/L  Δ 25OHD > 50%: Yes | 5 yr. of treatment (DB):  D2 1000 IU + Ca 1200 mg/d  Vs.  Placebo-VitD + placebo-Ca | Weight loss was not a primary end-point.  No significant effect on body weight |
| Sneve, 2008 [110], Norway | X | X |  | N=445 (64% F) healthy, overweight and obese, aged 21–70 yr.  Mean age 48±11 yr.  IC-25OHD < 50 nmol/L: No  B-25OHD: 53±17 nmol/L  Δ 25OHD > 50%: Yes | 12 mo. of treatment (DB)  D3 20 000 IU twice a week  Vs.  D3 20 000 IU once a week  Vs.  placebo.  + Ca 500 mg/d to all subjects | Primary end-point: weight loss  No significant changes in weight, waist-to-hip ratio or percentage body fat in any of the groups, nor between them. |
| Zittermann, 2009 [46], Germany | X | X | X | N=165 (67% F) healthy overweight subjects recruited from the background population. Excluded subjects with CVD.  Mean age: 48±10 yr.  IC-25OHD < 50 nmol/L: No  B-25OHD: 30±19 nmol/L  Δ 25OHD > 50%: Yes | 12 mo. of treatment (DB):  D3 3320 IU/d  Vs.  Placebo  All participated also in a weight-reduction program. | Primary end-point: weight loss  Weight loss was not affected significantly by VitD supplementation (-5.7±5.8 kg) or placebo (-6.4±5.6 kg).  SMD -0.10 (95% Cl -0.20 to 0.41). |
| Nagpal, 2009, [45], India | X |  | X | N=71 apparently healthy men with centrally obesity, aged ≥ 35 yr.  Excluded men with hypertension.  IC-25OHD < 50 nmol/L: No  B-25OHD: 33±13 nmol/L  Δ 25OHD > 50%: Yes | 3 oral doses administrated 2 weeks apart (DB):  D3 120 000 IU  Vs.  Placebo | Weight loss *was not* a primary end-point  6 weeks after baseline (2 weeks after last dose):  No effects on body weight or BMI: SMD 0.03 (95% Cl -0.43 to 0.50). |
| von Hurst, 2010 [111], NZ | X | X |  | N=81 South Asian women with insulin resistance, aged 23–68 yr.  Excluded women with diabetes and/or an intake of VitD > 1000 IU/d.  IC-25OHD < 50 nmol/L: Yes  B-25OHD: ≈20 nmol/L  Δ 25OHD > 50%: Yes | 6 mo. of treatment (DB):  D3 4000 IU/d  Vs.  Placebo | Weight loss *was not* a primary end-point.  BMI did not change significantly in either group during the study. SMD -0.06 (95% Cl -0.50 to 0.37). |
| Zhou, 2010 [122], USA |  | X |  | N= 870 postmenopausal women  Mean age: 66±7 yr.  IC-25OHD < 50 nmol/L: No  B-25OHD: 73±20 nmol/L  Δ 25OHD > 50%: No | 4 yr. of treatment (DB):  Ca-group: Ca 1400 or 1500 mg/d + D3-placebo  Vs.  CaD group: Ca 1400 or 1500 mg/d  + D3 1100 IU/d  Vs.  Ca-placebo + D3-placebo | Weight loss was not a primary end-point (secondary analysis of data from a population-based trial on effects of CaD on fractures, including N=1179 women).  No effect on BMI  Compared with placebo, the CaD- and Ca-groups gained less truncal fat and lost less truncal lean mass. |
| Jorde, 2010  [53], Norway |  | X |  | N= 438 (64% F) overweight or obese subjects without diabetes or IHD.  Mean aged 48±11 yr.  IC-25OHD $<$50 nmol/L: No  B-25OHD: 58±21 nmol/L  Δ 25OHD > 50%: Yes | 12 mo. Of treatment (DB)  D3 20,000 IU/week + 500mg Ca/d  Vs.  D3 40,000 IU/week + 500mg Ca/d  Vs.  Placebo + 500 mg Ca/d | Weight loss was not a primary end-point.  No significant effect on body weight |
| Grimnes, 2011 [112], Norway | X | X |  | N=104 (47 % F) Caucasians aged 30–75 yr.  IC-25OHD < 50 nmol/L: Yes  B-25OHD: 40 nmol/L  Δ 25OHD > 50%: Yes | 6 mo. of treatment (DB):  D3 20,000 IU twice weekly  Vs.  Placebo twice weekly | Weight loss *was not* a primary end-point.  No statistically significant differences in the outcome variables between the two groups |
| Maki, 2011 [52], USA |  |  | X | N=60 (75% F) aged 18–79 yr with high waist circumference.  Mean age 52±2 yr.  IC-25OHD $<$ 50 nmol/L: No  B-25OHD: 64±4 nmol/L  Δ 25OHD > 50%: No | 8 weeks of treatment (DB):  D3 1,200 IU/d + daily multivitamin and mineral supplement  Vs.  Daily multivitamin and mineral supplement without D3 | Weight loss was not a primary end-point.  D3 increased body weight significantly |
| Nikooyeh, 2011 [59], Iran | X |  |  | N=90 (F=61 %) aged 30-60 yr. with T2D  IC-25OHD < 50 nmol/L: No  B-25OHD: 42 nmol/L  Δ 25OHD > 50%: No | 3 mo of treatment (DB):  Ca 300 mg/d (yogurt)  Vs.  D3 1000 IU/d D3 + Ca 300 mg/d (yogurt)  Vs.  D3 1000IU/d 500 mg calcium (yogurt) | Weight loss *was not* a primary end-point.  No significant effect on BMI: SMD -0.22 (95% Cl -0.72 to 0.29).  . |
| Shab-Bidar, 2011 [61], Iran | X |  |  | N=100 (F=57 %) with T2D, BMI 28-30 kg/m^2^  IC-25OHD < 50 nmol/L: No  B-25OHD: 38 nmol/L  Δ 25OHD > 50%: No | 3 mo. Of treatment (DB):  D3 1000 IU/d + Ca 340 mg/d  Vs.  Ca 340 mg/d (yogurt) | Weight loss *was not* a primary end-point.  No significant effect on BMI: SMD -0.35 (95% Cl -0.75 to 0.04).  . |
| Harris, 2011 [60], USA |  | X |  | N=45 (53% F) African-American adults with no overt cardiovascular, pulmonary or metabolic disease  Mean age 30±2 yr.  IC-25OHD $<$ 50 nmol/L: No  B-25OHD: 36±3 nmol/L  Δ 25OHD > 50%: Yes | 16 weeks of treatment (DB):  D3 60,000 IU monthly  Vs.  Placebo | Weight loss was not a primary end-point.  No significant effect on body weight |
| Salehpour, 2012 [51], Iran | X | X | X | N= 77 (100% F) overweight and obese women with BMI 29.8±4.1kg/m^2^  IC-25OHD < 50 nmol/L No  B-25OHD: 41.8 nmol/L  Δ 25OHD > 50%: No | For 3 mo. Treatment (DB) with  D3 1000 IU/d  Vs.  placebo | Primary end-point: anthropometric  Body weight: SMD -0.15 (95% Cl -0.60 to 0.30) and waist circumference did not change significantly |
| Ardabili, 2012 [113], Iran | X |  |  | N=50 women with PCOS and a vitamin D deficiency aged 20-40 yr.  IC-25OHD < 50 nmol/L: Yes  B-25OHD: ≈ 17±7 nmol/L  Δ 25OHD > 50%: Yes | 2 mo.of treatment (DB):  D3 50,000 IU/every 20 day  Vs.  placebo | Weight loss *was not* a primary end-point.  No effect on body weight: SMD 0.00 (95% Cl -0.56 to 0.55). |
| Wood, 2012 [73], UK |  | X |  | N=305 healthy postmenopausal women aged 60-70 yr.  Mean age 64 yr.  IC-25OHD < 50 nmol/L: No  B-25OHD: 34±16 nmol/L  Δ 25OHD > 50%: No | 1 year of therapy (DB) with:  D3 400 IU/d  Vs.  D3 1000 IU/d  Vs.  Placebo | Weight loss was not a primary end-point.  No significant effect on body weight |
| Kjærgaard, 2012 [68], Norway |  | X |  | N= 243 (53% F) adults  Mean age 53±10 yr.  IC-25OHD < 50 nmol/L: No  B-25OHD: 47±16 nmol/L  Δ 25OHD > 50%: Yes | 6 mo. of oral treatment (DB) with:  D3 40 000 IU/week  Vs.  Placebo | Weight loss was not a primary end-point.  No significant effect on body weight |
| Glendenning, 2012 [123], AU |  | X |  | N=686 elderly community-dwelling ambulatory women  Mean age 76.9 ±4.0 yr.  IC-25OHD < 50 nmol/L: No  B-25OHD: 66±23 nmol/L (sub-group)  Δ 25OHD > 50%: No | 6 mo. of treatment (DB):  D3 150,000 IU/threemonthly  Vs  Placebo | Weight loss was not a primary end-point.  No significant effect on body weight |
| Gallagher, 2012 [29], USA | X | X |  | N=163 healthy postmenopausal white women aged 57–90 yr.  Mean age: 68±8 yr.  IC-25OHD < 50 nmol/L: Yes  B-25OHD: 39±10 nmol/L  Δ 25OHD > 50%: Yes | 1 year of treatment (DB):  Placebo  Vs  D3 400 IU /d  Vs.  D3 800/ IU d  Vs.  D3 1600 IU /d  Vs.  D3 2400 IU /d  Vs.  D3 3200 IU /d  Vs.  D3 4000 IU /d  Vs.  D3 4800 IU/d.  All received daily calcium to maintain intake of 1200–1400 mg | Weight loss *was not* a primary end-point.  No effect on body weight:  D3 400 IU: SMD -0.06 (95% Cl -0.71 to 0.60).  D3 800 IU: SMD 0.15 (95% Cl -0.51 to 0.80).  D3 1600 IU: SMD 0.11 (95% Cl -0.56 to 0.77).  D3 2400 IU: SMD 0.36 (95% Cl -0.28 to 1.00).  D3 3200 IU: SMD -0.11 (95% Cl -0.76 to, 0.54).  D3 4000 IU: SMD 0.64 (95% Cl -0.04 to 1.33).  D3 4800 IU: SMD -0.27 (95% Cl -0.92 to 0.39). |
| Carrillo, 2013 [114], USA | X |  |  | N=23 (52% F), obese, BMI 31.3 kg/m^2^  Age: 26.1± 4.7 yr.  IC-25OHD: < 50 nmol/L: No  B-25OHD: 48 nmol/L  Δ 25OHD > 50%: No | 3 mo. of treatment (DB):  D3 4000 IU/d  Vs  placebo  Both groups completed 12 weeks of resistance training | Primary end-points: body composition, muscle function and glucose tolerance.  No effect on BMI: SMD -0.08 (95% Cl -0.90 to 0.75). |
| Wamberg, 2013 [83], Denmark | X | X | X | N=52 (71 % F) aged 18–50 years with BMI more than 30 kg/m^2^ and low vitamin D levels.  IC-25OHD < 50 nmol/L: Yes  B-25OHD: 33 nmol/L  Δ 25OHD > 50%: Yes | 26 weeks of treatment (DB):  D3 7000 IU/d  Vs.  Placebo | Weight loss *was not* a primary end-point.  No effect on BMI: SMD 0.09 (95% Cl -0.51 to 0.69). |
| Davidson, 2013 [108], USA |  | X |  | N=109 (67% F) with pre-diabetes, aged > 40 yr.  IC-25OHD < 50 nmol/L: No  B-25OHD: ≈55 nmol/L  Δ 25OHD > 50%: Yes | 1 yr. of treatment (DB):  D3: mean 88,865±16,154 (range 64,731–134,446) IU/week (individually titrated).  Vs.  Placebo/week | Weight loss was not a primary end-point.  No significant effect on body weight |
| Chandler, 2014 [124], USA |  | X |  | N=328 (68% F) African Americans living in public housing communities in Boston, aged 30-80 yr.  Median age of 51 yr.  IC-25OHD < 50nmol/L: No  B-25OHD: median 38 nmol/L  Δ 25OHD > 50%: Yes | 3 mo. of treatment (DB):  D3 1,000 IU/d  Vs.  D3 2,000 IU/d  Vs.  D3 4,000 IU/d  Vs.  Placebo | Weight loss was not a primary end-point.  No significant effect on body weight |
| Mason, 2014 [125], USA |  | X | X | N=218 overweight/obese women aged 50–75 yr. with 25OHD between 25-80 nmol/L  Age 60±5 yr.    IC-25OHD < 50nmol/L: No  B-25OHD: 54±15 nmol/L  Δ 25OHD > 50%: Yes | 12 mo. of treatment (DB):  D3 2000 IU/d  Vs  Placebo  All participants were also assigned to a weight loss program | Weight loss was primary end-point  D3 did not affect changes in body weight, waist circumference, percentage body fat, or trunk fat mass. |

## Table Ea. Systematic reviews on birth weight

SR with MA reporting summary data on findings from RCT on effects of vitamin D supplementation on birth weight.

| **Systematic reviews on effects of vitamin D supplementation on birth weight** | | | | |
| --- | --- | --- | --- | --- |
| **#MA** | **Reference, publication year** | **Papers included** | **Criteria for inclusion of studies** | **Results** |
| BiW1 | Mahomed, 2000 [126] | Birth weight: 2 RCT with N=176 pregnant women [127,128]. | A Cochrane review assessing effects of VitD supplementation on pregnancy outcomes.  A systematic literature search was performed on any RCT on effects of VitD supplementation in pregnancy with clinical endpoints | *Birth weight* (comparing VitD 1000 IU/d with no supplement):   - WMD: -78 g (95% CI, -119 to -36 g, I^2^ =82%)   The authors of the review noted that the SD on mean birth weight reported in the study by Mallet et al. [128] is too small to be realistic (reported to be 70-90g whereas it normally is approximately 600 g). Probably the standard error has been reported instead of SD and this may have affected to pooled estimate of the MA. |
| BiW2 | Thorne-Lyman, 2012 [129] | *Low birth weight:* 3 RCT with N=507 pregnant women  [127,130,131]  Birth weight: 5 RCT with N=932 pregnant women  [127,128,130–132] |  | *Birth weight:*   - WMD: 58 g (95% CI, -64 to 180 g, I^2^ = 70%)   *Low birth weight*:   - RR = 0.40 (95% CI, 0.23 to 0.71, I^2^ = 5%) |
| BiW3 | De-Regil, 2012 [133] | *Risk of having a baby with a birth weight < 2500 grams:* 3 RCT with N=463 pregnant women [127,130,131].  *Birth weight:* 3 RCT with N=403 pregnant women [127,128,130] | An update of the Cochrane review by Mahomed [126] on effects of VitD supplementation on pregnancy outcome. A systematic literature search was performed allowing for inclusion of individually or cluster randomized trials, evaluating the effect of supplementation with VitD alone or in combination with other micronutrients for women during pregnancy | *Birth weight*:   - WMD: 40 g (95% CI, -241 to 320 g, I^2^ = 96%)   *Risk of birth weight < 2500 g:*   - RR 0.48; 95% CI, 0.23 to 1.01, I^2^=53% |
| BiW4 | Harvey, 2014 [134] | 9 RCT were identified, among which 6 RCT were included in MA:  *Low dose studies (5 RCT):*  [127,128,130,135,136]  *High dose studies (6 RCT):*  [127,128,130,135–137] | A systematic search was performed on RCT published in English and reporting data on the individual treatment groups.  MA stratified by total dose of VitD administered:  *Low dose* studies: total dose < 120,000 IU VitD  *High dose* studies: total dose > 120,000 IU VitD | *Birth weight*:  Low dose studies: WMD 116 (95% CI, -57 to 289) gram (I^2^ 86%)  High dose studies: WMD 147 (95% CI, -112 to 407) gram (I^2^ 96%) |
| BiW5 | Pérez-López, 2015 [138] | 8 RCTs with N=1489 participants [81,127,132,137,139–142] | RCTs on VitD supplementation used alone and in combination with calcium and vitamin supplements. Included RCTs of pregnant women of any gestational or chronologic age and parity, without previous disease history.  Excluded trials without an appropriate control group, trials with data on multiple pregnancies, and if data were not available or could not be extracted for the study groups. | *Birth weight:*  Mean difference 108 (95% CI, 60 to 155) gram, I^2^ 0% |
| BiW6 | De-Regil, 2016  [143] | Birth weight: 5 RCTs with N=715 women [81,127,128,137,144].  Low birth weight (<2500 g): 3 RCTs with N=493 women. | Cochrane review – An update on the review from 2012 [133]. | **Birth weight:**  5 RCTs (N=715 women): No difference of weight at birth in infants from women who received VitD in comparison with women who did not receive VitD (MD 67; 95% CI -137 to 270 gram, I^2^ 95%).  If the study by Mallet et al. [128] was excluded the heterogeneity was reduced and vitD increased birth weight significantly (MD 147, 95% CI 68 to 225 gram, I^2^ 34%, N=638 women).  *Birthweight below 2500 g:* 3 trials (N=493 women) showed a lower risk of having a baby with low birth weight in response to vitD supplementation (RR 0.40; 95% CI 0.24 to 0.67). |

## Table Eb. RCTs on birth weight.

Characteristics of randomized clinical trials included in MA (Table 6A) on effects of vitamin D supplementation on birth weight.

| **Characteristics of the RCTs included in MAs on effects of vitamin D supplementation on birth weight** | | | | | | | | | |
| --- | --- | --- | --- | --- | --- | --- | --- | --- | --- |
| **Reference, publication year, country** | **Included in MA** | | | | | | **Population**  No. of randomized (% females), Characteristics of study | **Intervention** | **Results** |
|  | **BiW1** | **BiW2** | **BiW3** | **BiW4** | **BiW5** | **BiW6** |  |  |  |
| Brooke et al, 1980 [127], UK | X | X | X | X | X | X | N=126 first-generation Asian immigrants living in London:  IC-25OHD <50 nmol/L: No  B-25OHD: 20 nmol/L  Δ 25OHD > 50%: Yes | Treated during 3^rd^ trimester (DB):  D2 1000 IU/d  vs.  Placebo | Fetal growth was co-primary endpoint  No effects on birth weight |
| Marya, 1981 [130], India |  | X | X | X |  |  | N=120 women  IC-25OHD <50 nmol/L: No  B-25OHD: NR  Δ 25OHD > 50%: NR | Intervention during 3^rd^ trimester - randomized to (NB):  D2 1200 IU/d + Ca 375 mg/d  Vs.  D2 600,000 IU in 7^th^ and 8^th^ months of pregnancy  Vs.  No treatment | Birth weight was *not* primary endpoint  Birth weight increased significantly in response to vitamin D supplementation. |
| Congdon, 1983 [135], UK |  |  |  | X |  |  | N=64 pregnant Asian women in the UK  IC-25OHD <50 nmol/L: No  B-25OHD: NR  Δ 25OHD > 50%: NR | Intervention during 3^rd^ trimester - randomized to (NB):  D 1000 IU/d + Ca (dose not given)  Vs.  No treatment | Birth weight was *not* primary endpoint  No effects of supplementation on birth weight. |
| Mallet, 1986 [128], France | X | X | X | X |  | X | N=77 pregnant women:  IC-25OHD <50 nmol/L: No  B-25OHD: NR  Δ 25OHD > 50%: No | Treated from 7^th^ month of pregnancy until delivery with (NB):  D2, 1000 IU/d  Vs.  D2 200.000 IU (single dose)  Vs.  No treatment | Birth weight was not primary endpoint  No effects of supplementation on birth weight. |
| Marya, 1988 [137], India |  |  |  | X | X | X | N=200 with a singleton pregnancy attending an antenatal clinic  IC-25OHD <50 nmol/L: No  B-25OHD: NR  Δ 25OHD > 50%: NR | D3: two doses of 600,000 IU at  7^th^ and 8^th^ mo. of pregnancy  Vs.  No treatment | Primary endpoint: Birth weight  No effects of supplementation on birth weight. |
| Kaur, 1991 [136], India |  |  |  | X |  |  | N= 50 pregnant women:  IC-25OHD <50 nmol/L: No  B-25OHD: NR  Δ 25OHD > 50%: NR | Two doses of D3 60,000 IU (NB) at  6^th^ and 7^th^ mo. of pregnancy  Vs.  No treatment | Co-primary endpoint: Birth weight  Compared to controls, the birth weight was significantly higher in the supplemented group (p<0.001) |
| Yu, 2009 [131], UK |  | X | X |  |  |  | N= 180 pregnant women attending an antenatal clinic (routine visit):  IC-25OHD <50 nmol/L: No  B-25OHD: 26 nmol/L  Δ 25OHD > 50%: No | Treated from 27^th^ week of pregnancy until delivery (NB):  D2: a single dose of 200.000 IU  Vs.  D2 800 IU/d  Vs.  No treatment | Birth weight was *not* primary endpoint  No effects of supplementation on birth weight |
| Hollis, 2011 [132], USA |  | X |  |  | X |  | N= 350 with a singleton pregnancy  IC-25OHD <50 nmol/L: No  B-25OHD: ≈59 nmol/L  Δ 25OHD > 50%: Yes (in the group receiving 4000 IU/d) | Treated from 12 to 16 weeks' gestation until delivery (DB):  D3 400 IU/d  Vs.  D3 2000 IU/d  Vs.  D3 4000 IU/d | Birth weight was *not* primary endpoint  No effects of supplementation on birth weight |
| Sabet, 2012 [139], Iran |  |  |  |  | X |  | N=100 pregnant Iranian women  IC-25OHD <50 nmol/L: No  B-25OHD: NR  Δ 25OHD > 50%: NR | During the last trimester.  D3 100,000 IU/mo  Vs  Placebo | Birth weight was not primary endpoint  No effects of supplementation on birth weight |
| Goldring, 2013 [140], UK |  |  |  |  | X |  | N=158 women presenting at 27 weeks gestation for routine glucose challenge test from different ethnic groups (Asian, Middle Eastern, Black and White).  IC-25OHD <50 nmol/L: No  B-25OHD: NR  Δ 25OHD > 50%: NR | From 27 weeks gestation, stratified by 4 ethnic groups (NB):  D2 800 IU/d  Vs.  D3 a single oral dose of 200,000 IU  Vs.  No treatment | Birth weight was not primary endpoint  No effects of supplementation on birth weight |
| Roth, 2013 [81], Bangladesh |  |  |  |  | X | X | N=160 pregnant women aged 18-35 years, at 26-29 weeks gestation, and living in Bangladesh  IC-25OHD <50 nmol/L: No  B-25OHD: 45 nmol/L  Δ 25OHD > 50%: Yes | Treated from 26-29 wk. of gestation until delivery (DB):  D3 35,000 IU/wk.  Vs.  Placebo | Birth weight was not primary endpoint  No effects of supplementation on birth weight |
| Hashemipour, 2014, [141], Iran |  |  |  |  | X |  | N=130 pregnant women (24–26 weeks of gestation) with 25OHD < 75 nmol/L.  IC-25OHD <50 nmol/L: No  B-25OHD: ≈ 41±15 nmol/L  Δ 25OHD > 50%: NR | 8 weeks of therapy (NB):  D3 50,000 IU/wk. plus Ca 200 mg/d + multivitamin with D3 400 IU/d  Vs.  Ca 200 mg/d + multivitamin with D3 400 IU/d | Birth weight was a co-primary end-point.  Compared with low dose D3, high dose therapy increased birth weight significantly (p = 0.01) in the D3 group (mean 170; 95% CI, 41 to 299 g.). |
| Hossain, 2014 [142], Pakistan |  |  |  |  | X |  | N=200 healthy women at week 20 or less of gestation with a singleton pregnancy  IC-25OHD <50 nmol/L: No  B-25OHD: ≈13 nmol/L  Δ 25OHD > 50%: Yes | Started at 20 weeks and continued till delivery (NB):  D3 4000 IU/d + Ca 600 mg/d  Vs  Ca 600 mg/d | Birth weight was not primary endpoint  No effects of supplementation on birth weight |
| Sablok, 2015 [144], India |  |  |  |  |  | X | N=180 Primi-gravidae healthy women with singleton pregnancy at 14–20 weeks.  IC-25OHD <50 nmol/L: No  B-25OHD: NR  Δ 25OHD > 50%: NR | Treated from 14-20 week of pregnancy until delivery (NB):  N=120 supplementation according to baseline 25OHD levels: - If 25OHD>50 nmol/L: single dose of D3 60,000 IU at 20 weeks,  - If 25OHD 25-50 nmol/L: D3 120,000 IU at week 20 and 24.  - If 25OHD< 25 nmol/L: D3 120,000 IU at week 20, 24, 28 and 32.  Vs.  N=60 No intervention | Birth weight was a co-primary end-point  Mean birthweight was significantly (p=0.04) higher in the intervention group (2.6±0.4 kg) compared to the controls (2.4±0.31 kg) |

## Table Fa. Systematic reviews on risk of malignant diseases

SR with MA reporting summary data on findings from RCT on effects of vitamin D supplementation on risk of malignant diseases.

| **Systematic reviews on risk of malignant diseases** | | | | |
| --- | --- | --- | --- | --- |
| **#MA** | **Reference, publication year** | **Papers included** | **Criteria for inclusion of studies** | **Results** |
| C1 | Fortmann, 2013 [16] | 2 RCT on VitD alone: [2,145]  2 RCT on CaD: [15,18] | Included fair- and good-quality RCT assessing effects of supplements in the *primary prevention* of cancers in the general adult population (community dwelling) without chronic diseases. | Pooled unadjusted summary risk on risk of cancer:  VitD alone: RR 1.05 (0.94 to 1.18)  CaD: RR 0.98 (CI, 0.91 to 1.04).  No I^2^ reported |
| C2 | Bjelakovic, 2014 [146] | N=18 trials, N=50,623 [2,3,7,15,20,21,26,62,73,123,145,147–153] | Cochrane review on RCT comparing vitD at any dose, duration, and route of administration versus placebo or no intervention in adults | Incident cancers:  Overall (N=18 RCT): RR 1.00 (95% CI, 0.94 to 1.06; P = 0.88; I² =0%)  Trials on VitD3 (N=14 RCT): RR 1.00 (95% CI, 0.94 to 1.06; P = 0.88; I² = 0%)  Cancer mortality:  Trials with VitD3 (N=4 RCT): RR 0.88 (95% CI 0.78 to 0.98; P = 0.02; I² = 0%)  No effects in trials including participants with mean 25OHD levels < 50 nmol/L compared to trials including participants with mean 25OHD levels > 50 nmol/L.  No effects of concomitant calcium supplementation compared to no calcium supplementation |
| C3 | Sperati, 2013 [154] | Only breast cancer  2 RCT with N=5372 postmenopausal women): [15,145] | Included RCTs if VitD was administered as a single agent compared with placebo/no treatment or as part of combined regimens including supplements and lifestyle modifications as long as the administration of the co-intervention was planned to be the same in all groups. | RR 1.11 (95% CI, 0.74 to 1.68, I^2^=0% ) |
| C4 | Bolland, 2014 [30] | 7 RCT with N=48,167 participants [2,3,7,14,15,18,151] | MA published since January, 2009, reporting data from RCT on effects of VitD supplementation on risk of cancers. Three MA were identified [31,32,155] reporting data from 7 RCT. A new MA was performed on data from the 7 RCT.  Studies were excluded if they were cluster randomized or investigated effects of hydroxylated VitD or VitD analogues. The MA also excluded trials that included other interventions only in the VitD group, trials of fortified dairy products, and trials in populations with chronic comorbidity other than osteoporosis or frailty | Vit D alone: RR 0.98 (95% CI, 0.83 to 1.17; I^2^=10%)  CaD; RR 0.89 (95% CI, 0.67 to 1.18; I^2^=67%)  VitD with or without Ca: RR 0.99 (95% CI, 0.93 to 1.05; I^2^=0%); |
| C5 | Keum, 2014  [156] | Cancer incidence: 4 RCTs with N=45,151 participants [2,15,18,145]  Cancer mortality: 3 RCTs with N=44,260 participants [2,18,145] | Included RCT providing information on the effect of vitD supplementation (with or without Ca) on total cancer incidence or mortality. | Cancer incidence: RR 1.00 (95% CI, 0.94 to 1.06) I^2^ 0%.  Mortality: RR 0.88 (95% CI 0.78 to 0.98) I^2^ 0%. |

## Table Fb. RCTs on risk of malignant diseases

Characteristics of randomized clinical trials included in MA (Table 7A) on effects of vitamin D supplementation on risk of malignant diseases.

| **Characteristics of RCTs included in MAs on effects of vitamin D supplementation on risk of malignant diseases** | | | | | | | | | |
| --- | --- | --- | --- | --- | --- | --- | --- | --- | --- |
| **Reference, publication year, country** | | **Included in systematic reviews** | | | | | **Population**  No. of randomized (% females), Characteristics of study | **Intervention** | **Results** |
|  |  | C1 | C2 | C3 | C4 | C5 |  |  |  |
| Ott, 1989 [20], USA | |  | X |  |  |  | N=86 postmenopausal women with vertebral compression fractures.  IC-25OHD $<$50 nmol/L: No  B-25OHD: 66 nmol/L  Δ 25OHD > 50%: NR | 2 yr of treatment (DB)  Calcitriol, mean dose 0.43 µg/d.  Vs.  Placebo  Dose and dietary calcium were adjusted for hypercalciuria or hypercalcemia. | No effect  RR 3.00 (95% CI, 0.13 to 71.65) - data as reported by [146] |
| Grady, 1991 [147], USA | |  | X | X |  |  | N=98 (544% F) volunteers aged > 69 yr.  IC-25OHD $<$50 nmol/L: No  B-25OHD: ≈63±35nmol/L  Δ 25OHD > 50%: NR | 6 mo of treatment (DB):  Calcitriol 0.5 μg/d  Vs.  Placebo | No effect  RR 2.88 (95% CI, 0.12 to 69.07) - data as reported by [146] |
| Komulainen, 1999 [7], Finland | |  | X |  | X |  | N=227 postmenopausal women aged 47-56 yr.  IC-25OHD $<$50 nmol/L: No  B-25OHD: ≈28 nmol/L  Δ 25OHD > 50%: No  (data on 25OHD reported in a subgroup - reference [157]) | 5 yr of treatment (DB):  D3 300 IU/d  Vs.  Placebo | No effect  RR 0.67 (95% CI, 0.11 to 3.92) - data as reported by [146] |
| Gallagher, 2001 [21], USA | |  | X | X |  |  | N=246 women with normal age-adjusted bone mineral density, aged 65–77 yr.  IC-25OHD $<$50 nmol/L: No  B-25OHD: ≈80±27 nmol/L  Δ 25OHD > 50%: No | 3 yr of treatment (DB):  Calcitriol 0.5 μg/d  Vs.  Placebo | No effect  HR 1.20 (95% CI, 0.38 to 3.83) |
| Trivedi, 2003 [2], UK | | X | X |  | X | X | N=2,686 (24% F) community-dwelling aged 65­85 yr.  IC-25OHD $<$50 nmol/L: No  B-25OHD: ≈ NR  Δ 25OHD > 50%: NR | 5 yr. of treatment (DB):  D3 100 000 IU/ 4^th^ mo.  Vs  Placebo / 4^th^ mo., | No effect  Any incident cancer: RR 1.09 (95% CI, 0.86 to 1.36)  Colon cancer: RR 1.02 (95% CI, 0.60 to 1.74)  Respiratory cancer: RR 1.12 (95% CI, 0.56 to 2.25)  Breast cancer: RR 0.99 (95% CI, 0.25 to 3.99)  Cancer related death: RR = 0.86 (95% CI, 0.61 to 1.20) |
| Grant, 2005 [14], UK | |  |  | X | X |  | N=5292 (85% women) aged > 70 yr with a previous low-trauma fracture.  IC-25OHD $<$50 nmol/L: No  B-25OHD: 38±16 nmol/L (subgroup)  Δ 25OHD > 50%: Yes (subgroup after one year of treatment) | Mean 45 (range 24-62) mo. of treatment (DB):  D3 800 IU/d  Vs.  Ca 1000 mg/d  Vs.  D3 800 IU/d + Ca 1000 mg/d  Vs.  Placebo. | No effect  Rate of incident cancers did not differ between the VitD group (6.6%) and the control group (6.7%), as reported by [30]. |
| *Data from WHI, USA:* | | X | X |  | X | X | N=36,282 postmenopausal women  IC-25OHD $<$50 nmol/L: No  B-25OHD: NR  Δ 25OHD > 50%: NR | 7 years of treatment (DB):  D3 400 IU + Ca 1000 mg/d  Vs.  Placebo | No effect |
|  | Wactawski-Wende, 2006 [18] |  |  |  |  |  |  |  | Incident *colorectal* cancer: HR 1.08 (95 CI, 0.86 to 1.34)  Any cancer: HR 0.98 (95% CI, 0.91 to 1.05)  Risk of death from cancer: HR 0.89 (95% CI, 0.77 to 1.03) |
|  | Chlebowski, 2008 [158] |  |  |  |  |  |  |  | Incident invasive *breast* cancer:  HR 0.96 (95% CI, 0.85 to 1.09).  Death from breast cancer: HR 0.99 (95% CI, 0.55 to 1.76) |
|  | Brunner, 2011 [152] |  |  |  |  |  |  |  | Incident invasive cancers: HR 0.98 (95% CI, 0.90 to 1.05)  Cancer mortality: HR 0.90 (95% CI, 0.77 to 1.05)  No effects on specific types of cancers e.g. breast cancer (HR 0.96; 95% CI, 0.85 to 1.09) or colon cancer (HR 0.98; 95% CI, 0.76 to 1.27) |
| Lappe, 2007 [15], USA | | X | X | X | X | X | N=1,179 community-dwelling women aged > 55 yr  IC-25OHD $<$50 nmol/L: No  B-25OHD: ≈72±21nmol/L  Δ 25OHD > 50%: No | 4 yr of treatment (DB):  CaD: D3 1100 IU + Ca 1500 mg/d  Vs.  Ca-only: 1500 mg/d  Vs  Placebo | Published results (CaD group vs. placebo group): RR 0.40 (95% CI, 0.20 to 0.82)  Recalculated by Autier [159]: CaD group vs. the combined Ca-only + placebo group RR 0.59 (95% CI, 0.32 to 1.10) |
| Bolton-Smith, 2007 [148], UK | |  | X |  |  |  | N=123 healthy non-osteoporotic women aged ≥ 60 yr.  IC-25OHD <50 nmol/L: No  B-25OHD: 60 nmol/L  Δ 25OHD > 50%: No | 2 yr of treatment (DB):  D3 400 IU/d + Ca 1000 mg/d  VS  Placebo | No effect  RR 2.95 (95% CI, 0.12 to 71.09) – data as reported by [146] |
| Prince, 2008 [3], AU | |  | X |  | X |  | 302 community-dwelling ambulant women with a history of falling in the past 12 months, aged 70-90 yr.  Mean age ≈77±5 yr.  IC-25OHD $<$50 nmol/L: No  B-25OHD: ≈45±12 nmol/L  Δ 25OHD > 50%: No | 12 mo. of treatment (DB):  D2 1000 IU + Ca 1000 mg/d  Vs  Placebo + Ca 1000 mg/d | No effect  RR 0.20 (95% CI, 0.02 to 1.69) ) – data as reported by [146] |
| Daly, 2008 [149], AU | |  | X |  |  |  | N=167 community-dwelling healthy men aged >50 yr.  IC-25OHD $<$50 nmol/L: No  B-25OHD: 77 ± 23 nmol/L  Δ 25OHD > 50%: No  (25OHD levels reported in reference [160]) | 2 yr. of treatment (NB):  D3 800 IU/d + Ca 1000 mg/d provided as fortified milk  Vs.  No treatment | No effects on incident cancers at follow-up 18 mo. after end of trial:  RR 1.29 (95% CI, 0.30 to 5.57) – data as reported by [146] |
| Janssen, 2010 [150], The Netherlands | |  | X |  |  |  | N=70 female geriatric patients aged >65 yr.  IC-25OHD $<$50 nmol/L: Yes  B-25OHD: ≈35±11 nmol/L  Δ 25OHD > 50%: Yes | 6 mo. of treatment (DB):  D3 400 IU/d  Vs.  Placebo  All participants also received Ca 500 mg/d | No effect  RR 0.32 (95% CI, 0.01 to 7.48) – data as reported by [146] |
| Sanders, 2010 [151], AU | |  | X |  | X |  | N=2258 community-dwelling women aged > 70 yr and at risk of fracture  IC-25OHD $<$50 nmol/L: No  B-25OHD 49 nmol/L (sub-study)  Δ 25OHD > 50%: No (sub-study) | 3–5 yr of treatment (DB):  D3 500,000 IU/yr  Vs.  Placebo | No effect  RR 0.70 (95% CI, 0.27 to 1.83) |
| Avenell, 2012 [145], UK | | X | X | X |  | X | N=5292 (85% women) aged > 70 yr. with a previous low-trauma fracture.  IC-25OHD $<$50 nmol/L: No  B-25OHD: 38±16 nmol/L (subgroup)  Δ 25OHD > 50%: Yes (subgroup after one year of treatment) | Mean 45 (range 24-62) mo of treatment (DB):  D3 800 IU/d  Vs.  Ca 1000 mg/d  Vs.  D3 800 IU/d + Ca 1000 mg/d  Vs.  Placebo. | No effect  Same study as reported by Grant et al [14] but longer follow-up (6.2 years after start of study):  VitD vs. no-VitD:  Cancer incidence: HR 1.07 (95% CI, 0.92 to1.25)  Cancer mortality: HR 0.85 (95% CI, 0.68 to 1.06)  Adjustment for compliance did not change results. |
| Larsen, 2012 [62], Denmark | |  | X |  |  |  | N=112 (69% F) hypertensive patients, aged 61±10 yr.  IC-25OHD $<$50 nmol/L: No  B-25OHD: 58±25 nmol/L  Δ 25OHD > 50%: Yes | 20 weeks of treatment (DB):  D3 3000 IU/d  Vs.  Placebo | No effect  Cancer incidence: RR 3.00 (95% CI, 0.12 to 72.31) – data as reported by [146] |
| Wood, 2012 [73], UK | |  | X |  |  |  | N=265 community-dwelling post-menopausal women aged 64±2 yr.  IC-25OHD $<$50 nmol/L: No  B-25OHD: ≈33±15 nmol/L  Δ 25OHD > 50%: Yes  Excluded women with known CVD or diabetes. | 12 mo. of treatment (DB):  D3 400 IU/d  Vs.  D3 1000 IU/d  Vs.  Placebo | No effect  RR 1.00 (95% CI, 0.09 to 10.95) |
| Murdoch, 2012 [153], NZ | |  | X |  |  |  | N=322 (75% F) healthy adults aged > 18 yr.  IC-25OHD < 50 nmol/L: No  B-25OHD: 72±22 nmol/L  Δ 25OHD > 50%: Yes | 18 mo. of treatment (DB):  D3: initial dose of 200 000 IU, then 200 000 IU 1 mo. later, then 100 000 IU monthly  Vs.  placebo (identical dosing regimen) | No effect  RR 4.00 (95% CI, 0.45 to 35.40) – data as reported by [146] |
| Glendenning, 2012 [123], AU | |  | X |  |  |  | N=686 women living independently aged > 70 yr.  IC-25OHD < 50 nmol/L: No  B-25OHD: 66±23 nmol/L (sub-group)  Δ 25OHD > 50%: No | At baseline and after 3 and 6 mo. of treatment (DB):  D3 150,000 IU  Vs  Placebo | No effect  9 mo. after start of treatment: RR 1.19 (95% CI, 0.62 to 2.31) |
| Witham, 2013 [26], Scotland | |  | X |  |  |  | N=159 (59% F) with isolated systolic hypertension aged >70 yr.  IC-25OHD < 50 nmol/L: No  B-25OHD: 45±15 nmol/L  Δ 25OHD > 50%: Yes | Every 3 months for 1 year (DB):  D3 100,000 IU  Vs  Placebo | No effect: RR 0.99 (95% CI, 0.14 to 6.84) – data as reported by [146] |

## Table Ga. Systematic reviews on respiratory tract infections

SR with MA reporting summary data on findings from RCT on effects of vitamin D supplementation on risk of respiratory tract infections (RTI).

| **Systematic reviews on effects of vitamin D supplementation on risk of respiratory tract infections** | | | | |
| --- | --- | --- | --- | --- |
| **MA#** | **Reference, publication year** | **Papers included** | **Criteria for inclusion of studies** | **Results** |
| I1 | Charan, 2012 [161] | 5 RCT with N=1417 participants [162–166] | A SR and MA of RCTs published in English from 1966 to 2011 that studied effects of VitD supplementation for treatment or prevention of infectious diseases.  Included only RCTs exploring the effect of VitD supplementation on prevention of RTI like influenza, pneumonia, common cold, etc. Only clinical trials where results were given in categorical variables were included. Excluded trials in which VitD was used in combination with other interventions. | VitD supplementation significantly reduced the incidence of RTI: OR = 0.58; 95% CI, 0.42 to 0.81, I^2^ = 6%  Sub-group analyses, including only children (N=2 RCT): OR 0.58 (95% CI, 0.42 to 0.81, I^2^ = 0%). |
| I2 | Bergman, 2013 [167] | 11 RCT with N=5660 participants [153,162–166,168–172] | A systematic search using electronic databases on RCTs (published through April 15, 2013) reporting incident RTI. No distinction was made between “upper” and “lower” RTI.  Excluded studies addressing tuberculosis or fungal infections. | VitD showed a protective effect against RTI: OR 0.64; 95% CI, 0.49 to 0.84, I^2^ = 72%.  Interaction analyses (p<0.01) showed differences on the protective effect depending on whether VitD was administered once-daily dosing (OR 0.51; 95% CI, 0.39 to 0.67) or as a bolus dose (OR 0.86; 95% CI, 0.62 to 1.20).  No other trial-level parameters investigated were significant predictors of VitD effectiveness, including if the endpoint was primary or secondary (p = 0.35), if healthy individuals or patients were studied (p = 0.24) as well as age (p = 0.91), gender (p = 0.61), dose (p = 0.3), trial duration (p = 0.89), baseline 25OHD levels (p = 0.43) or latitude (p = 0.27). |
| I3 | Mao, 2013 [173] | 7 RCT with N=4827 participants [153,163–165,169–171] | A systematic search was performed of the major electronic databases through September 2013 on RCT comparing treatment with VitD3 with placebo on risk of RTI in healthy subjects. No language restriction was imposed. Included only studies reporting RR with 95% CI (or data to calculate them) Excluded low-quality studies in terms of a modified Jadad score ≤ 3 [174]. | No effects: RR 0.98; 95% CI, 0.93 to 1.03, I^2^ = 35%.  Meta-regression analyses showed almost no impact on the RR of age, vitamin D dosing regimen, and length of follow-up. Sensitivity analyses showed little impact on the pooled risk estimates by omission of any single trial. |
| I4 | Xiao, 2015 [175] | 4 RCTs on RTI with N=5,593 participants  [165,168,170,171] | Only RCTs comparing vitamin D supplementation with either placebo or no intervention in children younger than 18 years of age. | No effects  RTI: RR 0.79; 95% CI, 0.55 to 1.13, I^2^ 74%  Risk of pneumonia: RR 1.06, 95% CI, 0.90 to 1.25, I^2^ 0%  All-cause mortality: RR 1.18 (95% CI, 0.71 to 1.94)  Rate of hospital admission due to RTI in healthy children: RR 0.95 (95% CI, 0.72 to 1.26).  In children previously diagnosed with asthma, vitamin D supplementation reduced risk of asthma exacerbation (RR 0.26, 95% CI, 0.11 to 0.59; I^2^ 0.0%). |
| I5 | Yakoob et al, 2016 [176] | 2 RCTs with N=3134 participants  [171,177] | - Only children < 5 yr. of age  - Co-interventions should be identical in both groups i.e., vitamin D supplementation was the only difference between the intervention and control groups.  - excluded studies that evaluated the effects  of food fortiﬁcation or vitamin D-rich foods | No effects  Radiologically confirmed pneumonia - 2 RCTs (3134 participants): RR 1.06 (95% CI, 0.89 to 1.26)  Confirmed or unconfirmed pneumonia: 1 RCT (3046  participants): RR 0.95 (95% CI 0.87 to 1.04)  BUT increase risk of repeated episodes of pneumonia confirmed by chest radiograph 1 RCT (3046  participants): RR 1.69 (95% CI 1.28 to 2.21) |
| I6 | Vuichard Gysin, 2016 [178] | 15 RCTs with N=7,053 participants  [153,162–165,170,171,179–186] | Included RCTs comparing VitD vs. placebo or no treatment in previously healthy individuals on  a) the prevention of RTIs  b) the duration of RTIs  c) severity of RTIs | No significant effect on   - clinical RTIs: RR 0.94; 95% CI, 0.88 to 1.00, I^2^ 57% - Symptom duration (6 RCTs): MD -0.06; 95% CI -0.29 to 0.18 days - Severity of RTI (5 RCTs): OR 0.95; 95% CI 0.76 to 1.18   No significant interactions comparing:   - 25OHD below vs. above 50 nmol/L at baseline: P_i_=0.08 - Daily/weekly vs. les frequent: P_i_ =0.06 |
| I7 | Martineau, 2017 [187] | 25 RCT with N=11,321 participants  [25,153,163–166,168,170–172,179–186,188–195].  IPD analysis included data from N=10,933 (96.6%) of the participants. | Included double-blind RCTs on supplementation with vitamin D3 or vitamin D2 of any duration if data on incidence of acute RTI were collected prospectively and pre-specified as an efficacy outcome. | Acute RTI: OR 0.88, 95% CI, 0.81 to 0.96 (IPD meta-analysis).  *Subgroup analysis:*  Protective effects were seen in those receiving daily or weekly vitamin D without additional bolus doses (adjusted odds ratio 0.81, 0.72 to 0.91), but not in those receiving one or more bolus doses (adjusted OR 0.97, 95% CI, 0.86 to 1.10; P_i_ =0.05).  Among those receiving daily or weekly vitamin D, protective effects were stronger in those with baseline 25OHD <25 nmol/L (adjusted OR 0.30, 95% CI, 0.17 to 0.53) than in those with baseline 25OHD ≥25 nmol/L (adjusted OR 0.75, 95% CI, 0.60 to 0.95; P_i_ =0.006). |

## **Table Gb. RCTs on risk of respiratory tract infections**.

Characteristics of randomized clinical trials included in MA (Table 8A) on effects of vitamin D supplementation on risk of respiratory tract infections (RTI).

| **Characteristics of the RCTs included in MAs on effects of vitamin D supplementation on risk of respiratory tract infections** | | | | | | | | | | |
| --- | --- | --- | --- | --- | --- | --- | --- | --- | --- | --- |
| **Reference, publication year, country** | **Included in MA** | | | | | | | **Population**  No. of randomized (% females), Characteristics of study | **Intervention** | **Results** |
|  | I1 | I2 | I3 | I4 | I5 | I6 | I7 |  |  |  |
| Aloia, 2007 [162], USA | X | X |  |  |  | X |  | 208 African-American post-menopausal women.  IC-25OHD <50 nmol/L: No  B-25OHD: NR  Δ 25OHD > 50%: NR | 3 yr. of therapy (DB):  D3 800 IU/d → after 2^nd^ yr.: 2000 IU/d  Vs.  Placebo | Risk of infections was assessed by review of adverse events (not primary endpoint)  After 3 yr.: cold and influenza symptoms were reported by 8 in the D3-group and by 26 in the placebo group: OR 0.25 (95% CI, 0.12 to 0.42, P<0.01). |
| Li-Ng, 2009 [163], USA | X | X | X |  |  | X | X | N=162 (59 % F) healthy adults  Mean age: 58±14 yr.  IC-25OHD <50 nmol/L: No  B-25OHD: 64±26 nmol/L  Δ 25OHD > 50%: No | 12 weeks of therapy (DB):  D3 2000 IU/d  Vs.  Placebo | Primary endpoint: prevention of upper RTI:  No difference in the incidence of URIs between the vitD and placebo groups: RR 0.88 (95% CI, 0.58 to 1.35).  There was no difference in the duration or severity of URI symptoms between the vitamin D and placebo groups [5.4±4.8 days vs. 5.3±3.1 days, respectively (p=0.86). |
| Laaksi, 2010 [164], Finland | X | X | X |  |  | X | X | 164 voluntary young Finnish men aged 18–28 years undergoing compulsory periodic military training  IC-25OHD <50 nmol/L: No  B-25OHD: 76±19 nmol/L  Δ 25OHD > 50%: No | 6 mo. of therapy (DB):  D3 400 IU/d  Vs.  Placebo | Primary endpoint: number of days absent from duty because of acute RTI  Mean number of days absent: 2.2±3.2 days in the intervention group and 3.0±4.0 days in the placebo group (P=0.096).  Risk of RTI: OR 0.57 (95% CI, 0.30 to 1.07) as reported by [161] |
| Urashima, 2010 [165], Japan | X | X | X | X |  | X | X | 430 (44% F) schoolchildren aged 6–15 yr.  IC-25OHD <50 nmol/L: No  B-25OHD: NR  Δ 25OHD > 50%: NR | 4 mo. of therapy (DB):  D3 1200 IU/d  Vs  Placebo | Primary outcome: incidence of influenza A  RR 0.58 (95% CI, 0.34 to 0.99) |
| Manaseki-Holland, 2010 [166], Afghanistan | X | X |  |  |  |  | X | N=453 (43% F) children aged 1–36 mo, living I Kabul and diagnosed with non-severe or severe pneumonia at an outpatient clinic.  IC-25OHD <50 nmol/L: No  B-25OHD: NR  Δ 25OHD > 50%: NR | A single oral dose – with 3 mo. of follow-up:  D3 100,000 IU  Vs  Placebo  All were also treated with antibiotics. | Primary endpoints: whether VitD reduces the duration of illness in children with pneumonia and risk of repeat episodes.  No difference in mean number of days to recovery between the VitD (4.74±2.22) and the placebo arm (4.98±2.89; P = 0.17).  The risk of a repeat episode of pneumonia within 90 days was lower in the intervention (92 ⁄ 204; 45%) than the placebo group [122 ⁄ 211; (58%). RR 0.78 (95% CI, 0.64 to 0.94; P = 0.01]. |
| Alonso, 2011 [177], Spain |  |  |  |  | X |  |  | N=88 full-term 1-month-old healthy infants  IC-25OHD <50 nmol/L: No  B-25OHD: NR  Δ 25OHD > 50%: NR | 1 yr. of therapy (DB):  D3 402 IU/d  Vs.  Placebo | Infections were not a primary endpoint  No effects on infectious diseases |
| Majak, 2011 [168], Poland |  | X |  | X |  |  | X | N=48 (33% F) children aged 5-18 yr. with newly diagnosed asthma.  IC-25OHD <50 nmol/L: No  B-25OHD: 89±38 nmol/L  Δ 25OHD > 50%: No | 6 mo. of therapy (DB):  Inhaled budesonide with D3 500 IU/d  Vs  Inhaled budesonide without D3 | Outcomes: effects of VitD on symptom score, lung function, and the number of exacerbations  VitD reduced risk of exacerbations: RR 0.36 (95% CI, 0.13 to 0.98)  In children with a decrease of 25OHD levels, the risk of asthma exacerbation was 8 times higher than in children with a stable or increased 25OHD level (OR 8.6; 95% CI, 2.1 to 34.6). |
| Kumar, 2011 [188], India |  |  |  |  |  |  | X | N=2079 (47% F) Low birthweight infants born at term  IC-25OHD <50 nmol/L: No  B-25OHD: NR  Δ 25OHD > 50%: NR | 6-month of therapy (DB):  D3 1400 IU/wk  Vs  Placebo | RTI was secondary outcome  No effect on risk of infections. |
| Jorde, 2011 [169], Norway |  | X | X |  |  |  |  | N=549 (43% F) from 10 different RCTs were included  IC-25OHD <50 nmol/L: No  B-25OHD: NR  Δ 25OHD > 50%: NR | At least 12 weeks of therapy (DB):  D3: 1111–6800 IU/d  Vs  Placebo | Primary outcome: different in the 10 included RCTs.  VitD did not reduce risk of Influenza-like disease:  RR 0.88 (95% CI, 0.58 to 1.32). |
| de Gruijl, 2012 [179], The Netherlands |  |  |  |  |  | X |  | N= 105 (91% F) young adults aged 18–30 yr  IC-25OHD <50 nmol/L: No  B-25OHD: ≈ 60 nmol/L  Δ 25OHD > 50%: Yes | 8 weeks of therapy (NB):  D3 1000 IU/d  Vs  3 times a week sub-sunburn sunbed exposure  Vs  No treatment | RTI was primary outcome  No effect on number of self-reported colds |
| Choudhary, 2012 [196], India |  |  |  |  |  |  |  | N=200 (40% F) children aged 2 mo. to 5 yr. with severe pneumonia.  Mean age: 14±12 mo.  IC-25OHD <50 nmol/L: No  B-25OHD: NR  Δ 25OHD > 50%: NR | 5 days of therapy (DB):  D3 1000 IU/d if age < 1 yr; 2000 IU/d if age > 1 yr  Vs  Placebo | Primary outcome was time to resolution of severe pneumonia.  No effect of vitamin D3 on treatment responses. |
| Camargo, 2012 [170], Mongolia |  | X | X | X |  | X | X | N=244 (59% F) Mongolian schoolchildren aged ≈10±1 yr.  IC-25OHD <50 nmol/L: No  B-25OHD: 19 nmol/L  Δ 25OHD > 50%: Yes | 7 weeks with intake of (DB):  D3 fortified milk 300 IU/d  Vs.  Unfortified milk | Primary outcome: the number of parent-reported RTIs during 3 mo. of follow-up  Incidence rate ratio: 0.52 (95% CI, 0.31 to 0.89)  Similar results were found among children either below or above the median 25OHD level at baseline |
| Lehouck, 2012 [25], Belgium |  |  |  |  |  |  | X | N= 182 (20% F) patients with moderate to very severe COPD and a history of recent exacerbations  IC-25OHD <50 nmol/L: No  B-25OHD: ≈ 50 nmol/L  Δ 25OHD > 50%: Yes | 1 yr. of therapy (DB):  D3 100,000 IU every 4 wk  Vs.  Placebo | Primary outcome: time to first exacerbation.  Overall, no effect on median time to first exacerbation  *Post-hoc analysis*: if baseline 25OHD < 25 nmol/L, treatment caused a significant reduction in exacerbations (rate ratio, 0.57; 95% CI, 0.33 to 0.98). |
| Manaseki-Holland, 2012 [171], Afghanistan |  | X | X | X | X | X | X | N=3046 (48% F) children aged 1–11 months in Kabul  IC-25OHD <50 nmol/L: No  B-25OHD: NR  Δ 25OHD > 50%: yes | 18 mo. of follow up:    D100,000 IU D3/quarterly  Vs  Placebo/quarterly | Primary endpoint: first episode of radiologically confirmed pneumonia  No effect of vitamin D: Incidence rate ratio 1.06 (95% CI, 0.89 to 1.27).  2/652 children had 25OHD levels greater than 375 nmol/L in the intervention group, which was considered a toxic level. |
| Murdoch, 2012 [153], NZ |  | X | X |  |  | X | X | N=322 (75% F) healthy adults, aged > 18 yr.  IC-25OHD <50 nmol/L: No  B-25OHD: 72±22 nmol/L  Δ 25OHD > 50%: Yes | 18 mo. of therapy (DB):  D3: initial dose of 200,000 IU, then 200 000 IU 1 mo. later, then 100 000 IU monthly  Vs.  Placebo (identical dosing regimen) | Primary endpoint: upper RTI.  No effect of vitamin D: RR 0.97; 95% CI, 0.85 to 1.11  No effect on number of days of missed work (RR 1.03; 95% CI, 0.81 to 1.30) or duration of symptoms (RR 0.96; 95% CI, 0.73 to 1.25).  Findings remained unchanged when analyses were repeated by season and by baseline 25-OHD levels |
| Bergman, 2012 [172], Sweden |  | X |  |  |  |  | X | N=140 (73% F) patients with antibody deficiency or increased susceptibility to RTIs  IC-25OHD <50 nmol/L: No  B-25OHD: ≈55 nmol/L  Δ 25OHD > 50%: yes | 12 mo. therapy (DB):  D3 4000 IU/d  Vs.  Placebo | Primary endpoint was an infectious score based on five parameters: symptoms from respiratory tract, ears and sinuses, malaise and antibiotic consumption.  Adjusted relative score: 0.77 (95% CI, 0.60 to 0.99, p=0.04) |
| Marchisio, 2013 [189], Italy |  |  |  |  |  |  | X | N= 116 (45% F) children aged 1-5 yr. with a history of recurrent acute otitis media  Mean age: 34±12 mo.  IC-25OHD <50 nmol/L: No  B-25OHD: ≈68 nmol/L  Δ 25OHD > 50%: No | 4 mo. of therapy (DB):  D3 1000 IU/d  Vs.  Placebo | Co-primary outcomes: total number of acute otitis media episodes, and the number of complicated and uncomplicated episodes.  Number of children experiencing ≥1 episode of acute otitis media was significantly lower in the treatment group (26 versus 38; P = 0.03). There was a significant difference in the number of children who developed uncomplicated acute otitis media (P < 0.001). |
| Rees, 2013 [180], USA |  |  |  |  |  | X | X | N=759 (42% F) participants aged 45–75 yr., in good health, and with a history of colorectal adenoma, and 25OHD ≥ 37.5 nmol/L.  Mean age: 58±7 yr.  IC-25OHD <50 nmol/L: No  B-25OHD: ≈63 nmol/L  Δ 25OHD > 50%: No | 13 mo. (average) of therapy (DB):  D3 1000 IU/d  Vs.  Ca 1200 mg/d  Vs.  D3 1000 IU/d + Ca 1200 mg/d  Vs.  Placebo | Upper respiratory tract infection was not a primary end-point.  Treatment did not affect incidence or duration of upper respiratory tract infection |
| Tran, 2014 [181], AU |  |  |  |  |  | X | X | N= 644 (47% F) elderly (aged 60–84 y) from the general community.  Mean age: 72 yr.  IC-25OHD <50 nmol/L: No  B-25OHD: ≈42 nmol/L  Δ 25OHD > 50%: Yes | Up to 12 mo. of therapy (DB):  D3 30,000 IU/mo.  Vs.  D3 60,000 IU/mo.  Vs  Placebo | Infections was not a primary end-point.  No significant effect on number of antibiotic prescription |
| Goodall, 2014 [182], CA |  |  |  |  |  | X | X | N= 600 (64% F) healthy university students aged ≥17 yr. and living with at least one student housemate.  Median age: 19 yr.  IC-25OHD <50 nmol/L: No  B-25OHD: NR  Δ 25OHD > 50%: NR | 8 weeks of therapy (DB):  D3 10,000 IU/wk. plus gargling  Vs.  D3 10,000 IU/wk. without gargling  Vs.  Placebo and gargling  Vs.  Placebo and no gargling. | Upper respiratory tract infection was primary end-point  No significant effect on self-reported clinical upper respiratory tract infection.  D3 was associated with a significantly lower risk for laboratory confirmed URTI (RR: 0.54, 95% CI, 0.34 to 0.84, p=0.007) and with a significantly lower mean viral load (mean difference: −0.89, 95% CI, -1.7 to −0.06, p = 0.04). |
| Urashima, 2014 [183], Japan |  |  |  |  |  | X | X | N=247 (34% F) high school students.  Mean age: 16±1 yr.  IC-25OHD <50 nmol/L: No  B-25OHD: NR  Δ 25OHD > 50%: NR | 2 mo. of therapy (DB):  D3 2000 IU/d  Vs  Placebo | Primary outcome: incidence of influenza A diagnosed by a rapid influenza diagnostic test.  No effect of D3 on the overall incidence of influenza A |
| Dubnov-Raz, 2015 [184], Israel |  |  |  |  |  | X | X | N=55 (36% F) competitive adolescent swimmers with vitamin D insufficiency  Mean age: 15±2 yr.  IC-25OHD <50 nmol/L: Yes  B-25OHD: 24±5 nmol/L  Δ 25OHD > 50%: Yes | 12 weeks during winter time:  D3 2000 IU/d  Vs  Placebo | Primary end-point: risk of upper respiratory infection (URI).  No effect on frequency, severity, or duration of upper respiratory infection. |
| Grant, 2015 [185], NZ |  |  |  |  |  | X | X | N=260 pregnant women  and offspring  Mean age: NR.  IC-25OHD <50 nmol/L: No  B-25OHD: 55±26 nmol/L  Δ 25OHD > 50%: NR | 3 mo. in pregnancy + 6 mo. in infancy:  Woman/infant pairs were randomized to:  D3 1000 IU/400 IU/d  Vs.  D3 2000 IU/800 IU/d  Vs.  Placebo/placebo | Primary outcome: primary care visits for acute respiratory infection (ARI).  Compared with placebo the proportion of children making any ARI visits was smaller in the higher-dose (87%, p = 0.004), but not the lower-dose vitamin D3 group (95%, p = 0.17). |
| Martineau, 2015 [190], UK |  |  |  |  |  |  | X | N=240 (40% F) adults with COPD  Mean age: 65±9 yr.  IC-25OHD <50 nmol/L: No  B-25OHD: 46±26 nmol/L  Δ 25OHD > 50%: NR | 1 yr. of therapy (DB):  D3 120,000 IU every 2^nd^ mo.  Vs  Placebo | Primary endpoint: moderate or severe COPD exacerbations and upper respiratory infections  No effect on time to first moderate or severe exacerbation or time to first upper respiratory infection. |
| Martineau, 2015 [191], UK |  |  |  |  |  |  | X | N=250 (56% F) adults with asthma  Mean age: 48±14 yr.  IC-25OHD <50 nmol/L: No  B-25OHD: 50±25 nmol/L  Δ 25OHD > 50%: No | 1 yr. of therapy (DB):  D3 120,000 IU every 2nd mo.  Vs  Placebo | Co-primary outcomes: asthma exacerbation and upper respiratory infections  No effect on time to first severe exacerbation or time to first upper respiratory infection. |
| Martineau, 2015 [192], UK |  |  |  |  |  |  | X | N=240 (66% F) older adults and their carers  Mean age: 67±13 yr.  IC-25OHD <50 nmol/L: No  B-25OHD: 43±23 nmol/L  Δ 25OHD > 50%: No | 1 yr. of therapy (DB):  D3:   - Elderly: 96,000 IU every 2^nd^ mo. + 400 IU/d - Carers: 120,000 IU every 2^nd^ mo.   Vs   - Elderly: placebo every 2^nd^ mo. + D3 400 IU/d - Carers: placebo every 2^nd^ mo. | Primary outcome was time to first acute respiratory infection  No effect on time to first acute respiratory infection  D3 was associated with increased risk of upper respiratory infection and increased duration of symptoms |
| Simpson, 2015 [186], AU |  |  |  |  |  | X | X | N= 34 (59% F) healthy adults.  Mean age: 32±12 yr.  IC-25OHD <50 nmol/L: No  B-25OHD: 68±23 nmol/L  Δ 25OHD > 50%: No | 17 weeks of therapy (DB):  D3 20,000 IU/week  Vs  Placebo | Primary endpoint: occurrence of acute infection  No effect on risk of infection, duration or severity |
| Denlinger, 2016 [193], USA |  |  |  |  |  |  | X | N=408 adult with asthma and 25OHD <75 nmol/L  Mean age: 39±13 yr.  IC-25OHD <50 nmol/L: No  B-25OHD: 47±17 nmol/L  Δ 25OHD > 50%: Yes | 28 weeks of therapy (DB):  D3 single dose of 100,000 IU plus 4,000 IU/d  Vs.  Placebo | Prospectively planned secondary analysis with cold symptom severity assessed with symptom score as outcome  D3 had no effect on cold symptom severity |
| Tachimoto, 2016 [194], Japan |  |  |  |  |  |  | X | N=89 (44% F) schoolchildren with asthma  Mean age: 10±2 yr.  IC-25OHD <50 nmol/L: No  B-25OHD: 75±25 nmol/L  Δ 25OHD > 50%: No | 2 mo. of therapy (DB):  D3 800 IU/d  Vs.  Placebo | Primary outcome: frequency and severity of asthma  D3 significantly improved asthma control |
| Ginde, 2016 [195], USA |  |  |  |  |  |  | X | N=107 (58% F) older long-term care residents aged >60 yr.  Mean age: 81±10 yr.  IC-25OHD <50 nmol/L: No  B-25OHD: 57±23 nmol/L  Δ 25OHD > 50%: No | 1 yr. of therapy (DB):  D3 bolus 100,000 IU + ≤1000 IU/d  Vs.  Placebo bolus + 400-1000 IU/d | Primary outcome: incidence of acute respiratory infection.  High-dose D3 reduced the incidence of ARI: incidence rate ratio = 0.60; 95% CI, 0.38 to 0.94)  Falls were more common in the high-dose group |

## Table Ha. Systematic reviews on depression.

SR with MA reporting summary data on findings from RCT on effects of vitamin D supplementation on risk of depression.

| **Systematic reviews on effects of vitamin D supplementation on risk of depression** | | | | |
| --- | --- | --- | --- | --- |
| **#MA** | **Reference, publication year** | **Papers included** | **Criteria for inclusion of studies** | **Results** |
| D1 | Spedding et al., 2014 [197] | 2 RCT without flaws: N=483 participants [198,199]  2 RCT with flaws: N= 2,875 participants [24,200] | 15 RCT were identified on effects of VitD supplementation on depression. In MA, analyses were stratified by “biological flaws”  The authors excluded studies that combined VitD with any other vitamin, antidepressant, calcium, or light therapy.  7 studies were considered as being *without* flaws ([198,199,201–205]) – but only 2 were included in the MA ([198,199]) as they both used BDI showing beneficial effect of VItD on depression score.  8 studies were considered as being *with*  flaws ([24,200,206–211]), but only two were included in MA [24,200] | Beneficial effect of VitD in MA of studies without flaws: SMD 0.78; 95% CI, 0.24 to 1.27.  Harmful effect of VitD in MA of studies with flaws: SMD −1.1; 95% CI, −0.7 to −1.5. |
| D2 | Li et al. 2014 [212] | 6 RCT with N=1,203 participants [24,68,198,199,201,211] | In the SR, RCTs were only included if they were placebo-controlled with a parallel group design and reported effects of oral VitD as mono-intervention in subjects aged > 18 yr. and being *at risk* of depression.  The SR excluded trials with another primary focus e.g., major psychiatric condition such as anxiety disorders and studies involving participants with diseases known to affect VitD metabolism such as hyperparathyroidism. | The protocol was published prior to study results [213]  MAs showed no significant effect of VitD supplementation on post-intervention depression scores: SMD -0.14; 95% CI, -0.41 to 0.13 or risk of depression OR 0.93; 95% CI, 0.54 to 1.59.  The quality of evidence was considered low.  No significant differences were demonstrated in subgroup or sensitivity analyses. |
| D3 | Shaffer et al. 2014 [214] | 7 RCT with N=3,191 participants [68,198,199,207,209,215,216]. | Included data from RCT that compared the effect of vitD supplementation on depressive symptoms to a control condition.  The SR included studies of both non-depressed and depressed individuals  Primary endpoint: Data on mean (SD) depressive symptoms were extracted (no studies included a diagnosis of depressive disorder as end point). | No overall effect on depressive symptoms: SMD: 0.14; 95% CI, -0.33 to 0.05. High degree of heterogeneity.  Subgroup analysis:  Moderate effect for subjects with clinically significant depressive symptoms/disorder (2 RCT): SMD, -0.60; 95% CI, -1.19 to -0.01; p <0.05.  Most trials were considered to have an unclear or high risk of bias. High degree of between trial variation in the amount, frequency, duration, and mode of delivery of vitD. |
| D4 | Gowda et al, 2015 [217] | 9 RCTs with N=4923 participants  [24,68,198,199,201,202,207,209,211] | Included data from RCTs RCTs) in which vitamin D supplementation was used to reduce depression or depressive symptoms.  Included only data from studies performed in subjects aged ≥18 yr. diagnosed with depressive disorder based on both the Diagnostic and Statistical Manual of Mental Disorders or other symptom checklist for depression. | No overall effect on depression: SMD 0.28; 95% CI, −0.14 to 0.69), I^2^ = 85%  Exclusion of 1 RCT [209] reduced the heterogeneity – favoring placebo (SMD = 0.03; 95% CI, 0.02 to 0.04; P < 0.05).  Most of the studies focused on individuals with low levels of depression and adequate 25OHD levels at baseline. |

SMD: Standardized mean difference

1. Cross-over RCTs was excluded because depressive symptoms may not be static and participants’ variability is hard to interpret.

## Table Hb. RCTs on risk of depression.

Characteristics of randomized clinical trials included in MA (Table 9A) on effects of vitamin D supplementation on risk of depression.

| **Characteristics of the RCTs included in MAs on effects of vitamin D supplementation on risk of depression** | | | | | | | | |
| --- | --- | --- | --- | --- | --- | --- | --- | --- |
| **Reference, publication year, country** | **Included in MA** | | | | **Population**  No. of randomized (% females), Characteristics of study | **Intervention** | **Depression assessment** | **Results** |
|  | **D1** | **D2** | **D3** | **D4** |  |  |  |  |
| Lansdowne, 1998 [202], AU |  |  |  | X | N=44 (77% F) healthy students  Mean age 22 yr.  IC-25OHD <50 nmol/L: No  B-25OHD: NR  Δ 25OHD > 50%: NR | 5 days of therapy (DB):  D3 400 IU/d + vitamin A 9000 IU/d  Vs.  D3 800 IU/d+ vitamin A 8000 IU/d  Vs.  Vitamin A 10,000 IU/d without D3 | The Positive and Negative Affect Schedule (PANAS)  was used as a self-report measure of both positive and negative affectivity. | D3 significantly enhanced positive affect and there was some evidence of a reduction in negative affect. |
| Hogie-Lorenzen, 2003 [215], US |  |  | X |  | N≈98 (59% F) community-dwellings aged > 60 yr.  IC-25OHD <50 nmol/L: No  B-25OHD: 8.2±3.0 nmol/L  Δ 25OHD > 50%: NR | 8 wk. of treatment:  D3 Fortified cheese with 600 IU/d  Vs.  Non-fortified cheese or no cheese | GDS | No effect of the intervention - as reported by Shaffer et al [214] |
| Dumville, 2006 [200], UK | X |  |  |  | N=2117 elderly women with seasonal affective disorders aged > 70 yr.  IC-25OHD <50 nmol/L: No  B-25OHD: NR  Δ 25OHD > 50%: NR | 6 mo. of therapy (NB)  D3 800 IU/d + Ca  Vs.  None | SF-12 (MCS) | Primary outcome: Depression  No effect of D3 on MCS (−0.49; 95% CI, −1.34 to 0.81) |
| Jorde et al. 2008 [198], Norway | X | X | X | X | N= 441 (64% F) overweight or obese  Age 49±11 yr.  IC-25OHD <50 nmol/L: No  B-25OHD: 58±21 nmol/L  Δ 25OHD > 50%: Yes  None used antidepressant | 12 mo. of therapy (DB):  D3: 40,000 IU/wk.  Vs.  D3: 20,000 IU/wk.  Vs.  Placebo  All participants also received Ca 500 mg/d | BDI | Depression was *not* the primary outcome.  ITT analyses: no effect of VitD.  *Per protocol analysis*: the BDI subscale 1–13 (cognitive-affective indices) showed a decrease in depressive symptoms in the VitD group compared with the placebo group. |
| Arvold, 2009 [201], USA |  | X |  | X | N=100 (%F: NR) seeking medical care at primary healthcare clinic.  Age ≈58±15 yr.  IC-25OHD <50 nmol/L: No  B-25OHD_B_ ≈45±10 nmol/L  Δ 25OHD > 50%: Yes | 8 wk. of therapy (DB):  D3 50,000 IU/wk.  Vs  Placebo | Questionnaires about fibromyalgia  (FIQ) including depression as a domain. | Depression was *not* the primary outcome.  No effect of D3 on the sub-domain “depressed mood” |
| Sanders, 2011 [24], AU | X | X |  | X | N=2258 community-dwelling women aged > 70 yr and at risk of fracture  IC-25OHD <50 nmol/L: No  B-25OHD 49 nmol/L (sub-study)  Δ 25OHD > 50%: No (sub-study) | 3–5 yr of therapy (DB):  D3 500,000 IU/yr  Vs.  Placebo | - GHQ - SF-12 - Patient Global Impression–Improvement scale - WHO-5 | Depression was *not* a primary outcome.  No significant differences between the VitD and placebo groups were found on any of the measured outcomes of mental health.  No interaction between those on antidepressant/anxiety medication at baseline and the treatment groups. |
| Dean, 2011 [209], AU |  |  | X | X | N=128 (57% F) healthy young adults  Age 22±3 yr.  IC-25OHD <50 nmol/L: No  B-25OHD 77±20 nmol/L  Δ 25OHD > 50%: No | 6 wk. of therapy (DB):  D3 5000 IU/d  Vs  Placebo | BDI | An emotional state of depression was a co-primary end point.  Overall no effects on BDI  No effect in participants with baseline levels of 25OHD < 75 nmol/L |
| Bertone-Johnson, 2012 [207], WHI, USA |  |  | X | X | N= 2,263 (/36,282) post-menopausal women (sub-set of the WHI RCT)  Age 62±7 yr.  IC-25OHD <50 nmol/L: No  B-25OHD NR  Δ 25OHD >50%: NR | 2 years of therapy (DB):  D3 400 IU + Ca 1000 mg/d  Vs.  Placebo | - Burnam 8-item scale for depressive disorders (N=2,263) - Current use of antidepressants (N=36,282) | Depression was *not* the primary outcome.  Burnam scale: No effects on risk of experiencing depressive symptoms: OR 1.16 (95% CI, 0.86 to 1.56).  No effect on use of antidepressant: OR 1.01 (95% CI, 0.92 to 1.12)  Sub-group: Among women without evidence of prior depression, the Burnam-score was worsened by VitD compared with placebo (0.009; 95% CI, 0.002 to 0.017) |
| Kjærgaard, 2012 [68], Norway |  | X | X | X | N= 243 (88% F) community dwelling, aged 30-75 yr.  IC-25OHD <50 nmol/L: No  B-25OHD 47±16 nmol/L  Δ 25OHD > 50%: Yes  All were without clinical depression, and none used antidepressant | 6 mo. of therapy (DB):  D3 40 000 IU/wk.  Vs.  Placebo | BDI, HADS, SPAQ, MADRS and SCID-CV | Primary end point: Depression  No significant effect of VitD compared with placebo on depressive symptom scores  Sub-group analyses: no differences between groups according to gender, age (</≥54 yr), BMI (</≥ 28 kg/m^2^), baseline 25OHD levels (< /≥ 25 nmol/L or </≥40 nmol/L) or smoking status. |
| Yalamanchili, 2012 [211], USA |  | X |  | X | N=489 postmenopausal women aged 65-77 yr.  IC-25OHD <50 nmol/L: No  B-25OHD 31±11 nmol/L  Δ 25OHD > 50%: No | 3 yr. of therapy (DB):  Calcitriol 0.50 µg/d  Vs.  HRT  Vs  Calcitriol 0.50 µg/d + HRT  Vs.  Placebo | GDS | Depression was not the primary outcome.  No effects of calcitriol or HRT alone or in combination |
| Khoraminya, 2013 [199], Iran | X | X | X | X | N= 42 (34% F) outpatients diagnosed with a major depressive disorder, aged 18-65 yr.  IC-25OHD <50 nmol/L: No  B-25OHD 58±11 nmol/L  Δ 25OHD > 50%: Yes  None used antidepressant at inclusion. | 8 wk. of therapy (DB):  D3 1500 IU/d + fluoxetine 20 mg/d  Vs.  Placebo + fluoxetine 20 mg/d | HDRS and BDI | Primary end point: Depression  Compared with fluoxetine alone, depressive symptoms (HDRS and BDI score) decreased significantly in response to D3 + fluoxetine. |
| Mozaffari-Khosravi, 2013 [216], Iran |  |  | X |  | N=120 (72% F) patients with depression (BDI scores >17), aged 20-60 yr.  None used antidepressant at inclusion  IC-25OHD <50 nmol/L: Yes  B-25OHD: NR  Δ 25OHD > 50%: Yes | 3 mo. of follow-up after a single IM injection (NB):  D3 300,000 IU  Vs.  D3 150,000 IU  Vs.  No injection. | BDI | Primary end point: probably depression (not specifically stated)  3 mo. after injections:  Compared with no injection, BDI score improved in the group treated with 300,000 IU. |

## Table Ia. Systematic reviews on mortality

SR with MA reporting summary data on findings from RCT on effects of vitamin D supplementation on mortality.

| **Systematic reviews on effects of vitamin D supplementation on mortality** | | | | |
| --- | --- | --- | --- | --- |
| **#MA** | **Reference, publication year** | **Papers included** | **Criteria for inclusion of studies** | **Results** |
| M1 | Autier, 2007 [218] | 18 RCTs with N=57,311 participants: [2,4,7,8,14,18,27,43,219–229] | Risk of death from any cause in subjects who participated in randomized trials testing the impact of vitD supplementation (D2 D3) on any health condition. | VitD with or without Ca: RR 0.93 (95% CI, 0.87 to 0.99)  D3 trials: RR 0.93 (0.87-0.98)  No I^2^ reported.  The summary RR did not differ according to whether VitD was given alone or in combination with calcium or according to dose or duration of treatment. |
| M2 | Elamin, 2011 [6] | N=30 RCTs with N=32,191 participants [2–4,7–10,14,27,43,151,219–237] | RCT that enrolled adults who received VitD supplementation and a concurrent comparison group that did not receive VitD.  Studies were included regardless of their language, size, or duration of follow-up.  Excluded studies with activated VitD analogs | Mortality: RR 0.96 (95% CI, 0.93 to 1.00; p= 0.08], I^2^=0%  The summary RR did not differ according to whether VitD was given alone or in combination with calcium (P_i_ 0.70) or according to gender (P_i_ 0.41), duration (less/more than 12 mo.) of treatment (P_i_ 0.36), type of calciferol (D2 or D3; P_i_ 0.38), vitamin D status at baseline (P_i_ 0.19), or dose of VitD (above or below 800 IU/d, P_i_ 0.29). |
| M3 | Bjelakovic, 2011 [238] | 50 RCTs with N=94,148 participants [2–4,7,14,15,20–22,27,43,58,119,120,147–149,151,219–223,225–229,232–235,239–256] | Cochrane review. Systematic search | Overall, VitD decreased mortality (RR 0.97, 95% CI, 0.94 to 1.00, I^2^ = 0%).  When the different forms of vitamin D were assessed separately, only vitD3 decreased mortality significantly (RR 0.94, 95%CI 0.91, 0.98, I^2^ = 0%; 74,789 participants) whereas VitD2, alfacalcidol, or calcitriol did not.  VitD3 combined with calcium versus placebo or no intervention significantly decreased mortality (RR 0.95, 95%CI 0.91, 0.99, P = 0.02, I^2^ = 0%).  More than one-half of the trials had a low risk of bias.  This MA was updated in 2014 |
| M4 | Rejnmark, 2012 [257] | 24 RCTs with N=88,097 participants: [2,4,7,14,27,43,151,220–229,233,246,250,251,258–260] | A systematic literature search for publications between 1966 and 2008 in Medline, Embase, and the Cochrane Central Register of Controlled Trials on the effects of vitamin D treatment on the risk of fracture. Excluded studies on effects of treatment with activated VitD. | VitD with or without calcium: OR 0.95 (95% CI, 0.91 to 1.00, p=0.04, I^2^=0%).  VitD alone: OR 0.98 (95% CI, 0.91 to 1.06)  CaD: OR 0.94 (95% CI, 0.88 to 0.99)  Number needed to treat with vitamin D plus calcium for 3 yr. to prevent one death: 151. |
| M5 | Zheng, 2013 [261] | N=42 RCTs with N=85,466 participants  [2–4,7,15,22,25,27,43,58,63,119,120,145,148,151,219–229,233–237,240,245,246,250,253,255,260,262–264] | Searches limited to human trials, no language or time restriction was applied.  Excluded studies of patients receiving active vitamin D and intramuscular injections. | All-cause mortality with a duration of follow-up longer than 3 years: RR 0.94 (95% CI, 0.90 to 0.98, I^2^=0%)  No benefit was seen if follow-up was less than 3 years: RR 1.04 (95% CI, 0.97 to 1.12, I^2^=12%)  The following subgroups of long-term follow-up had significantly fewer deaths:  Female only RR 0.91 (95% CI, 0.83 to 1.00, I^2^=0%)  Participants with a mean age younger than 80 yrs: RR 0.93 (95% CI, 0.88 to 0.97, I^2^=0%)  Daily dose of 800 IU or less: RR 0.93 (95% CI, 0.89 to 0.98, I^2^=0%)  Participants with vitamin D insufficiency (baseline 25OHD less than 50 nmol/L: RR 0.93 (95% CI, 0.89 to 0.98, I^2^=0%)  D3 therapy: RR 0.93 (95% CI, 0.89 to 0.97, I^2^=0%)  CaD vs placebo RR 0.94 (95% CI, 0.88 to 0.99, I^2^=0%)  VitD alone did not reduce mortality: RR 0.93 (95% CI, 0.86 to 1.00, p=0.06, I^2^=0%) |
| M6 | Fortmann, 2013 [16] | 2 RCT on VitD-alone: [2,145]  1 RCT on CaD: [18] | Included fair- and good-quality RCT that assessed effects of supplements in the setting of *primary prevention* of all-cause mortality in the general adult population.  Only community-dwelling, nutrient-sufficient adults  Excluded supplement doses higher than the upper tolerable limit set by the U.S. Food and Nutrition Board | Pooled unadjusted summary risk:  VitD alone: RR 0.94 (95% Cl, 0.87 to 1.01)  CaD: RR 0.92 (95% Cl, 0.83 to 1.01).  No I^2^ reported |
| M7 | Avenell, 2014 [265] | 29 RCTs with N=71,032 participants:  [2,3,7,10,14,20,21,26,27,58,123,150,151,219,220,223,224,226,228,229,233,235,244,247,250,256,266–268] | Cochrane review on fracture risk which also assessed mortality in response to VitD supplementation | VitD with or without Ca (29 trials, 71,032 participants): RR 0.97 (95% Cl, 0.93 to 1.01, I^2^=0%)  CaD: RR 0.94, (95% Cl, 0.87 to 1.02, I^2^=7%)  VitD alone: RR 0.99, (95% Cl, 0.94 to 1.03, I^2^=0%) |
| M8 | Bjelakovic, 2014 [146] | Cochrane review including 15 RCTs with N=49,866 participants  [2,3,7,15,20,21,26,123,145,147–152] | Cochrane review on cancer risk, which also assessed mortality. Included RCTs that compared VitD at any dose, duration, and route of administration versus placebo or no intervention in adults | All-cause mortality: RR 0.93 (95% CI 0.88 to 0.98) I² =0%  Trials with low risk of bias: RR 0.90 (95% CI 0.77 to 1.07) I² =NA)  Trials with high risk of bias RR 0.93 (95% CI 0.88 to 0.998) I² =0% |
| M9 | Bjelakovic, 2014 [269] | 56 RCT with N=95,286 participants: [2–4,7,15,20–22,27,43,58,119,120,147–151,219–223,225–230,232–235,239–255][25,112,123,145,256,270] | Cochrane review on mortality | VitD with or without Ca (56 trials): RR 0.97 (95% CI, 0.94 to 0.99, I^2^ = 0%)  Type of calciferol:   - D3: RR 0.93 (95% CI, 0.89 to 0.98) - D2: RR 1.02 (95% CI, 0.96 to 1.08)   VitD alone or combined with Ca:   - VitD3 alone: RR 0.92 (95% CI, 0.85 to 1.00) - CaD3: RR 0.96 (95% CI, 0.92 to 0.99)   Vitamin D status:   - VitD insufficiency: RR 0.95 (95% CI, 0.91 to 0.99) - VitD adequacy: RR 0.95 (95% CI, 0.87 to 1.05) - Unknown status: RR 1.02 (95% CI, 0.92 to 1.13) |
| M10 | Bolland, 2014 [30] | 38 RCTs with N=81,173 participants:  [2–4,7,8,10,14,15,18,22,27,119,120,123,148,151,219–229,233–236,239,240,244,245,251,253,255,260] | Through a systematic search on PubMed, the authors identified MA (published since January 2009) reporting data from RCT on effects of VitD supplementation on risk of cancers. Four MA were identified [1,6,31,32] reporting data from a total of RCT. A new MA was performed on data from the 7 RCT.  Studies were excluded if they were cluster randomized or investigated effects of hydroxylated VitD or VitD analogues. The analyses also excluded trials that included other interventions only in the VitD group, trials of fortified dairy products, and trials in populations with chronic comorbidity other than osteoporosis or frailty | VitD with or without Ca: RR 0.96 (95% CI, 0.93 to 1.00, p=0.04, I^2^ =0%)  Vit D alone: RR 0.97 (95% CI, 0.92 to 1.01; I^2^=0%)  CaD: RR 0.96 (95% CI, 0.89 to 1.02; I^2^=0%) |
| M11 | Chowdhury, 2014 [271] | N=22 RCTs with N=30,716 participants: [2,25,33,43,58,145,147,151,220,225,226,232–234,239,240,248,250,251,253,263,272] | Systematic search - Included only RCTs on effects of VitD supplementation alone in adults compared with a placebo or no treatment.  Data on cause specific death or all-cause mortality endpoints were collected. | All studies (D2 or D3): RR 0.98 (95% CI, 0.94 to 1.02).  D3 studies: RR 0.89 (95% CI, 0.80 to 0.99, I^2^=10 %)  D2 studies: RR 1.04 (95% CI, 0.97 to 1.11, I^2^=7 %)  For D3 trials, the overall effect did not vary significantly across sex (P_i_ 0.23), population source (P_i_ 0.64), daily dose (P_i_ 0.50), or duration of the intervention (P_i_ 0.56) or of the follow-up (P_i_ 0.22).  For D2 trials, mortality was increased in trials that used an intervention dose of 600 to 2000 IU/day, had shorter average intervention period (<1.5 v ≥1.5 years), and had high risk of bias. |
| M12 | Zheng, 2015 [273] | 7 RCTs with N=21,203 participants  [2,151,225,226,233,250,251] | Only RCTs on intermittent, high dose vitamin D treatment on all-cause mortality among older (>65 yr.) adults. | No effect of supplementation on all-cause mortality: RR 1.04 (95% CI, 0.91 to 1.17, I^2^ 64%).  Sub-group analysis showed no change in risk ratio according to gender, participant residency, and type of vitamin D, route of administration, baseline 25OHD levels or trial duration. |

## Table Ib. RCTs on mortality

Characteristics of randomized clinical trials included in MA (Table 10A) on effects of vitamin D supplementation on mortality.

| **Characteristics of the RCTs included in MAs on effects of vitamin D supplementation on mortality** | | | | | | | | | | | | | | | |
| --- | --- | --- | --- | --- | --- | --- | --- | --- | --- | --- | --- | --- | --- | --- | --- |
| **Reference, publication year, country** | **Included in MA** | | | | | | | | | | | | **Population** | **Intervention** | **Results** |
|  | 1 | 2 | 3 | 4 | 5 | 6 | 7 | 8 | 9 | 10 | 11 | 12 |  |  |  |
| Brohult, 1973 [230], Sweden |  | X |  |  |  |  |  |  | X |  |  |  | N= 49 (68% F) patients with rheumatoid arthritis  Mean age 52 yr.  IC-25OHD $<$ 50 nmol/L: No  B-25OHD: NR  Δ 25OHD > 50%: NR | 1 yr.of treatment (DB):  calciferol 100,000 IU/d  Vs.  Placebo | RR 3.00 (95% Cl, 0.13 to 70.30) |
| Grove, 1981 [231], Denmark |  | x |  |  |  |  |  |  |  |  |  |  | N= 22 postmenopausal women with backache and a halisteretic spine with at least one compression fracture  Mean age 73.9 yr.  IC-25OHD $<$ 50 nmol/L: No  B-25OHD: NR  Δ 25OHD > 50%: NR | 3 mo. of treatment (DB):  Calciferol 50,000 IU twice weekly+20 ng sodium fluoride/d + Ca 500 mg/D  Vs  Placebo | RR 3.23 (95% Cl, 0.14 to 72.46) |
| Inkocaara, 1983 [10], Finland |  | x |  |  |  |  | X |  |  | X |  |  | N= 327 (83% F) residents or elderly care homes.  Mean age: 79.5±7.1 yr.  IC-25OHD $<$ 50 nmol/L: No  B-25OHD: nmol/L: NR  Δ 25OHD > 50%: NR | 9 mo. of treatment (DB):  D3 1000 IU/d  Vs.  Ca 1200 mg/d  Vs.  Methandienone  Vs.  D3 1000 IU/d  + Ca 1200 mg/d  Vs.  D3 1000 IU/d  + Ca 1200 mg/d  + methandienone 2.5 mg/d  Vs.  D3 1000 IU/d  + methandienone 2.5 mg/d  Vs.  Ca 1200 mg/d  + methandienone 2.5 mg/d  Vs.  Placebo | RR 1.31 (95% Cl, 0.45 to 3.80) |
| Corless, 1985 [239], UK |  |  | X |  |  |  |  |  | X | X | X |  | N= 62 (82% F) elderly patients from the geriatric walls.  Mean age: 82.4 yr.  IC-25OHD $<$ 50 nmol/L: No  B-25OHD: 55 nmol/L  Δ 25OHD > 50%: Yes | 9 mo of treatment (DB):  D2 9000 IU/d  Vs  placebo | RR 1.00 (95% Cl, 0.42 to 2.41) |
| Ott, 1989 [20], USA |  |  | X |  |  |  | X | X | X |  |  |  | N=86 postmenopausal women with vertebral compression fractures.  Mean age. 67.5 yr.  IC-25OHD $<$ 50 nmol/L: No  B-25OHD: 66 nmol/L  Δ 25OHD > 50%: NR | 24 mo treatment (DB) with  Calcitriol 0.43 µg/d  Vs  Placebo | RR 0.33 (95% Cl, 0.01 to 7.96) |
| Gallagher, 1990 [266], USA |  |  |  |  |  |  | X |  | X |  |  |  | N=50 postmenopausal women with one or more previous non-traumatic vertebral fracture.  Mean age 70 yr.  IC-25OHD < 50 nmol/L: No  B-25OHD: NR  Δ 25OHD > 50%: NR | 24 mo.of treatment (DB):  Calcitriol mean dose 0.62 μg/d + Ca 800-1000 mg/d  Vs  D2 400 IU/d + placebo | RR 3.00 (95% Cl, 0.13 to 70.30) |
| Grady, 1991 [147], USA |  |  | X |  |  |  |  | X |  |  | X |  | N= 98 (54% F) elderly, aged > 69 yr.  Mean age 79.1 ±5.4 yr.  IC-25OHD $<$ 50 nmol/L: No  B-25OHD: 60.4 nmol/L  Δ 25OHD > 50%: No | 6 mo of treatment (DB):  Calcitriol 0.5 µg/d  Vs  placebo | RR 2.88 (95% Cl, 0.12 to 67.07) |
| Chapuy, 1992 [219], France | X | X | X |  | X |  | X |  | X | X |  |  | N=3,270 (100% F) healthy ambulatory women  Mean aged 84±6 yr.  IC-25OHD $<$ 50 nmol/L: No  B-25OHD: 40 nmol/L  Δ 25OHD > 50%: Yes | Up to 18 months, treatment (DB):  D3 800 IU/d + Ca 1200 mg/d  Vs  Placebo.  Follow up, 4 yr. | RR 0.98 (95% Cl, 0.92 to 1.04) |
| Tilyard, 1992 [267], NZ |  |  |  |  |  |  | X |  |  |  |  |  | N= 622 (100% F) ambulatory post-menopausal women with osteoporosis and 1 or more vertebral compression fracture  Mean aged 64±7 yr.  IC-25OHD $<$ 50 nmol/L: No  B-25OHD: NR  Δ 25OHD > 50%: NR | 36 mo. of treatment (SB):  Calcitriol 0.5 µg/d  Vs  Ca 1000 mg/d | RR 1.13 (95% Cl, 0.41 to 3.09) |
| Chapuy, 1994 [258], France |  |  |  | X |  |  |  |  |  |  |  |  | N=3,270 (100% F). nursing home residents  Mean age 84±6 yr.  IC-25OHD $<$ 50 nmol/L: No  B-25OHD: NR  Δ 25OHD > 50%: NR | Up to 36 mo. of treatment (DB):  D3 800 IU/d + Ca 1200 mg/d  vs.  Placebo | RR 1.01 (95% CI, 0.92 to 1.10). |
| Ooms, 1995 [240], The Netherlands |  |  | X |  | X |  |  |  | X | X | X |  | N=348 (100% F) elderly  Mean age 80.3± 5.6 yr.  IC-25OHD $<$ 50 nmol/L: No  B-25OHD: median 27 nmol/L  Δ 25OHD > 50%: Yes | Up to 24 mo. of treatment (DB):  D3 400 IU/d  Vs.  Placebo | RR 0.51 (95% Cl, 0.25 to 1.02) |
| Lips, 1996 [220], The Netherlands | X | X | X | X | X |  | X |  | X | X | X |  | N=2,578 (74% F) living independently in apartments/ homes for elderly persons, aged ≥70y  Mean age 80±6 yr.  IC-25OHD $<$ 50 nmol/L: No  B-25OHD: 27 nmol/L  Δ 25OHD > 50%: Yes | Up to 42 mo. (DB) treatment with  D3 400 IU/d  vs.  placebo | RR 0.92 (95% Cl, 0.80 to 1.06) |
| Dawson-Hughes, 1997 [119], USA |  |  | X |  | X | X |  |  | X | X |  |  | N=389 (55% F) healthy ambulatory participants  Mean age 61.9 yr.  IC-25OHD $<$ 50 nmol/L: No  B-25OHD: 72 nmol/L  Δ 25OHD > 50%: No | Up to 36 mo treatment (DB) with  D3 700 IU/d + Ca 500 mg/d  Vs  placebo | RR 1.08 (95% Cl, 0.15 to 7.59) |
| Sato, 1997 [241], Japan |  |  | X |  |  |  |  |  | X |  |  |  | N=64 (45% F) outpatients with hemiplegia after stroke.  Mean age 68.5 yr.  IC-25OHD $<$ 50 nmol/L: No  B-25OHD: NR  Δ 25OHD > 50%: NR | 6 mo. of treatment (DB) with  Alfacalcidol 1 µg/d + Ca 300 mg/d  Vs  Placebo + Ca 300 mg/d | RR 0.87 (95% Cl, 0.06 to 13.40) |
| Baeksgaard, 1998 [221], Denmark | X | X | X | X | X |  |  |  | X | X |  |  | N=240 healthy postmenopausal women, aged 58–67 yr.  Mean age 62.5 yr.  IC-25OHD $<$ 50 nmol/L: No  B-25OHD: nmol/L: NR  Δ 25OHD > 50%: NR | 24 mo. of treatment (DB):  D3 560 IU/d + Ca 1000 mg/d  vs.  D3 560 IU/d (in a multivitamin) + Ca 1000 mg/d  vs.  Placebo | RR 0,33 (95% CI 0,01 to 8.06) |
| Sato 1999 a, [242], Japan |  |  | X |  |  |  |  |  | X |  |  |  | N=86 (78 % F) elderly patients with Parkinson’s disease  Mean age 70.6 yr.  IC-25OHD $<$ 50 nmol/L: No  B-25OHD: 27.5 nmol/L  Δ 25OHD > 50%: No | 12 mo treatment (DB) with  Alfacalcidol 1 µg/d  Vs  Placebo | RR 3.00 (95% Cl, 0.13 to 71.65) |
| Sato 1999, [243], Japan |  |  | X |  |  |  |  |  | X |  |  |  | N= 105 (56% F) outpatients with hemiplegia after stroke.  Mean age 70.7 yr.  IC-25OHD $<$ 50 nmol/L: No  B-25OHD: 28.5 nmol/L  Δ 25OHD > 50%: No | 12 mo. treatment (NB):  Alfacalcidol 1 µg/d  Vs  Ipriflavone 600 mg/d  Vs  None | RR 0.34 (95% Cl, 0.01 to 8.13) |
| Komulainen, 1999 [7], Finland | X | X | X | X | X |  | X | X | X | X |  |  | N=458 early postmenopausal women  Mean age: 52.9 ±0.4 yr.  IC-25OHD $<$ 50 nmol/L: No  B-25OHD nmol/L: NR  Δ 25OHD > 50%: NR | Up to 60 mo. treatment (NB):  HRT  Vs  D3 300 IU/d (only100 IU/d during the 5^th^ year):  Vs  HRT + D3 300 IU/d (only100 IU/d during the 5^th^ year)  Vs  Placebo | RR 0.33 (95% Cl, 0.01 to 7.48) |
| Krieg, 1999 [222], Switzerland | X | X | X | X | X |  |  |  | X | X |  |  | N=248 institutionalized women aged 62–98 yrs.  Mean age: 85±8 yr.  IC-25OHD $<$ 50 nmol/L: No  B-25OHD: 29.8 nmol/L  Δ 25OHD > 50%: Yes | 24 mo. of treatment (NB)  D3 880 IU/d + 1000 mg Ca/d  Vs  None | RR 0.81 (95% Cl, 0.48 to 1.36) |
| Gallagher, 2001/2004, [21,33], USA |  |  | X |  |  |  | X | X | X |  | X |  | N= 489 elderly women  Mean age: 71±4 yr.  IC-25OHD $<$ 50 nmol/L: No  B-25OHD: 78 nmol/L  Δ 25OHD > 50%: No | 36 mo. of treatment (DB):  Estrogen (hysterectomized)  Vs  Hormone RT  vs.  Calcitriol 0.5 µg/d  Vs  Calcitriol 0.5 µg/d + hormone/estrogen RT  Vs  Placebo  Follow up for 60 mo. | RR 2.00 (95% CI, 0.18 to 21.77) |
| Chapuy, 2002 [223], France | X | X | X | X | X |  | X |  | X | X |  |  | N=583 ambulatory institutionalized women  Mean age 85±7 yr.  IC-25OHD $<$ 50 nmol/L: No  B-25OHD: 22 nmol/L  Δ 25OHD > 50%: Yes | 24 mo. of treatment (DB):  D3 800 IU/d + 1200 mg Ca/d  Vs  placebo | RR 0.76 (95% Cl, 0.55 to 1.06) |
| Meyer, 2002 [224], Norway | X | X |  | X | X |  | X |  |  | X |  |  | N=1,144 (75.9% F) nursing home residents  Age 85 (81-90) yr.  IC-25OHD $<$ 50 nmol/L: No  B-25OHD: 47 nmol/L  Δ 25OHD > 50%: No | 24 mo. of treatment (DB):  D3 400 IU/d  Vs.  Placebo | RR 1.01 (95% Cl, 0.89 to 1.16) |
| Bischoff, 2003 [244], Switzerland |  |  | X |  |  |  | X |  | X | X |  |  | N= 122 elderly women living in institutional care  Mean age 85.3 yr.  IC-25OHD $<$ 50 nmol/L: No  B-25OHD: 31 nmol/L  Δ 25OHD > 50%: Yes | 4 mo. of treatment (DB):  D3 800 IU/d + Ca 1200 mg/d  Vs.  Placebo | RR 0.24 (95% Cl, 0.03 to 2.10) |
| WHI study, USA  Jackson, 2003 [268]  Wactawski-Wende, 2006 [18]  Brunner, 2008 [254], LaCroix, 2009 [259] | X | X | X | X | X | X | X |  | X | X |  |  | N=36,282 postmenopausal women  IC-25OHD $<$ 50 nmol/L: No  B-25OHD: NR  Δ 25OHD > 50%: NR | 7 years of treatment (DB):  D3 400 IU + Ca 1000 mg/d  Vs.  Placebo | RR 0.92 (95 % Cl, 0.83 to 1.01) |
| Latham, 2003 [225], NZ | X | X | X | X | X |  |  |  | X | X | X | X | N=243 (53% F) frail elderly aged > 65 yrs  Mean age: 79± 7 yr.  IC-25OHD $<$ 50 nmol/L: No  B-25OHD: 37.50 nmol/L  Δ 25OHD > 50%: No | DB study:  D3 300,000 IU/single dose  Vs  placebo | 6 mo. of follow-up.  RR 3.70 (95% Cl, 1.06 to 12.92) |
| Cooper, 2003 [245], AU |  |  | X |  | X |  |  |  | X | X |  |  | N=187 postmenopausal women.  Mean age 80 yr.  IC-25OHD $<$ 50 nmol/L: No  B-25OHD: 81.6 nmol/L  Δ 25OHD > 50%: No | 24 mo. of treatment (DB):  D2 10,000 IU/wk + Ca 1000 mg/d  Vs  Placebo + Ca 1000 mg/d | RR 0.34 (95% Cl, 0.01 to 8.16) |
| Trivedi, 2003 [2], UK | X | X | X | X | X | X | X | X | X | X | X | X | N=2,686 (24% F) community-dwellings, aged 65­85 yr.  Mean age 75±5 yr.  IC-25OHD $<$ 50 nmol/L: No  B-25OHD: 74.3 nmol/L  Δ 25OHD > 50%: NR | 60 mo. of treatment (DB):  D3 100 000 IU fourmonthly  Vs  Placebo fourmonthly | RR 0.88 (95% CI, 0.74 to 1.06) |
| Harwood, 2004 [226], UK | X | X | X | X | X |  | X |  | X | X | X | X | N= 150 elderly women following surgery for hip fracture  Mean age: 83 yr.  IC-25OHD $<$ 50 nmol/L: No  B-25OHD: 29 nmol/L  Δ 25OHD > 50%: No | Up to 12 months treatment (NB):  D2 300,000 IU/single dose intramuscular  vs  D2 300,000 IU/single dose intramuscular +Ca 1000 mg/d  Vs  D3 800 IU/d + Ca 1000 mg/d  Vs.  None | RR 1.57 (95% Cl, 0.65 to 3.82) |
| Avanell, 2004 [27], UK | X | X | X | X | X |  | X |  | X | X |  |  | N= 134 (83 % F) with a history of an osteoporotic fracture within the last 10 yr., aged > 70 yr.  Mean age 77±5 yr.  IC-25OHD $<$ 50 nmol/L: No  B-25OHD: nmol/L: NR  Δ 25OHD > 50%: NR | 12 months of treatment (NB):  D3 800 IU/d + 1000 mg Ca:  vs*.*  None | RR 0.35 (95% Cl, 0.02 to 5.50). |
| Meier, 2004 [227], Germany | X | X | X | X | X |  |  |  | X | X |  |  | N= 55 (65% F) healthy volunteers  Mean age 56.5 yr.  IC-25OHD $<$ 50 nmol/L: No  B-25OHD: 75.3 nmol/L  Δ 25OHD > 50%: No | 6 mo. of treatment (NB):  D3 500 IU/d + Ca 500 mg/d  Vs  None | RR 0.28 (95% Cl, 0.01 to 6.58) |
| Larsen, 2004 [246], Denmark |  |  | X | X | X |  |  |  | X |  |  |  | N= 9,605 (60.1% F) community dwelling, aged ≥66yrs  Median age 74 yr.  IC-25OHD $<$ 50 nmol/L: No  B-25OHD: 37 nmol/L  Δ 25OHD > 50%: No | 42 mo. Of treatment (NB):  D3 4000/d + 1000 mg Ca/d  Vs  None | RR 0.93 (95% Cl, 0.12 to 1.07) |
| Dukas, 2004 [247], Switzerland |  |  | X |  |  |  | X |  | X |  |  |  | N=378 (51% F) elderly people  Mean age: 71 yr.  IC-25OHD $<$ 50 nmol/L: No  B-25OHD: 38 nmol/L  Δ 25OHD > 50%: NR | 36 wk of treatment (DB):  Alfacalcidol, 1 µg/d  Vs  Placebo | RR 0.97 (95% Cl, 0.06 to 2.73) |
| Brazier, 2005 [4], France | X | X | X | X | X |  |  |  | X | X |  |  | N= 192 elderly vitamin D-insufficient women  Mean age 74.6 yr.  IC-25OHD $<$ 50 nmol/L: Yes  B-25OHD: 18.3 nmol/L  Δ 25OHD > 50%: Yes | 1 yr. treatment (DB) with  D3 800 IU/d + 1000 mg Ca/d  Vs.  Placebo | RR 3.06 (95% Cl, 0.32 to 28.93) |
| Aloia, 2005 [120], USA |  |  | X |  | X |  |  |  | X | X |  |  | N=208 black postmenopausal African-American women.  Mean age 61 ± 6 yr.  IC-25OHD $<$ 50 nmol/L: No  B-25OHD: 48 nmol/L  Δ 25OHD > 50%: No | 36 mo. of treatment (DB):  D3 800 IU/d (the first two years), 2000 IU/d (the third year) + Ca 1200-1500 mg/d  Vs  Placebo Vit D and Ca/1200-1500 mg/d | RR 0.50 (95% CI, 0.05 to 5.43). |
| Porthouse, 2005 [228], UK | X | X | X | X | X |  | X |  | X | X |  |  | N=3,314 (100% F) GP patients >70 yr. with risk factors for fracture  Mean age: 76 yr  IC-25OHD $<$ 50 nmol/L: No  B-25OHD: NR  Δ 25OHD > 50%: NR | Median duration of treatment (NB) 25 mo (range 18-42)  D3 800 IU/d + Ca 1000 mg/d+ leaflet on dietary calcium intake  Vs.  Leaflet alone | RR 1.26 (95% Cl, 0.90 to 1.79) |
| Flicker, 2005 [229], Austria | X | X | X | X | X |  | X |  | X | X |  |  | N=625 (95%) nursing home residents  Age 83±8 yr.  IC-25OHD $<$ 50 nmol/L: No  B-25OHD: NR  Δ 25OHD > 50%: NR | Up to 24 mo. (DB) treatment  D2, initially 10,000 IU/w, later 1000 IU/d (because of the discontinuation of this preparation) + 600 mg Ca  Vs.  placebo | RR 0.89 (95% Cl, 0.68 to 1.16) |
| RECORD, 2005, UK  Grant, 2005 [14]  Avenell, 2012 [145] | X | X | X | X | X | X | X | X | X | X | X |  | N=5,292 (84.7% F) with a previous osteoporotic fracture  Mean age 77 yr.  IC-25OHD $<$ 50 nmol/L: No  B-25OHD: NR  Δ 25OHD > 50%: NR | Treatment (DB) in 24-62 mo. (median 30.4)  D3 800 IU/d + Ca 1000 mg/d  Vs  D3 800 IU/d  Vs.  1000 mg Ca  Vs.  double placebo | RR 0.95 (95% Cl, 0.84 to 1.07) |
| Sato, 2005 [248], Japan |  |  | X |  |  |  |  |  | X |  | X |  | N= 99 hospitalized elderly women with post-stroke hemiplegia  Mean age 74.1 ±4 yr.  IC-25OHD $<$ 50 nmol/L: Yes  B-25OHD: 24.5 nmol/L  Δ 25OHD > 50%: Yes | 24 mo. of treatment (DB):  D2 1000 IU/d  Vs.  Placebo | RR 0.50 (95% Cl, 0.05 to 5.33) |
| Moschonis, 2006 [249], Greece |  |  | X |  |  |  |  |  | X |  |  |  | N=112 healthy postmenopausal women.  Mean age 60.3 yr.  IC-25OHD $<$ 50 nmol/L: No  B-25OHD: NR  Δ 25OHD > 50%: NR | 12 mo. of treatment (NB):  D3 300 IU/d + Ca 1200 mg/d  Vs  None | RR 0.55 (95% Cl, 0.02 to 13.21) |
| Law, 2006 [250], UK |  |  | X | X | X |  | X |  | X |  | X | X | N=3,717 (76% F) nursing home residents  Mean age: 85 yr.  IC-25OHD $<$ 50 nmol/L: No  B-25OHD: 47 nmol/L  Δ 25OHD > 50%: No | Treatment (NB) for a median of 10 (IQR 7–14) mo.  D2 100,000 IU/threemonthly  Vs.  None | RR 1.20 (95% Cl, 1.04 to 1.37) |
| Campbell, 2005 [232], NZ |  | X | X |  |  |  |  |  | X |  | X |  | N= 391 (68% F) elderly with visual impairment.  Mean age 83.6 yr.  IC-25OHD $<$50 nmol/L: No  B-25OHD: NR  Δ 25OHD > 50%: NR | 12 mo. of treatment (NB):  Home safety assessment  Vs  Calciferol 2.5 mg/month + exercise program  Vs.  Both interventions  Vs.  Social visits | RR 0.60 (95% Cl, 0.22 to 1.63) |
| Schleithoff, 2006 [43], Germany | X | X | X | X | X |  |  |  | X |  | X |  | N= 123 (49% F) patients with congestive heart failure.  Mean age 55.5 yr.  IC-25OHD $<$ 50 nmol/L: No  B-25OHD: 36 nmol/L  Δ 25OHD > 50%:No | Treatment for 9 mo. (DB) with:  D3 2000 IU/D+ Ca 500 mg/d  Vs.  Placebo + Ca 500 mg/d | Follow up for 16 mo.  RR 1.19 (95% Cl, 0.42 to 3.33) |
| Burleigh, 2007 [235], UK |  | X | X |  | X |  | X |  | X | X |  |  | N= 205 (59 % F) elderly geriatric inpatients  Mean age 84±8 yr.  IC-25OHD $<$50 nmol/L: No  B-25OHD: 22 nmol/L  Δ 25OHD > 50%: No | For 0.96 mo. (until discharge or death) treatment with;  D3 800 IU/d + Ca 1200 mg/d  Vs.  Ca 1200 mg/d | RR 1.27 (95% Cl, 0.64 to 2.50) |
| Broe, 2007 [234], USA |  |  | X |  | X |  |  |  | X | X | X |  | N= 124 (73% F) nursing home residents  Mean age 89±6 yr.  IC-25OHD $<$50 nmol/L: No  B-25OHD: 49 nmol/L  Δ 25OHD > 50% : NR | 5 mo. of treatment (DB):  D2 200/d  Vs.  D2 400/d  Vs.  D2 600/d  Vs.  D2 800/d  Vs.  Placebo | RR 0.63 (95% Cl, 0.13 to 3.07) |
| Lyons, 2007 [233], UK |  | X | X | X | X |  | X |  | X | X | X | X | N=3,440 (F 76%) nursing home residents  Mean age 84±8 yr.  IC-25OHD $<$50 nmol/L: No  B-25OHD: 80 nmol/L  Δ 25OHD > 50%: NR | Up to 36 mo of treatment (DB) with  D2 100,000 IU/fourth monthly  Vs.  Placebo | RR 0.99 (95% Cl, 0.93 to 1.05) |
| Bolton-Smith, 2007 [148], UK |  |  | X |  | X |  |  | X | X | X |  |  | N= 244 elderly non-osteoporotic women  Mean age 68 yr.  IC-25OHD $<$50 nmol/L: No  B-25OHD: 62.5 nmol/L  Δ 25OHD > 50%: No | 2 yr. of treatment of (DB):  D3 400 IU/d + 1000 mg Ca/d  Vs.  Vit K1, 200 µg/d + 1000 mg Ca/d  Vs.  D3 400 IU/d + Vit K1 200 µg/d + 1000 mg Ca/d  Vs.  Placebo | RR 0.33 (95% Cl, 0.01 to 8.16) |
| Smith 2007 [251], UK |  |  | X | X |  |  |  |  | X | X | X | X | N=9,440 (53.9%) GP patients presenting for influenza vaccination  Median age 79 yr.  IC-25OHD $<$50 nmol/L: No  B-25OHD: 56.5 nmol/L  Δ 25OHD > 50%: No | 36 mo. of treatment (DB) with intramuscular  D2 300 000 IU/yearly  Vs.  placebo/yearly | RR = 1.00 (95% Cl, 0.87 to 1.15) |
| Lappe, 2007 [15], USA |  |  | X |  | X |  |  | X | X | X |  |  | N=1,179 healthy postmenopausal white women  Mean age 66.7 yr.  IC-25OHD $<$50 nmol/L: No  B-25OHD: 71.8 nmol/L  Δ 25OHD > 50%: No | 48 mo. of treatment (DB):  D3 1000 IU/d+ Ca 1400-1500 mg/d  Vs  Ca 1400-1500 mg/d  Vs  Placebo | RR 0.37 (95% Cl, 0.12 to 1.07) |
| Beer,2007 [272], USA |  |  |  |  |  |  |  |  |  |  | X |  | N= 250 men with prostate metastatic cancer, aged > 18 yrs.  Mean age 79 yr  IC-25OHD $<$50 nmol/L: No  B-25OHD: nmol/L: NR  Δ 25OHD > 50%: NR | Treatment (DB) with  Calcitriol 45 µg/once per week 3 out of four weeks.  Vs  placebo  NB: Duration of intervention not reported.  Both groups also received chemotherapy (doctaxel) | RR 0.75 (95% Cl, 0.17 to 3.28) |
| Berggren, 2008 [9], Sweden |  | x |  |  |  |  |  |  |  |  | X |  | N= 199 (74% F) patients operated on for femoral neck fracture, aged ≥70 years  Mean age 82 yr.  IC-25OHD <50 nmol/L: No  B-25OHD: NR  Δ 25OHD > 50%: NR | 1 yr. of treatment (NB):  D3 800 IU/d + Ca 1000 mg/d  Vs.  None  A multidisciplinary, multifactorial fall-prevention program was applied in the intervention group. | RR 0.85 (95% CI, 0.46 to 1.56)  The intervention group had a higher prescription of calcium and vitamin D (66%) than the control group (22%) |
| Zhu, 2008 [22], AU |  |  | X |  | X |  |  |  | X | X |  |  | N=120 elderly women.  Mean age 74.8±2.6 yr.  IC-25OHD <50 nmol/L: No  B-25OHD 68±29 nmol/L  Δ 25OHD > 50%: Yes | 60 mo. of treatment (DB):  D2 1000 IU/d + Ca 1200 mg/d  Vs.  Placebo + Ca 1200 mg/d | RR 0.41 (95% Cl, 0.02 to 8.34) |
| Chel, 2008 [253], The Netherlands |  |  | X |  | X |  |  |  | X | X | X |  | N= 338 (77 % women) nursing home residents.  Mean age 84±6.3 yr.  IC-25OHD $<$50 nmol/L: No  B-25OHD: 25 nmol/L  Δ 25OHD > 50%: Yes | 4 mo. of treatment (DB):  D3 600 IU/d  Vs.  D3 4200/wk.  Vs.  D3 18.000 IU/mo.  Vs.  Placebo | RR 0.78 (95% Cl 0.49 to 1.26) |
| Bjorkman, 2008 [236], Finland |  | X | X |  | X |  |  |  | X | X |  |  | N=218 (82% F) long-term inpatients  Mean age 84.5 yr.  IC-25OHD $<$50 nmol/L: No  B-25OHD: 23 nmol/L  Δ 25OHD > 50%: Yes | 6 mo. of treatment (DB):  D3 1200 IU/d + Ca 500 mg/d  Vs.  D3 400 IU/d + Ca 500 mg/d  Vs.  Placebo + Ca 500 mg/d | RR 1.36 (95% CI, 0.68 to 2.73) |
| Daly, 2008 [149], AU |  |  | X |  |  |  |  | X | X |  |  |  | N=167 healthy ambulatory men  Mean age: 61.9 ±7 yr.  IC-25OHD $<$50 nmol/L: No  B-25OHD: NR  Δ 25OHD > 50%: NR | Up to 24 months of treatment (NB):  D3 800 IU/d + Ca 1000 mg/d  vs  None | Follow up for 42 months:  RR 2.90 (95% Cl, 0.12 to 70.07) |
| Prince, 2008 [3], AU |  | X | X |  | X |  | X | X | X | X |  |  | N=302 elderly women with a history of falling and vitamin D insufficiency  Mean age ≈77±5 yr.  IC-25OHD <50 nmol/L: No  B-25OHD: ≈45±12 nmol/L  Δ 25OHD > 50%: No | 12 mo. of treatment (DB):  D2 1000 IU/d+ Ca 1000 mg/d  Vs  Placebo + Ca 1000 mg/d | RR 0.33 (95% Cl, 0.01 to 8.12) |
| Wejse, 2009 [237], Denmark |  | x |  |  | X |  |  |  |  |  |  |  | N= 187 (39% F) patients with tuberculosis  Mean age 37 yr.  IC-25OHD $<$50 nmol/L: No  B-25OHD: 77.5mol/L  Δ 25OHD > 50%: No | 12 mo. of treatment (DB):  D3 100,000 IU at 0, 5 and 8 months  Vs.  Placebo | RR 1.26 (95% Cl, 0.76 to 2.08) |
| Lips, 2010 [255], The Netherlands |  |  | X |  | X |  |  |  | X | X |  |  | N= 226 (%F NR) elderly >70 yr. with vitamin D insufficiency.  IC-25OHD $<$50 nmol/L: Yes  B-25OHD: nmol/L: 34.6 nmol/L  Δ 25OHD > 50%: No | 3 mo. of treatment (DB):  D3 8400/weekly + Ca 500 mg/d (if dietary calcium intake <1000 mg/d)  Vs  Placebo | RR 2.95 (95% Cl, 0.12 to 71.60) |
| Sanders, 2010 [151], AU |  | X | X | X | X |  | X | X | X | X | X | X | N=2256 (100 % F) community-dwelling women aged > 70 yr. and at risk of fracture  Mean age: 76.0 yr.  IC-25OHD <50 nmol/L: No  B-25OHD 49 nmol/L  Δ 25OHD > 50%: No | 3–5 yr of treatment (DB):  D3 500,000 IU/yr  Vs.  Placebo | RR 0.85 (95% Cl, 0.56 to 1.28) |
| Salovaara, 2010 [260], Finland |  |  |  | X | X |  |  |  |  | X |  |  | N= 3,195 community dwelling women  Mean age: 67 yr.  IC-25OHD $<$ 50 nmol/L: No  B-25OHD: 50.0 nmol/L  Δ 25OHD > 50%: No (49 %) | Up to 36 mo. treatment (NB) with  D3 800 IU/d + Ca 1000/D  Vs  None | RR 0.85 (95% Cl, 0.43 to 1.67) |
| Witham, 2010 [58], Scotland |  |  | X |  | X |  | X |  | X |  | X |  | N=105 (34% F) patients with systolic heart failure  Mean age 79.7 yr.  IC-25OHD $<$50 nmol/L: Yes  B-25OHD: 23.7 nmol/L  Δ 25OHD > 50%: Yes | 3 mo. of treatment (DB):  D2 100,000 at baseline and after 10 weeks  Vs.  Placebo | Follow up for 6 mo.  RR 1.96 (95% Cl, 0.38 to 10.26) |
| Kärkkäinen, 2010 [256], Finland |  |  | X |  |  |  | X |  | X |  |  |  | N= 3139 postmenopausal women  Mean age: 67.4 ±2.0 yr.  IC-25OHD $<$ 50 nmol/L; No  B-25OHD: 50.1 nmol/L  Δ 25OHD > 50%: No | 36 mo. of treatment (NB):  D3 300 IU/d + Ca 1000 mg/d  Vs  None | RR 1.15 (95% Cl, 0.55 to 2.41) |
| Janssen, 2010 [150], The Netherlands |  |  |  |  |  |  | X | X | X |  |  |  | N=70 elderly vitamin D insufficient women  Mean age 82.4±6.4 yr.  IC-25OHD $<$50 nmol/L: Yes  B-25OHD: 32.6 nmol/L  Δ 25OHD > 50%: Yes | 6 mo. of treatment (DB):  D3 400 IU/d + 500 mg Ca/d  Vs.  Placebo + 500 mg Ca/d | RR 0.32 (95% Cl, 0.01 to 7.48) |
| Cherniack, 2011 [270], USA |  |  |  |  |  |  |  |  | X |  |  |  | N=46 (2% F) elderly people  Mean age: 80 yr.  IC-25OHD $<$50 nmol/L: No  B-25OHD: 71 nmol/L  Δ 25OHD > 50%: No | 6 mo. of treatment (DB):  D3 2000 IU/d + Ca 1200 mg/d  Vs  Placebo | RR 3.00 (95% Cl, 0.13 to 70.02) |
| Grimnes, 2011  Grimnes 2011, 21911741, Norway |  |  |  |  |  |  |  |  | X |  |  |  | N= 104 (49% F)  Eldery healthy people with low vitamin D status  Mean age: 52 ±8.8 yr.  IC-25OHD $<$ 50 nmol/L: No  B-25OHD: 42.2nmol/L  Δ 25OHD > 50%: yes | For 6 months treatment (DB) with  D3 20,000 IU/twice weekly  Vs  Placebo | RR 0.35 (95 % Cl, 0.01, 8.31) |
| Alvarez, 2012 [63], USA |  |  |  |  | X |  |  |  |  |  |  |  | N = 46 (9% F) patients with early kidney disease.  Mean age: 62.4 yr.  IC-25OHD: <50 nmol/L: No  B-25OHD: 66.8 nmol/L  Δ 25OHD > 50%: No | 12 mo. of treatment (DB):  D2 50,000 IU/week  Vs.  placebo | RR 1.09 (95% Cl, 0.07 to 16.41) |
| Lehouck, 2012 [25], Belgium |  |  |  |  | X |  |  |  | X |  | X |  | N=182 (20% F) patients with moderate to severe chronic obstructive pulmonary disease  Mean age 68±9 yr.  IC-25OHD: <50 nmol/L: No  B-25OHD:50 nmol/L.  Δ 25OHD > 50%: Yes | 12 mo. of treatment (DB)  D3 100,000/mo.  Vs.  Placebo | RR 1.50 (95% Cl, 0.56 to 4.04) |
| Glendenning, 2012 [123], AU |  |  |  |  |  |  | X | X | X | X |  |  | N=686 elderly community-dwelling ambulatory women  Mean age 76.9 ±4.0  IC-25OHD: < 50 nmol/L: No  B-25OHD: 66±23 nmol/L (sub-group)  Δ 25OHD > 50%: No | 6 mo. of treatment (DB):  D3 150,000 IU/three monthly  Vs  Placebo | Follow up for 9 mo.:  RR 7.72 (95% Cl, 0.23 to 97.90) |
| Punthakee, 2012 [263], CA |  |  |  |  | X |  |  |  |  |  | X |  | N= 1221 (41 % F) patients with type 2 diabetes  Mean age: 66.6 ±6.3 yr.  IC-25OHD: < 50 nmol/L: No  B-25OHD: NR  Δ 25OHD > 50%: NR | 6 mo. of treatment (DB)  D3 1000 IU/d  Vs  Placebo | Follow up for 5.5 yr.  RR 0.20 (95% CI, 0.01 to 4.21) |
| Wasse, 2012 [264], USA |  |  |  |  | X |  |  |  |  |  |  |  | N=102 (38% F) patients on hemodialysis.  IC-25OHD: < 50 nmol/L: No  B-25OHD: 36 nmol/L  Δ 25OHD > 50%: Yes | 3 wks. of treatment (NB):  D3 200,000 IU/wk.  Vs  None | RR 0.2.16 (95% CI, 0.21 to 22.38) |
| Witham, 2013 [26], Scotland |  |  |  |  |  |  | X | X |  |  |  |  | N=159 (59% F) with isolated systolic hypertension aged >70 yr.  IC-25OHD: < 50 nmol/L: No  B-25OHD: 45±15 nmol/L  Δ 25OHD > 50%: Yes | Every 3 months for 1 year (DB):  D3 100,000 IU  Vs  Placebo | RR 0.99 (95% CI, 0.14 to 6.84) |

# Search string

For PubMed we used the search string ((vitamin D) AND ((("review"[Publication Type]) AND Systematic) OR ("Meta-analysis"[Publication Type]) AND ("2006/12/01"[Date - Publication] : "2016/11/30"[Date - Publication])))

For Embase we used: ['meta analysis' OR 'systematic review'/de] AND ['hypertension'/exp OR 'cardiovascular disease'/exp OR 'mortality'/exp OR 'respiratory tract infection'/exp OR 'depression'/exp OR 'diabetes'/exp OR 'cancer'/exp OR 'weight'/exp OR 'obesity'] AND ['vitamin d'/exp OR 'vitamin d supplementation' AND [english]/lim AND [1-12-2006]/sd NOT [30-11-2016]/sd]

For the Cochrane Library we used: ["vitamin D":ti,ab,kw] (Word variations have been searched) AND [Review] (publication type) with publication date between December 2006 and November 2016].

# References for the appendix

Reference List

1. Wang L, Manson JE, Song Y, Sesso HD. Systematic review: Vitamin D and calcium supplementation in prevention of cardiovascular events. Ann Intern Med 2010; 152: 315-323.

2. Trivedi DP, Doll R, Khaw KT. Effect of four monthly oral vitamin D3 (cholecalciferol) supplementation on fractures and mortality in men and women living in the community: randomised double blind controlled trial. BMJ 2003; 326: 469.

3. Prince RL, Austin N, Devine A, Dick IM, Bruce D, Zhu K. Effects of Ergocalciferol Added to Calcium on the Risk of Falls in Elderly High-Risk Women. Arch Intern Med 2008; 168: 103-108.

4. Brazier M, Grados F, Kamel S, Mathieu M, Morel A, Maamer M et al. Clinical and laboratory safety of one year's use of acombination calcium + vitamin D tablet in ambulatory elderly women with vitamin D insufficiency: Results of a mlticenter, rndomized, double-blind, placebo-controlled study. Clin Ther 2005; 27: 1885-1893.

5. Hsia J, Heiss G, Ren H, Allison M, Dolan NC, Greenland P et al. Calcium/vitamin D supplementation and cardiovascular events. Circulation 2007; 115: 846-854.

6. Elamin MB, bu Elnour NO, Elamin KB, Fatourechi MM, Alkatib AA, Almandoz JP et al. Vitamin D and cardiovascular outcomes: a systematic review and meta-analysis. J Clin Endocrinol Metab 2011; 96: 1931-1942.

7. Komulainen M, Kröger H, Tuppurainen MT, Heikkinen AM, Alhava E, Honkanen R et al. Prevention of femoral and lumbar bone loss with hormone replacement therapy and vitamin D3 in early postmenopausal women: a population-based 5-year randomized trial. J Clin Endocrinol Metab 1999; 84: 546-552.

8. Jackson RD, LaCroix AZ, Gass M, Wallace RB, Robbins J, Lewis CE et al. Calcium plus vitamin D supplementation and the risk of fractures. N Engl J Med 2006; 354: 669-683.

9. Berggren M, Stenvall M, Olofsson B, Gustafson Y. Evaluation of a fall-prevention program in older people after femoral neck fracture: a one-year follow-up. Osteoporos Int 2008; 19: 801-809.

10. Inkovaara J, Gothoni G, Halttula R, Heikinheimo R, Tokola O. Calcium, vitamin D and anabolic steroid in treatment of aged bones: double-blind placebo-controlled long-term clinical trial. Age Ageing 1983; 12: 124-130.

11. Myung S-K, Ju W, Cho B, Oh S-W, Park SM, Koo B-K et al. Efficacy of vitamin and antioxidant supplements in prevention of cardiovascular disease: systematic review and meta-analysis of randomised controlled trials. BMJ 2013; 346.

12. Mark SD, Wang W, Fraumeni JF, Li JY, Taylor PR, Wang GQ et al. Lowered Risks of Hypertension and Cerebrovascular Disease after Vitamin/Mineral Supplementation: The Linxian Nutrition Intervention Trial. Am J Epidemiol 1996; 143: 658-664.

13. Mao PJ, Zhang C, Tang L, Xian YQ, Li YS, Wang WD et al. Effect of calcium or vitamin D supplementation on vascular outcomes: A meta-analysis of randomized controlled trials. Int J Cardiol 2013; 169: 106-111.

14. Grant AM, Avenell A, Campbell MK, McDonald AM, MacLennan GS, McPherson GC et al. Oral vitamin D3 and calcium for secondary prevention of low-trauma fractures in elderly people (Randomised Evaluation of Calcium Or vitamin D, RECORD): a randomised placebo-controlled trial. Lancet 2005; 365: 1621-1628.

15. Lappe JM, Travers-Gustafson D, Davies KM, Recker RR, Heaney RP. Vitamin D and calcium supplementation reduces cancer risk: results of a randomized trial. Am J Clin Nutr 2007; 85: 1586-1591.

16. Fortmann SP, Burda BU, Senger CA, Lin JS, Whitlock EP. Vitamin and Mineral Supplements in the Primary Prevention of Cardiovascular Disease and Cancer: An Updated Systematic Evidence Review for the U.S. Preventive Services Task Force. Ann Intern Med 2013; 159: 824-834.

17. Ford JA, MacLennan GS, Avenell A, Bolland M, Grey A, Witham M et al. Cardiovascular disease and vitamin D supplementation: trial analysis, systematic review, and meta-analysis. Am J Clin Nutr 2014; 100: 746-755.

18. Wactawski-Wende J, Kotchen JM, Anderson GL, Assaf AR, Brunner RL, O'Sullivan MJ et al. Calcium plus Vitamin D Supplementation and the Risk of Colorectal Cancer. N Engl J Med 2006; 354: 684-696.

19. Aloia JF, Vaswani A, Yeh JK, Ellis K, Yasumura S, Cohn SH. Calcitriol in the treatment of postmenopausal osteoporosis. Am J Med 1988; 84: 401-408.

20. Ott SM, Chesnut III. Calcitriol Treatment Is Not Effective in Postmenopausal Osteoporosis. Ann Intern Med 1989; 110: 267-274.

21. Gallagher JC, Fowler SE, Detter JR, Sherman SS. Combination treatment with estrogen and calcitriol in the prevention of age-related bone loss. J Clin Endocrinol Metab 2001; 86: 3618-3628.

22. Zhu K, Devine A, Dick IM, Wilson SG, Prince RL. Effects of Calcium and Vitamin D Supplementation on Hip Bone Mineral Density and Calcium-Related Analytes in Elderly Ambulatory Australian Women: A Five-Year Randomized Controlled Trial. J Clin Endocrinol Metab 2008; 93: 743-749.

23. Xia Wb, Zhang Zl, Wang Hf, Meng Xw, Zhang Y, Zhu Gy et al. The efficacy and safety of calcitriol and/or Caltrate D in elderly Chinese women with low bone mass. Acta Pharmacol Sin 2009; 30: 372-378.

24. Sanders KM, Stuart AL, Williamson EJ, Jacka FN, Dodd S, Nicholson G et al. Annual high-dose vitamin D3 and mental well-being: randomised controlled trial. Br J Psychiatry 2011; 198: 357-364.

25. Lehouck A, Mathieu C, Carremans C, Baeke F, Verhaegen J, Van Eldere J et al. High Doses of Vitamin D to Reduce Exacerbations in Chronic Obstructive Pulmonary DiseaseA Randomized Trial. Ann Intern Med 2012; 156: 105-114.

26. Witham MD, Price RG, Struthers AD. Cholecalciferol treatment to reduce blood pressure in older patients with isolated systolic hypertension: The VitDISH randomized controlled trial. JAMA Internal Medicine 2013; 173: 1672-1679.

27. Avenell A, Grant AM, McGee M, McPherson G, Campbell MK, McGee MA et al. The effects of an open design on trial participant recruitment, compliance and retention - a randomized controlled trial comparison with a blinded, placebo-controlled design. Clinical Trials 2004; 1: 490-498.

28. Matsumoto T, Miki T, Hagino H, Sugimoto T, Okamoto S, Hirota T et al. A new active vitamin D, ED-71, increases bone mass in osteoporotic patients under vitamin D supplementation: a randomized, double-blind, placebo-controlled clinical trial. J Clin Endocrinol Metab 2005; 90: 5031-5036.

29. Gallagher JC, Sai A, Templin II, Smith L. Dose Response to Vitamin D Supplementation in Postmenopausal Women - A Randomized Trial. Ann Intern Med 2012; 156: 425-437.

30. Bolland MJ, Grey A, Gamble GD, Reid IR. The effect of vitamin D supplementation on skeletal, vascular, or cancer outcomes: a trial sequential meta-analysis. Lancet Diabetes Endocrinol 2014; 2: 307-320.

31. Chung M, Balk EM, Brendel M, Ip S, Lau J, Lee J et al. Vitamin D and calcium: a systematic review of health outcomes. Evid Rep Technol Assess (Full Rep ) 2009; 183: 1-420.

32. Chung M, Lee J, Terasawa T, Lau J, Trikalinos TA. Vitamin D With or Without Calcium Supplementation for Prevention of Cancer and Fractures: An Updated Meta-analysis for the U.S. Preventive Services Task Force. Ann Intern Med 2011; 155: 827-838.

33. Gallagher JC. The effects of calcitriol on falls and fractures and physical performance tests. J Steroid Biochem Mol Biol 2004; 89−90: 497-501.

34. Witham MD, Nadir MA, Struthers AD. Effect of vitamin D on blood pressure: a systematic review and meta-analysis. J Hypertens 2009; 27: 1948-1954.

35. Scragg R, Khaw KT, Murphy S. Effect of winter oral vitamin D3 supplementation on cardiovascular risk factors in elderly adults. Eur J Clin Nutr 1995; 49: 640-646.

36. Krause R, Buhring M, Hopfenmuller W, Holick MF, Sharma AM. Ultraviolet B and blood pressure. The Lancet 1998; 352: 709-710.

37. Pfeifer M, Begerow B, Minne HW, Nachtigall D, Hansen C. Effects of a Short-Term Vitamin D3 and Calcium Supplementation on Blood Pressure and Parathyroid Hormone Levels in Elderly Women. J Clin Endocrinol Metab 2001; 86: 1633-1637.

38. Sugden JA, Davies JI, Witham MD, Morris AD, Struthers AD. Vitamin-D improves endothelial function in patients with Type-2 diabetes mellitus and low vitamin-D levels. Diabet Med 2008; 25: 320-325.

39. Wu SH, Ho SC, Zhong L. Effects of vitamin D supplementation on blood pressure. South Med J 2010; 103: 729-737.

40. Pan WH, Wang CY, Li LA, Kao LS, Yeh SH. No significant effect of calcium and vitamin D supplementation on blood pressure and calcium metabolism in elderly Chinese. Chin J Physiol 1993; 36: 85-94.

41. Major GC, Alarie F, Dore J, Phouttama S, Tremblay A. Supplementation with calcium + vitamin D enhances the beneficial effect of weight loss on plasma lipid and lipoprotein concentrations. Am J Clin Nutr 2007; 85: 54-59.

42. Pittas AG, Chung M, Trikalinos T, Mitri J, Brendel M, Patel K et al. Systematic Review: Vitamin D and Cardiometabolic Outcomes. Ann Intern Med 2010; 152: 307-314.

43. Schleithoff SS, Zittermann A, Tenderich G, Berthold HK, Stehle P, Koerfer R. Vitamin D supplementation improves cytokine profiles in patients with congestive heart failure: a double-blind, randomized, placebo-controlled trial. Am J Clin Nutr 2006; 83: 754-759.

44. Margolis KL, Ray RM, Van Horn L, Manson JE, Allison MA, Black HR et al. Effect of Calcium and Vitamin D Supplementation on Blood Pressure: The Womens Health Initiative Randomized Trial. Hypertension 2008; 52: 847-855.

45. Nagpal J, Pande JN, Bhartia A. A double-blind, randomized, placebo-controlled trial of the short-term effect of vitamin D3 supplementation on insulin sensitivity in apparently healthy, middle-aged, centrally obese men. Diabet Med 2009; 26: 19-27.

46. Zittermann A, Frisch S, Berthold HK, Gõtting C, Kuhn J, Kleesiek K et al. Vitamin D supplementation enhances the beneficial effects of weight loss on cardiovascular disease risk markers. Am J Clin Nutr 2009; 89: 1321-1327.

47. Jorde R, Figenschau Y. Supplementation with cholecalciferol does not improve glycaemic control in diabetic subjects with normal serum 25-hydroxyvitamin D levels. Eur J Nutr 2009; 48: 349-354.

48. Kunutsor S, Burgess S, Munroe P, Khan H. Vitamin D and high blood pressure: causal association or epiphenomenon? Eur J Epidemiol 2014; 29: 1-14.

49. Beveridge LA, Struthers AD, Khan F, Jorde R, Scragg R, Macdonald HM et al. Effect of Vitamin D Supplementation on Blood Pressure: A Systematic Review and Meta-analysis Incorporating Individual Patient Data. JAMA 2015; 175: 745-754.

50. Manousopoulou A, Al-Daghri NM, Garbis SD, Chrousos GP. Vitamin D and cardiovascular risk among adults with obesity: a systematic review and meta-analysis. Eur J Clin Invest 2015; 45: 1113-1126.

51. Salehpour A, Hosseinpanah F, Shidfar F, Vafa M, Razaghi M, Dehghani S et al. A 12-week double-blind randomized clinical trial of vitamin D3 supplementation on body fat mass in healthy overweight and obese women. Nutrition Journal 2012; 11: 78.

52. Maki KC, Rubin MR, Wong LG, McManus JF, Jensen CD, Lawless A. Effects of vitamin D supplementation on 25-hydroxyvitamin D, high-density lipoprotein cholesterol, and other cardiovascular disease risk markers in subjects with elevated waist circumference. Int J Food Sci Nutr 2011; 62: 318-327.

53. Jorde R, Sneve M, Torjesen P, Figenschau Y. No improvement in cardiovascular risk factors in overweight and obese subjects after supplementation with vitamin D3 for 1-year. J Int Med 2010; 267: 462-472.

54. Golzarand M, Shab-Bidar S, Koochakpoor G, Speakman J, Djafarian K. Effect of vitamin D3 supplementation on blood pressure in adults: An updated meta-analysis. Nutr Metab Cardiovasc Dis 2016; 26: 663-673.

55. Lee KJ, Lee YJ. Effects of vitamin D on blood pressure in patients with type 2 diabetes mellitus. Int J Clin Pharmacol Ther 2016; 54: 233-242.

56. de Zeeuw D, Agarwal R, Amdahl M, Audhya P, Coyne D, Garimella T et al. Selective vitamin D receptor activation with paricalcitol for reduction of albuminuria in patients with type 2 diabetes (VITAL study): a randomised controlled trial. The Lancet 2006; 376: 1543-1551.

57. Witham MD, Dove FJ, Dryburgh M, Sugden JA, Morris AD, Struthers AD. The effect of different doses of vitamin D3 on markers of vascular health in patients with type 2 diabetes: a randomised controlled trial. Diabetologia 2010; 53: 2112-2119.

58. Witham MD, Crighton LJ, Gillespie ND, Struthers AD, McMurdo MET. The Effects of Vitamin D Supplementation on Physical Function and Quality of Life in Older Patients With Heart Failure: A Randomized Controlled Trial. Circ Heart Fail 2010; 3: 195-201.

59. Nikooyeh B, Neyestani TR, Farvid M, avi-Majd H, Houshiarrad A, Kalayi A et al. Daily consumption of vitamin D- or vitamin D + calcium-fortified yogurt drink improved glycemic control in patients with type 2 diabetes: a randomized clinical trial. Am J Clin Nutr 2011; 93: 764-771.

60. Harris RA, Pedersen-White J, Guo DH, Stallmann-Jorgensen IS, Keeton D, Huang Y et al. Vitamin D3 Supplementation for 16 Weeks Improves Flow-Mediated Dilation in Overweight African-American Adults. Am J Hypertens 2011; 24: 557-562.

61. Shab-Bidar S, Neyestani T, Djazayery A, Eshraghian MR, Houshiarrad A, Gharavi A' et al. Regular consumption of vitamin D-fortified yogurt drink (Doogh) improved endothelial biomarkers in subjects with type 2 diabetes: a randomized double-blind clinical trial. BMC Medicine 2011; 9: 125.

62. Larsen T, Mose FH, Bech JN, Hansen AB, Pedersen EB. Effect of Cholecalciferol Supplementation During Winter Months in Patients With Hypertension: A Randomized, Placebo-Controlled Trial. Am J Hypertens 2012; 25: 1215-1222.

63. Alvarez JA, Law J, Coakley KE, Zughaier SM, Hao L, Shahid Salles K et al. High-dose cholecalciferol reduces parathyroid hormone in patients with early chronic kidney disease: a pilot, randomized, double-blind, placebo-controlled trial. Am J Clin Nutr 2012; 96: 672-679.

64. Gepner AD, Ramamurthy R, Krueger DC, Korcarz CE, Binkley N, Stein JH. A prospective randomized controlled trial of the effects of vitamin D supplementation on cardiovascular disease risk. PLoS ONE 2012; 7: e36617.

65. Ahmadi N, Mortazavi M, Iraj B, Askari G. Whether vitamin D3 is effective in reducing proteinuria in type 2 diabetic patients? J Res Med Sci 2013; 18: 374-377.

66. Longenecker CT, Hileman CO, Carman TL, Ross AC, Seydafkan S, Brown TT et al. Vitamin D supplementation and endothelial function in vitamin D deficient HIV-infected patients: A randomized placebo-controlled trial. Antivir Ther 2012; 17: 613-621.

67. Heshmat R, Tabatabaei-Malazy O, Abbaszadeh-Ahranjani S, Shahbazi S, Khooshehchin G, Bandarian F et al. Effect of vitamin D on insulin resistance and anthropometric parameters in Type 2 diabetes; a randomized double-blind clinical trial. DARU 2012; 20: 10.

68. Kjærgaard M, Waterloo K, Wang CEA, Almås B, Figenschau Y, Hutchinson MS et al. Effect of vitamin D supplement on depression scores in people with low levels of serum 25-hydroxyvitamin D: nested case-control study and randomised clinical trial. Br J Psychiatry 2012; 201: 360-368.

69. Muldowney S, Lucey AJ, Hill TR, Seamans KM, Taylor N, Wallace JMW et al. Incremental Cholecalciferol Supplementation up to 15 μg/d Throughout Winter at 51-55^o^ N Has No Effect on Biomarkers of Cardiovascular Risk in Healthy Young and Older Adults. J Nutr 2012; 142: 1519-1525.

70. Salehpour A, Shidfar F, Hosseinpanah F, Vafa M, Razaghi M, Hoshiarrad A et al. Vitamin D3 and the risk of CVD in overweight and obese women: a randomised controlled trial. Br J Nutr 2012; 108: 1866-1873.

71. Stricker H, Tosi Bianda F, Guidicelli-Nicolosi S, Limoni C, Colucci G Effect of a Single, Oral, High-dose Vitamin D Supplementation on Endothelial Function in Patients with Peripheral Arterial Disease: A Randomised Controlled Pilot Study. (2012) Eur J Vasc Endovasc Surg 44: 307-312.

72. Witham MD, Dove FJ, Sugden JA, Doney AS, Struthers AD. The effect of vitamin D replacement on markers of vascular health in stroke patients - A randomised controlled trial. Nutrition, Metabolism and Cardiovascular Diseases 2012; 22: 864-870.

73. Wood AD, Secombes KR, Thies F, Aucott L, Black AJ, Mavroeidi A et al. Vitamin D3 Supplementation Has No Effect on Conventional Cardiovascular Risk Factors. A Parallel-Group, Double-Blind, Placebo-Controlled RCT. J Clin Endocrin Metab 2012; 97: 3557-3568.

74. Asemi Z, Hashemi T, Karamali M, Samimi M, Esmaillzadeh A. Effects of vitamin D supplementation on glucose metabolism, lipid concentrations, inflammation, and oxidative stress in gestational diabetes: a double-blind randomized controlled clinical trial. Am J Clin Nutr 2013; 98: 1425-1432.

75. Boxer RS, Kenny AM, Schmotzer BJ, Vest M, Fiutem JJ, Pina IL. A Randomized Controlled Trial of High-Dose Vitamin D3 in Patients With Heart Failure. JACC: Heart Failure 2013; 1: 84-90.

76. Breslavsky A, Frand J, Matas Z, Boaz M, Barnea Z, Shargorodsky M. Effect of high doses of vitamin D on arterial properties, adiponectin, leptin and glucose homeostasis in type 2 diabetic patients. Clin Nutr 2013; 32: 970-975.

77. Breslavsky A, Oz H, Matas Z, Shargorodsky M. The effect of vitamin D supplementation on plasma leptin/adiponectin ratio in diabetic individuals with different haptoglobin phenotypes. Cardiovasc Endocrinol 2014; 3: 74-78.

78. Chai W, Cooney RV, Franke AA, Bostick RM. Effects of calcium and vitamin D supplementation on blood pressure and serum-álipids and carotenoids: a randomized, double-blind, placebo-controlled, clinical-átrial. Ann Epidemiol 2013; 23: 564-570.

79. Forman JP, Scott JB, Ng K, Drake BF, Suarez EG, Hayden DL et al. Effect of Vitamin D Supplementation on Blood Pressure in Blacks. Hypertension 2013; 61: 779-785.

80. Petchey WG, Hickman IJ, Prins JB, Hawley CM, Johnson DW, Isbel NM. Vitamin D does not improve the metabolic health of patients with chronic kidney disease stage 3-4: A randomized controlled trial. Nephrology 2013; 18: 26-35.

81. Roth D, Al Mahmud A, Raqib R, Akhtar E, Perumal N, Pezzack B et al. Randomized placebo-controlled trial of high-dose prenatal third-trimester vitamin D3 supplementation in Bangladesh: the AViDD trial. Nutrition Journal 2013; 12: 47.

82. Toxqui L, Blanco-Rojo R, Wright I, P+®rez-Granados MA, Vaquero PM. Changes in Blood Pressure and Lipid Levels in Young Women Consuming a Vitamin D-Fortified Skimmed Milk: A Randomised Controlled Trial. Nutrients 2013; 5: 4966-4977.

83. Wamberg L, Kampmann U, Stødkilde-Jørgensen H, Rejnmark L, Pedersen SB, Richelsen B. Effects of vitamin D supplementation on body fat accumulation, inflammation, and metabolic risk factors in obese adults with low vitamin D levels - Results from a randomized trial. Eur J Intern Med 2013; 24: 644-649.

84. Witham MD, Dove FJ, Khan F, Lang CC, Belch JJF, Struthers AD. Effects of Vitamin D supplementation on markers of vascular function after myocardial infarction − A randomised controlled trial. Int J Cardiol 2013; 167: 745-749.

85. Witham MD, Adams F, Kabir G, Kennedy G, Belch JJF, Khan F. Effect of short-term vitamin D supplementation on markers of vascular health in South Asian women living in the UK - A randomised controlled trial. Atherosclerosis 2013; 230: 293-299.

86. Yiu YF, Yiu KH, Siu CW, Chan YH, Li SW, Wong LY et al. Randomized controlled trial of vitamin D supplement on endothelial function in patients with type 2 diabetes. Atherosclerosis 2013; 227: 140-146.

87. Moghassemi S, Marjani A. The effect of short-term vitamin D supplementation on lipid profile and blood pressure in post-menopausal women: A randomized controlled trial. Iran J Nurs Midwifery Res 2014; 5: 517-521.

88. Dalbeni A, Scaturro G, Degan M, Minuz P, Delva P. Effects of six months of vitamin D supplementation in patients with heart failure: A randomized double-blind controlled trial. Nutr Metab Cardiovasc Dis 2014; 24: 861-868.

89. Scragg R, Slow S, Stewart AW, Jennings LC, Chambers ST, Priest PC et al. Long-Term High-Dose Vitamin D_3_ Supplementation and Blood Pressure in Healthy Adults. Hypertension 2014; 64: 725.

90. Sollid ST, Hutchinson MYS, Fuskev+Ñg OM, Figenschau Y, Joakimsen RM, Schirmer H et al. No Effect of High-Dose Vitamin D Supplementation on Glycemic Status or Cardiovascular Risk Factors in Subjects With Prediabetes. Diabetes Care 2014; 37: 2123.

91. Strobel F, Reusch J, Penna-Martinez M, Ramos-Lopez E, Klahold E, Klepzig C et al. Effect of a Randomised Controlled Vitamin D Trial on Insulin Resistance and Glucose Metabolism in Patients with Type 2 Diabetes Mellitus. Horm Metab Res 2014; 46: 54-58.

92. Raja-Khan N, Shah J, Stetter CM, Lott MEJ, Kunselman AR, Dodson WC et al. High-dose vitamin D supplementation and measures of insulin sensitivity in polycystic ovary syndrome: a randomized, controlled pilot trial. Fertility and Sterility 2014; 101: 1740-1746.

93. Chen WR, Liu ZY, Shi Y, Yin DW, Wang H, Sha Y et al. Vitamin D and nifedipine in the treatment of Chinese patients with grades I-II essential hypertension: A randomized placebo-controlled trial. Atherosclerosis 2014; 235: 102-109.

94. Witham MD, Ireland S, Houston JG, Gandy SJ, Waugh S, MacDonald TM et al. Vitamin D Therapy to Reduce Blood Pressure and Left Ventricular Hypertrophy in Resistant Hypertension: Randomized, Controlled Trial. Hypertension 2014; 63: 706-712.

95. Ryu OH, Chung W, Lee S, Hong KS, Choi MG, Yoo HJ. The effect of high-dose vitamin D supplementation on insulin resistance and arterial stiffness in patients with type 2 diabetes. Korean J Intern Med 2014; 29: 620-629.

96. Jehle S, Lardi AF, Felix BF, Hulter HN FAU - Stettler C, Stettler CF, Krapf R. Effect of large doses of parenteral vitamin D on glycaemic control and calcium/phosphate metabolism in patients with stable type 2 diabetes mellitus: a randomised, placebo-controlled, prospective pilot study. Swiss Med Wkly 2014; 144: w13942.

97. Kampmann U, Mosekilde L, Juhl C, Moller N, Christensen B, Rejnmark L et al. Effects of 12 weeks high dose vitamin D3 treatment on insulin sensitivity, beta cell function, and metabolic markers in patients with type 2 diabetes and vitamin D insufficiency - a double-blind, randomized, placebo-controlled trial. Metabolism 2014; 63: 1115-1124.

98. Nasri H, Behradmanesh S, Ahmadi A, Rafieian-Kopaei M. Impact of oral vitamin D (cholecalciferol) replacement therapy on blood pressure in type 2 diabetes patients; a randomized, double-blind, placebo controlled clinical trial. J Nephropathol 2014; 3: 29-33.

99. Tabesh M, Azadbakht L, Faghihimani E, Tabesh M, Esmaillzadeh A. Effects of calcium-vitamin D co-supplementation on metabolic profiles in vitamin D insufficient people with type 2 diabetes: a randomised controlled clinical trial. Diabetologia 2014; 57: 2038-2047.

100. Tabesh M, Azadbakht L, Faghihimani E, Tabesh M, Esmaillzadeh A. Effects of Calcium Plus Vitamin D Supplementation on Anthropometric Measurements and Blood Pressure in Vitamin D Insufficient People with Type 2 Diabetes: A Randomized Controlled Clinical Trial. J Am Coll Nutr 2015; 34: 281-289.

101. Sadiya A, Ahmed SM, Carlsson M, Tesfa Y, George M, Ali SH et al. Vitamin D supplementation in obese type 2 diabetes subjects in Ajman, UAE: a randomized controlled double-blinded clinical trial. Eur J Clin Nutr 2015; 69: 707-711.

102. Pilz S, Gaksch M, Kienreich K, Grübler M, Verheyen N, Fahrleitner-Pammer A et al. Effects of Vitamin D on Blood Pressure and Cardiovascular Risk Factors: A Randomized Controlled Trial. Hypertension 2015; 65: 1195.

103. Mozaffari-Khosravi H, Loloei S, Mirjalili MR, Barzegar K. The effect of vitamin D supplementation on blood pressure in patients with elevated blood pressure and vitamin D deficiency: a randomized, double-blind, placebo-controlled trial. Blood Pressure Monitoring 2015; 20: 83-91.

104. Seida JC, Mitri J, Colmers IN, Majumdar SR, Davidson MB, Edwards AL et al. Effect of Vitamin D Supplementation on Improving Glucose Homeostasis and Preventing Diabetes: A Systematic Review and Meta-Analysis. J Clin Endocrinol Metab 2014; 99: 3551-3560.

105. Pittas AG, Harris SS, Stark PC, Dawson-Hughes B. The Effects of Calcium and Vitamin D Supplementation on Blood Glucose and Markers of Inflammation in Nondiabetic Adults. Diabetes Care 2007; 30: 980-986.

106. de Boer IH, Tinker LF, Connelly S, Curb JD, Howard BV, Kestenbaum B et al. Calcium plus vitamin D supplementation and the risk of incident diabetes in the Women's Health Initiative. Diabetes Care 2008; 31: 701-707.

107. Avenell A, Cook JA, MacLennan GS, McPherson GC. Vitamin D supplementation and type 2 diabetes: a substudy of a randomised placebo-controlled trial in older people (RECORD trial). Age Ageing 2009; 38: 606-609.

108. Davidson MB, Duran P, Lee ML, Friedman TC. High-Dose Vitamin D Supplementation in People With Prediabetes and Hypovitaminosis D. Diabetes Care 2013; 36: 260-266.

109. Pathak K, Soares MJ, Calton EK, Zhao Y, Hallett J. Vitamin D supplementation and body weight status: a systematic review and meta-analysis of randomized controlled trials. Obes Rev 2014; 15: 528-537.

110. Sneve M, Figenschau Y, Jorde R. Supplementation with cholecalciferol does not result in weight reduction in overweight and obese subjects. Eur J Endocrinol 2008; 159: 675-684.

111. von Hurst PR, Stonehouse W, Coad J. Vitamin D supplementation reduces insulin resistance in South Asian women living in New Zealand who are insulin resistant and vitamin D deficient - a randomised, placebo-controlled trial. Br J Nutr 2010; 103: 549-555.

112. Grimnes G, Figenschau Y, Almås B, Jorde R. Vitamin D, Insulin Secretion, Sensitivity, and Lipids: Results From a Case-Control Study and a Randomized Controlled Trial Using Hyperglycemic Clamp Technique. Diabetes 2011; 60: 2748-2757.

113. Ardabili HR, Gargari BP, Farzadi L. Vitamin D supplementation has no effect on insulin resistance assessment in women with polycystic ovary syndrome and vitamin D deficiency. Nutrition Research 2012; 32: 195-201.

114. Carrillo AE, Flynn MG, Pinkston C, Markofski MM, Jiang Y, Donkin SS et al. Impact of vitamin D supplementation during a resistance training intervention on body composition, muscle function, and glucose tolerance in overweight and-áobese adults. Clin Nutr 2013; 32: 375-381.

115. Chandler PD, Wang L, Zhang X, Sesso HD, Moorthy MV, Obi O et al. Effect of vitamin D supplementation alone or with calcium on adiposity measures: a systematic review and meta-analysis of randomized controlled trials. Nutr Rev 2015; 73: 577.

116. Ljunghall S, Lind L, Lithell H, Skarfors E, Selinus I, Sorensen OH et al. Treatment with one-alpha-hydroxycholecalciferol in middle-aged men with impaired glucose tolerance--a prospective randomized double-blind study. Acta Med Scand 1987; 222: 361-367.

117. Dawson-Hughes B, Dallal GE, Krall EA, Harris S, Sokoll LJ, Falconer G. Effect of Vitamin D Supplementation on Wintertime and Overall Bone Loss in Healthy Postmenopausal Women. Ann Intern Med 1991; 115: 505-512.

118. Heikkinen AM, Tuppurainen MT, Niskanen L, Komulainen M, Penttila I, Saarikoski S. Long-term vitamin D3 supplementation may have adverse effects on serum lipids during postmenopausal hormone replacement therapy. Eur J Endocrinol 1997; 137: 495-502.

119. Dawson-Hughes B, Harris SS, Krall EA, Dallal GE. Effect of calcium and vitamin D supplementation on bone density in men and women 65 years of age or older. N Engl J Med 1997; 337: 670-676.

120. Aloia JF, Talwar SA, Pollack S, Yeh J. A Randomized Controlled Trial of Vitamin D3 Supplementation in African American Women. Arch Intern Med 2005; 165: 1618-1623.

121. Caan B, Neuhouser M, Aragaki A, Lewis CB, Jackson R, Leboff MS et al. Calcium plus vitamin D supplementation and the risk of postmenopausal weight gain. Arch Intern Med 2007; 167: 893-902.

122. Zhou J, Zhao LJ, Watson P, Zhang Q, Lappe JM. The effect of calcium and vitamin D supplementation on obesity in postmenopausal women: secondary analysis for a large-scale, placebo controlled, double-blind, 4-year longitudinal clinical trial. Nutrition & Metabolism 2010; 7: 62.

123. Glendenning P, Zhu K, Inderjeeth C, Howat P, Lewis JR, Prince RL. Effects of three-monthly oral 150,000 IU cholecalciferol supplementation on falls, mobility, and muscle strength in older postmenopausal women: A randomized controlled trial. J Bone Min Res 2012; 27: 170-176.

124. Chandler PD, Scott JB, Drake BF, Ng K, Manson JE, Rifai N et al. Impact of Vitamin D Supplementation on Inflammatory Markers in African Americans: Results of a Four-Arm, Randomized, Placebo-Controlled Trial. Cancer Prev Res 2014; 7: 218.

125. Mason C, Xiao L, Imayama I, Duggan C, Wang CY, Korde L et al. Vitamin D3 supplementation during weight loss: a double-blind randomized controlled trial. Am J Clin Nutr 2014; 99: 1015-1025.

126. Mahomed K, Gulmezoglu AM. Vitamin D supplementation in pregnancy. Cochrane Database Syst Rev 2000; CD000228.

127. Brooke OG, Brown IR, Bone CD, Carter ND, Cleeve HJ, Maxwell JD et al. Vitamin D supplements in pregnant Asian women: effects on calcium status and fetal growth. Br Med J 1980; 280: 751-754.

128. Mallet E, Gugi B, Brunelle P, Henocq A, Basuyau JP, Lemeur H. Vitamin D supplementation in pregnancy: a controlled trial of two methods. Obstet Gynecol 1986; 68: 300-304.

129. Thorne-Lyman A, Fawzi WW. Vitamin D During Pregnancy and Maternal, Neonatal and Infant Health Outcomes: A Systematic Review and Meta-analysis. Paediatric and Perinatal Epidemiology 2012; 26: 75-90.

130. Marya RK, Rathee S, Lata V, Mudgil S. Effects of Vitamin D Supplementation in Pregnancy. Gynecol Obstet Invest 1981; 12: 155-161.

131. Yu CK, Sykes L, Sethi M, Teoh TG, Robinson S. Vitamin D deficiency and supplementation during pregnancy. Clin Endocrinol (Oxf) 2009; 70: 685-690.

132. Hollis BW, Johnson D, Hulsey TC, Ebeling M, Wagner CL. Vitamin D supplementation during pregnancy: Double blind, randomized clinical trial of safety and effectiveness. J Bone Min Res 2011; 26: 2341-2357.

133. De-Regil LM, Palacios C, Ansary A, Kulier R, Pena-Rosas JP. Vitamin D supplementation for women during pregnancy. Cochrane Database Syst Rev 2012; CD008873.

134. Harvey NC, Holroyd C, Ntani G, Javaid K, Cooper P, Moon R et al. Vitamin D supplementation in pregnancy: A systematic review. Health Technol Assess 2014; 18: 1-190.

135. Congdon P, Horsman A, Kirby PA, Dibble J, Bashir T. Mineral content of the forearms of babies born to Asian and white mothers. Br Med J (Clin Res Ed) 1983; 286: 1233-1235.

136. Kaur J, Marya RK, Rathee S, Lal H, Singh GP. Effect of pharmacological doses of vitamin D during pregnancy on placental protein status and birth weight. Nutr Res 1991; 11: 1077-1081.

137. Marya RK, Rathee S, Dua V, Sangwan K. Effect of vitamin D supplementation during pregnancy on foetal growth. Indian J Med Res 1988; 88: 488-492.

138. Pérez-López FR, Pasupuleti V, Mezones-Holguin E, Benites-Zapata VA, Thota P, Deshpande A et al. Effect of vitamin D supplementation during pregnancy on maternal and neonatal outcomes: a systematic review and meta-analysis of randomized controlled trials. Fertility and Sterility 2015; 103: 1278-1288.

139. Sabet Z, Ghazi AA, Tohidi M, Oladi B. Vitamin D Supplementation in Pregnant Iranian Women: Effects on Maternal and Neonatal Vitamin D and Parathyroid Hormone Status. Acta Endo (Buc) 2012; 8: 59-66.

140. Goldring ST, Griffiths CJ, Martineau AR, Robinson S, Yu C, Poulton S et al. Prenatal Vitamin D Supplementation and Child Respiratory Health: A Randomised Controlled Trial. PLoS ONE 2013; 8: e66627.

141. Hashemipour S, Ziaee A, Javadi A, Movahed F, Elmizadeh K, Javadi EH et al. Effect of treatment of vitamin D deficiency and insufficiency during pregnancy on fetal growth indices and maternal weight gain: a randomized clinical trial. Eur J Obstet Gynecol Reprod Biol 2014; 172: 15-19.

142. Hossain N, Kanani FH, Ramzan S, Kausar R, Ayaz S, Khanani R et al. Obstetric and neonatal outcomes of maternal vitamin D supplementation: results of an open-label, randomized controlled trial of antenatal vitamin D supplementation in Pakistani women. J Clin Endocrinol Metab 2014; 99: 2448-2455.

143. De-Regil LM, Palacios C, Lombardo LK, Pe+¦a-Rosas JP. Vitamin D supplementation for women during pregnancy. Cochrane Database of Systematic Reviews 2016; CD008873.

144. Sablok A, Batra A, Thariani K, Batra A, Bharti R, Aggarwal AR et al. Supplementation of vitamin D in pregnancy and its correlation with feto-maternal outcome. Clin Endocrinol 2015; 83: 536-541.

145. Avenell A, MacLennan GS, Jenkinson DJ, McPherson GC, McDonald AM, Pant PR et al. Long-Term Follow-Up for Mortality and Cancer in a Randomized Placebo-Controlled Trial of Vitamin D3 and/or Calcium (RECORD Trial). J Clin Endocrin Metab 2012; 97: 614-622.

146. Bjelakovic G, Gluud LL, Nikolova D, Whitfield K, Krstic G, Wetterslev J et al. Vitamin D supplementation for prevention of cancer in adults. Cochrane Database Syst Rev 2014; 6: CD007469.

147. Grady D, Halloran B, Cummings S, Leveille S, Wells L, Black D et al. 1,25-Dihydroxyvitamin D3 and muscle strength in the elderly: a randomized controlled trial. J Clin Endocrinol Metab 1991; 73: 1111-1117.

148. Bolton-Smith C, McMurdo ME, Paterson CR, Mole PA, Harvey JM, Fenton ST et al. Two-Year Randomized Controlled Trial of Vitamin K1 (Phylloquinone) and Vitamin D3 Plus Calcium on the Bone Health of Older Women. J Bone Min Res 2007; 22: 509-519.

149. Daly RM, Petrass N, Bass S, Nowson CA. The skeletal benefits of calcium- and vitamin D3-fortified milk are sustained in older men after withdrawal of supplementation: an 18-mo follow-up study. Am J Clin Nutr 2008; 87: 771-777.

150. Janssen HC, Samson MM, Verhaar HJ. Muscle strength and mobility in vitamin D-insufficient female geriatric patients: a randomized controlled trial on vitamin D and calcium supplementation. Aging Clin Exp Res 2010; 22: 78-84.

151. Sanders KM, Stuart AL, Williamson EJ, Simpson JA, Kotowicz MA, Young D et al. Annual High-Dose Oral Vitamin D and Falls and Fractures in Older Women: A Randomized Controlled Trial. JAMA 2010; 303: 1815-1822.

152. Brunner RL, Wactawski-Wende J, Caan BJ, Cochrane BB, Chlebowski RT, Gass MLS et al. The Effect of Calcium plus Vitamin D on Risk for Invasive Cancer: Results of the Women's Health Initiative (WHI) Calcium Plus Vitamin D Randomized Clinical Trial. Nutrition and Cancer 2011; 63: 827-841.

153. Murdoch DR, Slow S, Chambers ST. Effect of vitamin d3 supplementation on upper respiratory tract infections in healthy adults: The vidaris randomized controlled trial. JAMA 2012; 308: 1333-1339.

154. Sperati F, Vici P, Maugeri-Sacc+á M, Stranges S, Santesso N, Mariani L et al. Vitamin D Supplementation and Breast Cancer Prevention: A Systematic Review and Meta-Analysis of Randomized Clinical Trials. PLoS ONE 2013; 8: e69269.

155. Lazzeroni M, Serrano D, Pilz S, Gandini S. Vitamin D supplementation and cancer: review of randomized controlled trials. Anticancer Agents Med Chem 2013; 13: 118-125.

156. Keum N, Giovannucci E. Vitamin D supplements and cancer incidence and mortality: a meta-analysis. Br J Cancer 2014; 111: 976-980.

157. Heikkinen AM, Parviainen MT, Tuppurainen MT, Niskanen L, Komulainen MH, Saarikoski S. Effects of Postmenopausal Hormone Replacement Therapy With and Without Vitamin D3 on Circulating Levels of 25-Hydroxyvitamin D and 1,25-Dihydroxyvitamin D. Calcif Tissue Int 1998; 62: 26-30.

158. Chlebowski RT, Johnson KC, Kooperberg C, Pettinger M, Wactawski-Wende J, Rohan T et al. Calcium Plus Vitamin D Supplementation and the Risk of Breast Cancer. J Natl Cancer Inst 2008; 100: 1581-1591.

159. Autier P, Boniol M, Pizot C+, Mullie P. Vitamin D status and ill health: a systematic review. The Lancet Diabetes & Endocrinology 2014; 2: 76-89.

160. Daly RM, Brown M, Bass S, Kukuljan S, Nowson C. Calcium- and Vitamin D3-Fortified Milk Reduces Bone Loss at Clinically Relevant Skeletal Sites in Older Men: A 2-Year Randomized Controlled Trial. J Bone Min Res 2006; 21: 397-405.

161. Charan J, Goyal JP, Saxena D, Yadav P. Vitamin D for prevention of respiratory tract infections: A systematic review and meta-analysis. J Pharmacol Pharmacother 2012; 3: 300-303.

162. Aloia JF, Li-Ng M. Re: epidemic influenza and vitamin D. Epidemiol Infect 2007; 135: 1095-1096.

163. Li-Ng M, Aloia JF, Pollack S, CUNHA BA, MIKHAIL M, Yeh J et al. A randomized controlled trial of vitamin D3 supplementation for the prevention of symptomatic upper respiratory tract infections. Epidemiol Infect 2009; 137: 1396-1404.

164. Laaksi I, Ruohola J-P, Mattila V, Auvinen A, Ylikomi T, Pihlajamâki H. Vitamin D Supplementation for the Prevention of Acute Respiratory Tract Infection: A Randomized, Double-Blinded Trial among Young Finnish Men. J Infect Dis 2010; 202: 809-814.

165. Urashima M, Segawa T, Okazaki M, Kurihara M, Wada Y, Ida H. Randomized trial of vitamin D supplementation to prevent seasonal influenza A in schoolchildren. Am J Clin Nutr 2010; 91: 1255-1260.

166. Manaseki-Holland S, Qader G, Isaq Masher M, Bruce J, Zulf Mughal M, Chandramohan D et al. Effects of vitamin D supplementation to children diagnosed with pneumonia in Kabul: a randomised controlled trial. Trop Med Int Health 2010; 15: 1148-1155.

167. Bergman P, Lindh ÅU, Björkhem-Bergman L, Lindh JD. Vitamin D and Respiratory Tract Infections: A Systematic Review and Meta-Analysis of Randomized Controlled Trials. PLoS ONE 2013; 8: e65835.

168. Majak P, Olszowiec-Chlebna M, Smejda K, Stelmach I. Vitamin D supplementation in children may prevent asthma exacerbation triggered by acute respiratory infection. J Allergy Clin Immunol 2011; 127: 1294-1296.

169. Jorde R, Witham M, Janssens W, Rolighed L, Borchhardt K, de Boer IH et al. Vitamin D supplementation did not prevent influenza-like illness as diagnosed retrospectively by questionnaires in subjects participating in randomized clinical trials. Scand J Infect Dis 2011; 44: 126-132.

170. Camargo CA, Ganmaa D, Frazier AL, Kirchberg FF, Stuart JJ, Kleinman K et al. Randomized Trial of Vitamin D Supplementation and Risk of Acute Respiratory Infection in Mongolia. Pediatrics 2012; 130: e561-e567.

171. Manaseki-Holland S, Maroof Z, Bruce J, Mughal MZ, Masher MI, Bhutta ZA et al. Effect on the incidence of pneumonia of vitamin D supplementation by quarterly bolus dose to infants in Kabul: a randomised controlled superiority trial. Lancet 2012; 379: 1419-1427.

172. Bergman P, Norlin AC, Hansen S, Rekha RS, Agerberth B, Björkhem-Bergman L et al. Vitamin D3 supplementation in patients with frequent respiratory tract infections: a randomised and double-blind intervention study. BMJ Open 2012; 2: e001663.

173. Mao S, Huang S. Vitamin D supplementation and risk of respiratory tract infections: A meta-analysis of randomized controlled trials. Scand J Infect Dis 2013; 45: 696-702.

174. Jadad AR, Moore RA, Carroll D, Jenkinson C, Reynolds DJ, Gavaghan DJ et al. Assessing the quality of reports of randomized clinical trials: Is blinding necessary? Control Clin Trials 1996; 17: 1-12.

175. Xiao L, Xing C, Yang Z, Xu S, Wang M, Du H et al. Vitamin D supplementation for the prevention of childhood acute respiratory infections: a systematic review of randomised controlled trials. Br J Nutr 2015; 114: 1026-1034.

176. Yakoob MY, Salam RA, Khan FR, Bhutta ZA. Vitamin D supplementation for preventing infections in children under five years of age. Cochrane Database of Systematic Reviews 2016; CD008824.

177. Alonso A, Rodriguez J, Carvajal I, Prieto ML, Rodriguez RM, Pérez AM et al. Prophylactic vitamin D in healthy infants: assessing the need. Metabolism 2011; 60: 1719-1725.

178. Vuichard Gysin D, Dao D, Gysin CM, Lytvyn L, Loeb M. Effect of Vitamin D3 Supplementation on Respiratory Tract Infections in Healthy Individuals: A Systematic Review and Meta-Analysis of Randomized Controlled Trials. PLoS ONE 2016; 11: e0162996.

179. de Gruijl FR, Pavel S. The effects of a mid-winter 8-week course of sub-sunburn sunbed exposures on tanning, vitamin D status and colds. Photochem Photobiol Sci 2012; 11: 1848-1854.

180. Rees JR, Hendricks K, Barry EL, Peacock JL, Mott LA, Sandler RS et al. Vitamin D3 Supplementation and Upper Respiratory Tract Infections in a Randomized, Controlled Trial. Clinical Infectious Diseases 2013; 57: 1384-1392.

181. Tran B, Armstrong BK, Ebeling PR, English DR, Kimlin MG, van der Pols JC et al. Effect of vitamin D supplementation on antibiotic use: a randomized controlled trial. Am J Clin Nutr 2014; 99: 156-161.

182. Goodall E, Granados A, Luinstra K, Pullenayegum E, Coleman B, Loeb M et al. Vitamin D3 and gargling for the prevention of upper respiratory tract infections: a randomized controlled trial. BMC Infectious Diseases 2014; 14: 273.

183. Urashima M, Mezawa H, Noya M, Camargo CA. Effects of vitamin D supplements on influenza A illness during the 2009 H1N1 pandemic: a randomized controlled trial. Food Funct 2014; 5: 2365-2370.

184. Dubnov-Raz G, Rinat B, Hemilae H, Choleva L, Cohen A, Constantini N. Vitamin D Supplementation and Upper Respiratory Tract Infections in Adolescent Swimmers: A Randomized Controlled Trial. Pediatr Exerc Sci 2015; 27: 113-119.

185. Grant CC, Kaur S, Waymouth E, Mitchell EA, Scragg R, Ekeroma A et al. Reduced primary care respiratory infection visits following pregnancy and infancy vitamin D supplementation: a randomised controlled trial. Acta Paediatr 2015; 104: 396-404.

186. Simpson S, van der Mei I, Stewart N, Blizzard L, Tettey P, Taylor B. Weekly cholecalciferol supplementation results in significant reductions in infection risk among the vitamin D deficient: results from the CIPRIS pilot RCT. BMC Nutrition 2015; 1: 7.

187. Martineau AR, Jolliffe DA, Hooper RL, Greenberg L, Aloia JF, Bergman P et al. Vitamin D supplementation to prevent acute respiratory tract infections: systematic review and meta-analysis of individual participant data. BMJ 2017; 356.

188. Kumar GT, Sachdev HS, Chellani H, Rehman AM, Singh V, Arora H et al. Effect of weekly vitamin D supplements on mortality, morbidity, and growth of low birthweight term infants in India up to age 6 months: randomised controlled trial. BMJ 2011; 342: d2975.

189. Marchisio P, Consonni D, Baggi E, Zampiero A, Bianchini S, Terranova L et al. Vitamin D supplementation reduces the risk of acute otitis media in otitis-prone children. Pediatr Infect Dis 2013; 22: 1055-1060.

190. Martineau AR, James WY, Hooper RL, Barnes NC, Jolliffe DA, Greiller CL et al. Vitamin D3 supplementation in patients with chronic obstructive pulmonary disease (ViDiCO): a multicentre, double-blind, randomised controlled trial. The Lancet Respiratory Medicine 2015; 3: 120-130.

191. Martineau AR, MacLaughlin BD, Hooper RL, Barnes NC, Jolliffe DA, Greiller CL et al. Double-blind randomised placebo-controlled trial of bolus-dose vitamin D3 supplementation in adults with asthma (ViDiAs). Thorax 2015; 70: 451.

192. Martineau AR, Hanifa Y, Witt KD, Barnes NC, Hooper RL, Patel M et al. Double-blind randomised controlled trial of vitamin D3 supplementation for the prevention of acute respiratory infection in older adults and their carers (ViDiFlu). Thorax 2015; 70: 953.

193. Denlinger LC, King TS, Cardet JC, Craig T, Holguin F, Jackson DJ et al. Vitamin D Supplementation and the Risk of Colds in Patients with Asthma. Am J Respir Crit Care Med 2016; 193: 634-641.

194. Tachimoto H, Mezawa H, Segawa T, Akiyama N, Ida H, Urashima M. Improved control of childhood asthma with low-dose, short-term vitamin D supplementation: a randomized, double-blind, placebo-controlled trial. Allergy 2016; 71: 1001-1009.

195. Ginde AA, Blatchford P, Breese K, Zarrabi L, Linnebur SA, Wallace JI et al. High-Dose Monthly Vitamin D for Prevention of Acute Respiratory Infection in Older Long-Term Care Residents: A Randomized Clinical Trial. J Am Geriatr Soc 2017; 65: 496-503.

196. Choudhary N, Gupta P. Vitamin D supplementation for severe pneumonia − A randomized controlled trial. Indian Pediatr 2012; 49: 449-454.

197. Spedding S. Vitamin D and depression: a systematic review and meta-analysis comparing studies with and without biological flaws. Nutrients 2014; 6: 1501-1518.

198. Jorde R, Sneve M, Figenschau Y, Svartberg J, Waterloo K. Effects of vitamin D supplementation on symptoms of depression in overweight and obese subjects: randomized double blind trial. J Intern Med 2008; 264: 599-609.

199. Khoraminya N, Tehrani-Doost M, Jazayeri S, Hosseini A, Djazayery A. Therapeutic effects of vitamin D as adjunctive therapy to fluoxetine in patients with major depressive disorder. Aust N Z J Psychiatry 2013; 47: 271-275.

200. Dumville JC, Miles JN, Porthouse J, Cockayne S, Saxon L, King C. Can vitamin D supplementation prevent winter-time blues? A randomised trial among older women. J Nutr Health Aging 2006; 10: 151-153.

201. Arvold DS, Odean MJ, Dornfeld MP, Regal RR, Arvold JG, Karwoski GC et al. Correlation of symptoms with vitamin D deficiency and symptom response to cholecalciferol treatment: a randomized controlled trial. Endocr Pract 2009; 15: 203-212.

202. Lansdowne ATG, Provost SC. Vitamin D_3_ enhances mood in healthy subjects during winter. Psychopharmacology 1998; 135: 319.

203. Vieth R, Kimball S, Hu A, Walfish P. Randomized comparison of the effects of the vitamin D3 adequate intake versus 100 mcg (4000 IU) per day on biochemical responses and the wellbeing of patients. Nutrition Journal 2004; 3: 8.

204. Gloth FM. Vitamin D vs broad spectrum phototherapy in the treatment of seasonal affective disorder. J Nutr Health Aging 1999; 3: 5-7.

205. Zhang M, Robitaille L, Eintracht S, Hoffer LJ. Vitamin C provision improves mood in acutely hospitalized patients. Nutrition 2011; 27: 530-533.

206. Harris S, Dawson-Hughes B. Seasonal mood changes in 250 normal women. Psychiatry Res 1993; 49: 77-87.

207. Bertone-Johnson ER, Powers SI, Spangler L, Larson J, Michael YL, Millen AE et al. Vitamin D Supplementation and Depression in the Womens Health Initiative Calcium and Vitamin D Trial. Am J Epidemiol 2012; 176: 1-13.

208. Belcaro G, Cesarone MR, Cornelli U, Dugall M. MF Afragil(R) in the treatment of 34 menopause symptoms: a pilot study. Panminerva Med 2010; 52: 49-54.

209. Dean AJ, Bellgrove MA, Hall T, Phan WMJ, Eyles DW, Kvaskoff D et al. Effects of Vitamin D Supplementation on Cognitive and Emotional Functioning in Young Adults − A Randomised Controlled Trial. PLoS ONE 2011; 6: e25966.

210. Khajehei M, Abdali K, Parsanezhad ME, Tabatabaee HR. Effect of treatment with dydrogesterone or calcium plus vitamin D on the severity of premenstrual syndrome. Int J Gynaecol Obstet 2009; 105: 158-161.

211. Yalamanchili V, Gallagher JC. Treatment with hormone therapy and calcitriol did not affect depression in older postmenopausal women: no interaction with estrogen and vitamin D receptor genotype polymorphisms. Menopause 2012; 19: 697-703.

212. Li G, Mbuagbaw L, Samaan Z, Falavigna M, Zhang S, Adachi JD et al. Efficacy of vitamin D supplementation in depression in adults: a systematic review. J Clin Endocrinol Metab 2014; 99: 757-767.

213. Li G, Mbuagbaw L, Samaan Z, Zhang S, Adachi JD, Papaioannou A et al. Efficacy of vitamin D supplementation in depression in adults: a systematic review protocol. Syst Rev 2013; 2: 64.

214. Shaffer JAP, Edmondson D, Wasson LT, Falzon L, Homma K, Ezeokoli N et al. Vitamin D Supplementation for Depressive Symptoms: A Systematic Review and Meta-Analysis of Randomized Controlled Trials. Psychosom Med 2014; 76: 190-196.

215. Hogie-Lorenzen, T. (2003) The Relationship Between Depression Levels and Vitamin D Status Among Older Adults in Eastern South Dakota. Madison, South Dakota State University.

216. Mozaffari-Khosravi H, Nabizade L, Yassini-Ardakani SM, Hadinedoushan H, Barzegar K. The Effect of 2 Different Single Injections of High Dose of Vitamin D on Improving the Depression in Depressed Patients With Vitamin D Deficiency: A Randomized Clinical Trial. J Clin Psychopharmacol 2013; 33: 378-385.

217. Gowda U, Mutowo MP, Smith BJ, Wluka AE, Renzaho AMN. Vitamin D supplementation to reduce depression in adults: Meta-analysis of randomized controlled trials. Nutrition 2015; 31: 421-429.

218. Autier P, Gandini S. Vitamin D Supplementation and Total Mortality: A Meta-analysis of Randomized Controlled Trials. Arch Intern Med 2007; 167: 1730-1737.

219. Chapuy MC, Arlot ME, Duboeuf F, Brun J, Crouzet B, Arnaud S et al. Vitamin D3 and calcium to prevent hip fractures in the elderly women. N Engl J Med 1992; 327: 1637-1642.

220. Lips P, Graafmans WC, Ooms ME, Bezemer PD, Bouter LM. Vitamin D supplementation and fracture incidence in elderly persons. A randomized, placebo-controlled clinical trial. Ann Intern Med 1996; 124: 400-406.

221. Baeksgaard L, Andersen KP, Hyldstrup L. Calcium and vitamin D supplementation increases spinal BMD in healthy, postmenopausal women. Osteoporos Int 1998; 8: 255-260.

222. Krieg MA, Jacquet AF, Bremgartner M, Cuttelod S, Thiébaud D, Burckhardt P. Effect of Supplementation with Vitamin D3 and Calcium on Quantitative Ultrasound of Bone in Elderly Institutionalized Women: A Longitudinal Study. Osteoporos Int 1999; 9: 483-488.

223. Chapuy MC, Pamphile R, Paris E, Kempf C, Schlichting M, Arnaud S et al. Combined calcium and vitamin D3 supplementation in elderly women: confirmation of reversal of secondary hyperparathyroidism and hip fracture risk: the Decalyos II study. Osteoporos Int 2002; 13: 257-264.

224. Meyer HE, Smedshaug GB, Kvavik ELIS, Falch JA, Tverdal A, Pedersen JI. Can Vitamin D Supplementation Reduce the Risk of Fracture in the Elderly? A Randomized Controlled Trial. J Bone Min Res 2002; 17: 709-715.

225. Latham NK, Anderson CS, Lee A, Bennett DA, Moseley A, Cameron ID. A randomized, controlled trial of quadriceps resistance exercise and vitamin D in frail older people: the Frailty Interventions Trial in Elderly Subjects (FITNESS). J Am Geriatr Soc 2003; 51: 291-299.

226. Harwood RH, Sahota O, Gaynor K, Masud T, Hosking DJ. A randomised, controlled comparison of different calcium and vitamin D supplementation regimens in elderly women after hip fracture: The Nottingham Neck of Femur (NONOF) Study. Age Ageing 2004; 33: 45-51.

227. Meier C, Woitge HW, Witte K, Lemmer B, Seibel MJ. Supplementation With Oral Vitamin D3 and Calcium During Winter Prevents Seasonal Bone Loss: A Randomized Controlled Open-Label Prospective Trial. J Bone Min Res 2004; 19: 1221-1230.

228. Porthouse J, Cockayne S, King C, Saxon L, Steele E, Aspray T et al. Randomised controlled trial of calcium and supplementation with cholecalciferol (vitamin D3) for prevention of fractures in primary care. BMJ 2005; 330: 1003.

229. Flicker L, MacInnis RJ, Stein MS, Scherer SC, Mead KE, Nowson CA et al. Should older people in residential care receive vitamin D to prevent falls? Results of a randomized trial. J Am Geriatr Soc 2005; 53: 1881-1888.

230. Brohult J, Jonson B. Effects of large doses of calciferol on patients with rheumatoid arthritis. A double-blind clinical trial. Scand J Rheumatol 1973; 2: 173-176.

231. Grove O, Halver B. Relief of osteoporotic backache with fluoride, calcium, and calciferol. Acta Med Scand 1981; 209: 469-471.

232. Campbell AJ, Robertson MC, Grow SJL, Kerse NM, Sanderson GF, Jacobs RJ et al. Randomised controlled trial of prevention of falls in people aged ΓëÑ75 with severe visual impairment: the VIP trial. BMJ 2005; 331: 817.

233. Lyons R, Johansen A, Brophy S, Newcombe R, Phillips C, Lervy B et al. Preventing fractures among older people living in institutional care: a pragmatic randomised double blind placebo controlled trial of vitamin D supplementation. Osteoporos Int 2007; 18: 811-818.

234. Broe KE, Chen TC, Weinberg J, Bischoff-Ferrari HA, Holick MF, Kiel DP. A higher dose of vitamin d reduces the risk of falls in nursing home residents: a randomized, multiple-dose study. J Am Geriatr Soc 2007; 55: 234-239.

235. Burleigh E, McColl J, Potter J. Does vitamin D stop inpatients falling? A randomised controlled trial. Age Ageing 2007; 36: 507-513.

236. Bjorkman M, Sorva A, Risteli J, Tilvis R. Vitamin D supplementation has minor effects on parathyroid hormone and bone turnover markers in vitamin D deficient bedridden older patients. Age Ageing 2008; 37: 25-31.

237. Wejse C, Gomes VF, Rabna P, Gustafson P, Aaby P, Lisse IM et al. Vitamin D as Supplementary Treatment for Tuberculosis - A Double-blind Randomized Placebo-controlled Trial. Am J Respir Crit Care Med 2009; 179: 843-850.

238. Bjelakovic G, Gluud LL, Nikolova D, Whitfield K, Wetterslev J, Simonetti RG et al. Vitamin D supplementation for prevention of mortality in adults. Cochrane Database Syst Rev 2011; CD007470.

239. Cordless D, DAWSON E, FRASER F, ELLIS M, Evans SJ, PERRY JD et al. Do vitamin D supplements improve the physical capabilities of elderly hospital patients? Age Ageing 1985; 14: 76-84.

240. Ooms ME, Roos JC, Bezemer PD, van der Vijgh WJ, Bouter LM, Lips P. Prevention of bone loss by vitamin D supplementation in elderly women: a randomized double-blind trial. J Clin Endocrinol Metab 1995; 80: 1052-1058.

241. Sato Y, Maruoka H, Oizumi K. Amelioration of Hemiplegia-Associated Osteopenia More Than 4 Years After Stroke by 1α-Hydroxyvitamin D3 and Calcium Supplementation. Stroke 1997; 28: 736-739.

242. Sato Y, Manabe S, Kuno H, Oizumi K. Amelioration of osteopenia and hypovitaminosis D by 1alpha-hydroxyvitamin D3 in elderly patients with Parkinson´s disease. J Neurol Neurosurg Psychiatry 1999; 66: 64-68.

243. Sato Y, Kuno H, Kaji M, Saruwatari N, Oizumi K. Effect of ipriflavone on bone in elderly hemiplegic stroke patients with hypovitaminosis D. Am J Phys Med Rehabil 1999; 78: 457-463.

244. Bischoff HA, Stahelin HB, Dick W, Akos R, Knecht M, Salis C et al. Effects of vitamin D and calcium supplementation on falls: a randomized controlled trial. J Bone Miner Res 2003; 18: 343-351.

245. Cooper L, Clifton-Bligh PB, Nery ML, Figtree G, Twigg S, Hibbert E et al. Vitamin D supplementation and bone mineral density in early postmenopausal women. Am J Clin Nutr 2003; 77: 1324-1329.

246. Larsen ER, Mosekilde L, Foldspang A. Vitamin D and calcium supplementation prevents osteoporotic fractures in elderly community dwelling residents: a pragmatic population-based 3-year intervention study. J Bone Miner Res 2004; 19: 370-378.

247. Dukas L, Bischoff HA, Lindpaintner LS, Schacht E, Birkner-Binder D, Damm TN et al. Alfacalcidol reduces the number of fallers in a community-dwelling elderly population with a minimum calcium intake of more than 500 mg daily. J Am Geriatr Soc 2004; 52: 230-236.

248. Sato Y, Iwamoto J, Kanoko T, Satoh K. Low-Dose Vitamin D Prevents Muscular Atrophy and Reduces Falls and Hip Fractures in Women after Stroke: A Randomized Controlled Trial. Cerebrovasc Dis 2005; 20: 187-192.

249. Moschonis G, Manios Y. Skeletal site-dependent response of bone mineral density and quantitative ultrasound parameters following a 12-month dietary intervention using dairy products fortified with calcium and vitamin D: the Postmenopausal Health Study. Br J Nutr 2006; 96: 1140-1148.

250. Law M, Withers H, Morris J, Anderson F. Vitamin D supplementation and the prevention of fractures and falls: results of a randomised trial in elderly people in residential accommodation. Age Ageing 2006; 35: 482-486.

251. Smith H, Anderson F, Raphael H, Maslin P, Crozier S, Cooper C. Effect of annual intramuscular vitamin D on fracture risk in elderly men and women--a population-based, randomized, double-blind, placebo-controlled trial. Rheumatology (Oxford) 2007; 46: 1852-1857.

252. Aspray TJ, Francis RM. Vitamin D deficiency can old age learn from childhood? Age Ageing 2008; 37: 6-7.

253. Chel V, Wijnhoven H, Smit J, Ooms M, Lips P. Efficacy of different doses and time intervals of oral vitamin D supplementation with or without calcium in elderly nursing home residents. Osteoporos Int 2008; 19: 663-671.

254. Brunner RL, Cochrane B, Jackson RD, Larson J, Lewis C, Limacher M et al. Calcium, Vitamin D Supplementation, and Physical Function in the Women's Health Initiative. J Am Diet Assoc 2008; 108: 1472-1479.

255. Lips P, Binkley N, Pfeifer M, Recker R, Samanta S, Cohn DA et al. Once-weekly dose of 8400 IU vitamin D3 compared with placebo: effects on neuromuscular function and tolerability in older adults with vitamin D insufficiency. Am J Clin Nutr 2010; 91: 985-991.

256. Käkkäinen M, Tuppurainen M, Salovaara K, Sandini L, Rikkonen T, Sirola J et al. Effect of calcium and vitamin D supplementation on bone mineral density in women aged 65-71 years: a 3-year randomized population-based trial (OSTPRE-FPS). Osteoporos Int 2010; 21: 2047-2055.

257. Rejnmark L, Avenell A, Masud T, Anderson F, Meyer HE, Sanders KM et al. Vitamin D with Calcium Reduces Mortality: Patient Level Pooled Analysis of 70,528 Patients from Eight Major Vitamin D Trials. J Clin Endocrin Metab 2012; 97: 2670-2681.

258. Chapuy MC, Arlot ME, Delmans PD, Meunier PJ. Effect of calcium and cholecalciferol treatment for three years on hip fractures in elderly women. BMJ 1994; 308: 1081-1082.

259. LaCroix AZ, Kotchen J, Anderson G, Brzyski R, Cauley JA, Cummings SR et al. Calcium Plus Vitamin D Supplementation and Mortality in Postmenopausal Women: The Women's Health Initiative Calcium-Vitamin D Randomized Controlled Trial. The Journals of Gerontology Series A: Biological Sciences and Medical Sciences 2009; 64: 559-567.

260. Salovaara K, Tuppurainen M, Karkkainen M, Rikkonen T, Sandini L, Sirola J et al. Effect of vitamin D(3) and calcium on fracture risk in 65- to 71-year-old women: a population-based 3-year randomized, controlled trial--the OSTPRE-FPS. J Bone Miner Res 2010; 25: 1487-1495.

261. Zheng Y, Zhu J, Zhou M, Cui L, Yao W, Liu Y. Meta-Analysis of Long-Term Vitamin D Supplementation on Overall Mortality. PLoS ONE 2013; 8: e82109.

262. Bolland MJ, Grey A, Gamble GD, Reid IR. Calcium and vitamin D supplements and health outcomes: a reanalysis of the Women's Health Initiative (WHI) limited-access data set. The American Journal of Clinical Nutrition 2011; 94: 1144-1149.

263. Punthakee Z, Bosch J, Dagenais G, Diaz R, Holman R, Probstfield J et al. Design, history and results of the Thiazolidinedione Intervention with vitamin D Evaluation (TIDE) randomised controlled trial. Diabetologia 2012; 55: 36-45.

264. Wasse H, Huang R, Long Q, Singapuri S, Raggi P, Tangpricha V. Efficacy and safety of a short course of very-high-dose cholecalciferol in hemodialysis. Am J Clin Nutr 2012; 95: 522-528.

265. Avenell A, Mak JC, O'Connell D. Vitamin D and vitamin D analogues for preventing fractures in post-menopausal women and older men. Cochrane Database Syst Rev 2014; 4: CD000227.

266. Gallagher JC. Metabolic effects of synthetic calcitriol (Rocaltrol) in the treatment of postmenopausal osteoporosis. Metabolism 1990; 39: 27-29.

267. Tilyard MW, Spears GFS, Thomson J, Dovey S. Treatment of Postmenopausal Osteoporosis with Calcitriol or Calcium. N Engl J Med 1992; 326: 357-362.

268. Jackson RD, LaCroix AZ, Cauley JA, McGowan J. The women's health initiative calcium-vitamin D trial: overview and baseline characteristics of participants. Ann Epidemiol 2003; 13: S98-S106.

269. Bjelakovic G, Gluud LL, Nikolova D, Whitfield K, Wetterslev J, Simonetti RG et al. Vitamin D supplementation for prevention of mortality in adults. Cochrane Database of Systematic Reviews 2014.

270. Cherniack EP, Florez HJ, Hollis BW, Roos BA, Troen BR, Levis S. The Response of Elderly Veterans to Daily Vitamin D3 Supplementation of 2,000 IU: A Pilot Efficacy Study. J Am Geriatr Soc 2011; 59: 286-290.

271. Chowdhury R, Kunutsor S, Vitezova A, Oliver-Williams C, Chowdhury S, Kiefte-de-Jong JC et al. Vitamin D and risk of cause specific death: systematic review and meta-analysis of observational cohort and randomised intervention studies. BMJ 2014; 348: g1903.

272. Beer TM, Ryan CW, Venner PM, Petrylak DP, Chatta GS, Ruether JD et al. Double-Blinded Randomized Study of High-Dose Calcitriol Plus Docetaxel Compared With Placebo Plus Docetaxel in Androgen-Independent Prostate Cancer: A Report From the ASCENT Investigators. J Clin Oncol 2007; 25: 669-674.

273. Zheng YT, Cui QQ, Hong YM, Yao WG. A Meta-Analysis of High Dose, Intermittent Vitamin D Supplementation among Older Adults. PLoS ONE 2015; 10: e0115850.
